# Design of customized coronavirus receptors

**DOI:** 10.1038/s41586-024-08121-5

**Published:** 2024-10-30

**Authors:** Peng Liu, Mei-Ling Huang, Hua Guo, Matthew McCallum, Jun-Yu Si, Yuan-Mei Chen, Chun-Li Wang, Xiao Yu, Lu-Lu Shi, Qing Xiong, Cheng-Bao Ma, John E. Bowen, Fei Tong, Chen Liu, Ye-hui Sun, Xiao Yang, Jing Chen, Ming Guo, Jing Li, Davide Corti, David Veesler, Zheng-Li Shi, Huan Yan

**Affiliations:** 1 State Key Laboratory of Virology, College of Life Sciences, TaiKang Center for Life and Medical Sciences, Wuhan University; Wuhan, Hubei, China; 2 Key Laboratory of Virology and Biosafety, Wuhan Institute of Virology, Chinese Academy of Sciences; Wuhan, China; 3 Guangzhou Laboratory, Guangzhou International Bio Island; Guangzhou, China; 4 Department of Biochemistry, University of Washington; Seattle, WA 98195, USA; 5 Howard Hughes Medical Institute, University of Washington; Seattle, WA 98195, USA; 6 Humabs BioMed SA, subsidiary of Vir Biotechnology, Bellinzona, Switzerland

**Keywords:** receptors, viral entry, coronaviruses, infection model, customized viral receptor

## Abstract

Although coronaviruses use diverse receptors, the characterization of coronaviruses without known receptors has been impeded by the lack of infection models^1,2^. Here, we introduce an innovative strategy to engineer functional customized viral receptors (CVRs). The modular design relies on building artificial receptor scaffolds (ARS) comprising various modules and generating specific viral binding domains (VBD). We reveal key factors for CVRs to functionally mimic native receptors by facilitating spike proteolytic cleavage, membrane fusion, pseudovirus entry, and propagation for various coronaviruses. We delineate functional SARS-CoV-2 spike receptor binding sites for CVR design and elucidate the mechanism of cell entry promoted by the N-terminal domain (NTD)-targeting S2L20-CVR. We generated CVR-expressing cells for 12 representative coronaviruses from six subgenera, most of which lacking known receptors, and show that a pan-sarbecovirus CVR supports propagation of a replication-competent HKU3 pseudovirus and of authentic RsHuB2019A^3^. Using an HKU5-specific CVR, we successfully rescued wild-type and ZsGreen-Hibit-incorporated HKU5-1 (LMH03f) and isolated an HKU5 strain from bat samples. Our study demonstrates the potential of the CVR strategy for establishing native receptor-independent infection models, paving the way for studying viruses challenging to culture due to the lack of susceptible target cells.

## Introduction

The *Coronaviridae* family encompasses hundreds of viruses categorized into four genera, α-, β-, γ- and δ-coronaviruses. Human β-coronaviruses have caused three significant outbreaks in the 21^st^ century, highlighting the zoonotic risks associated with animal coronaviruses that are poorly studied, primarily infecting bats^3,4^.

Coronavirus entry is mediated by the trimeric spike glycoprotein (S)^5–7^, which can remain intact or undergo cleavage at the S_1_/S_2_ cleavage site, yielding S_1_ and S_2_ subunits^7^. The S_1_ subunit engages specific receptors, leading to conformational changes that trigger membrane fusion mediated by the S_2_ subunit. Activation of the S_2_ fusion machinery leads to exposure and proteolysis of the S_2_’ cleavage site situated immediately upstream of the fusion peptide (FP) and large-scale conformational changes from the prefusion conformation to the post-fusion conformation. Except for mouse hepatitis virus (MHV), most coronaviruses use their C-terminal domain (CTD, or domain B) of the S_1_ subunit as receptor binding domains (RBD), adopting either “down” or “up” conformations^8^, the latter state enabling receptor engagement^8,9^. SARS-CoV-2 recognizes the protease (head) domain of human angiotensin-converting enzyme 2 (ACE2) through the receptor-binding motif (RBM). ACE2-induced conformational changes expose the S2’ site, promoting proteolytic cleavage by the cell surface transmembrane serine protease 2 (TMPRSS2), the endosomal cysteine proteases cathepsin L or other proteases^10^.

Efforts in the past decades have led to the identification of five widely acknowledged protein entry receptors for coronaviruses: ACE2, Aminopeptidase N (APN), Dipeptidyl peptidase-4 (DPP4), Carcinoembryonic antigen-related cell adhesion molecule 1a (CEACAM1a), and TMPRSS2^2,11,12^. ACE2 is an entry receptor for various coronaviruses, including NL63, SARS-CoV-1, SARS-CoV-2, and several clades of bat-borne sarbecoviruses and merbecoviruses^13–16^. CryoEM analysis of human ACE2 (hACE2) demonstrated a dimerized structure, with a direct engagement of the head domain by the SARS-CoV-2 RBD, particularly the α1 and α2-helix and the loop connecting the β3- and β4-sheets^9^. However, the contribution of other ACE2 regions to receptor function remains unknown. Several alternative receptors or coreceptors for SARS-CoV-2 have been reported, but their entry-supporting efficiencies are generally low compared with that of ACE2^17,18^. Numerous bat coronaviruses have no known receptors, thereby limiting our ability to study them and to develop countermeasures^1,4^.

Functional entry receptors are crucial for developing infection models and inhibition assays, while the identification of native receptors is time-consuming with no guarantee of success. To address this unmet need, an alternative approach of establishing infection models independently of the identification of native receptors is awaiting exploration, with few attempts on MHV reported for this purpose^19^. The design of functional coronavirus receptors is impeded by the lack of knowledge regarding the critical sequence and structural requirements to induce spike conformational changes leading to cell entry. Studies focusing on SARS-CoV-2 or MERS-CoV have elucidated several scenarios of ACE2 or DPP4-independent entry *in vitro*, either by alternative receptors, antibody-dependent FcγR-mediated entry, or membrane-anchored antibodies, either in a productive or nonproductive manner^20–23^. These findings indicate that the interactions between coronaviruses and cognate receptors are dispensable for entry, making it possible to design receptors without prior knowledge of the native receptors.

In this study, we built receptors with customized specificity by grafting viral binding domains on various transmembrane scaffolds, demonstrating that ACE2 can be entirely substituted to promote sarbecovirus entry into cells. By deciphering the key factors affecting receptor functionality, we developed a generally applicable modular design strategy to build functional customized viral receptors (CVRs) for supporting productive viral entry and demonstrate the strengths of this strategy for various applications in virology.

## Results

### Modular design of CVR

**Extended Data Fig. 1 |:**
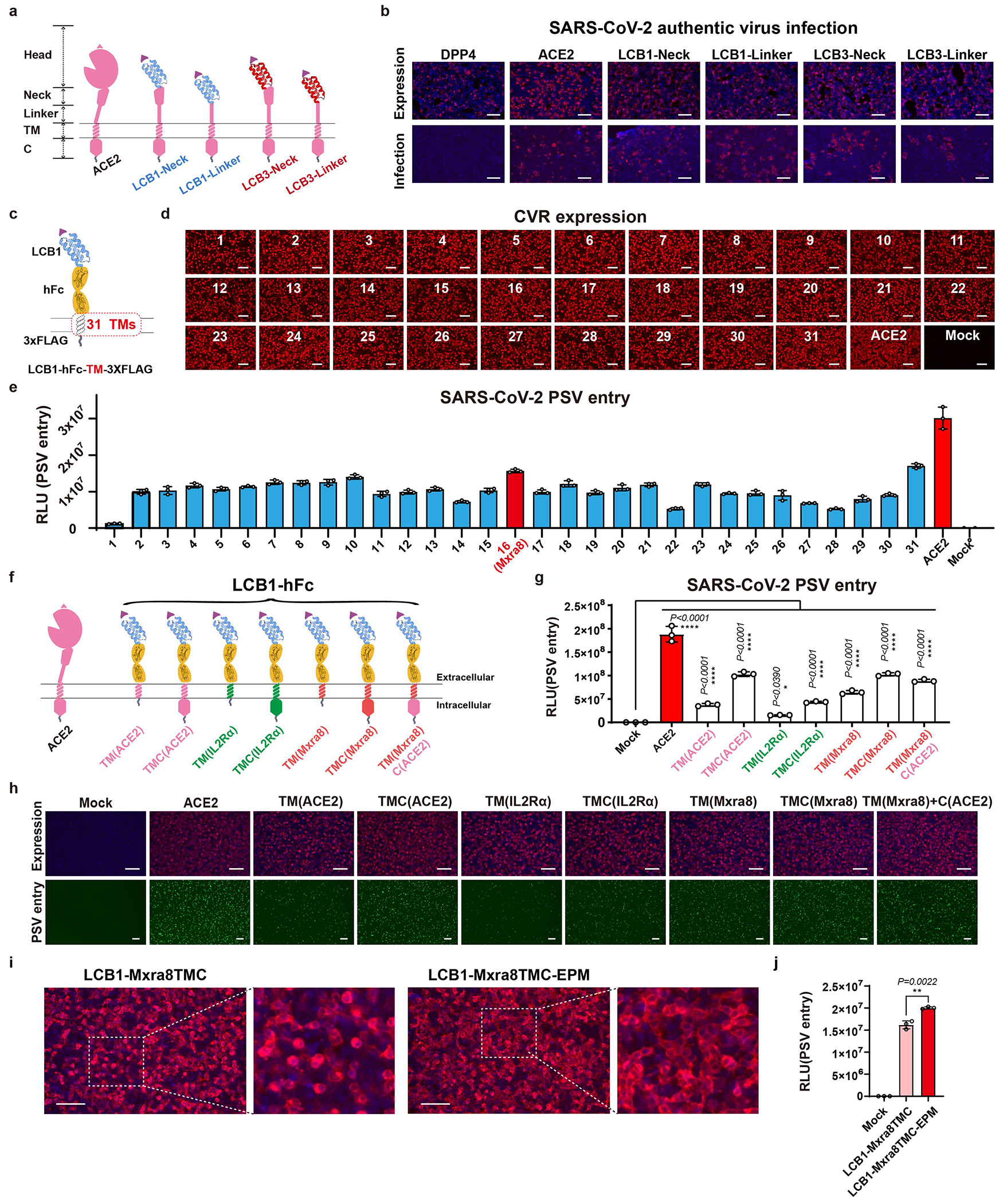
Development and optimization of a modular design strategy for CVR with a type I transmembrane topology. **a,** Schematic illustration of the four miniprotein-based CVRs. **b,** Immunofluorescence analysis of ACE2/CVRs expression and authentic SARS-CoV-2 infection in HEK293T cells stably expressing the receptors. Upper: Receptor expression examined by C-terminal fused 3×FLAG tags. Lower: SARS-CoV-2 infection efficiency as indicated by intracellular N proteins at 24 hpi. Data representative of two independent authentic SARS-CoV-2 infection assays with similar results. **c,** Cartoon illustrating the framework of the CVRs for TM evaluation. **d,** Immunofluorescence analysis of the expression of the 31 CVRs in HEK293T cells by detecting the C-terminal fused 3×FLAG tags. **e,** SARS-CoV-2 PSV entry efficiency promoted by CVRs carrying different TMs. The detailed information on the TMs is summarized in Supplementary Table 2. Data are presented as mean ± s.d. (n = 3 independently infected cells), representative of two independent experiments with similar results. **f,** Schematic diagram showing the LCB1-based CVRs with indicated TM or TMC substitutions. **g, h,** SARS-CoV-2 PSV entry in HEK293T cells transiently expressing the indicated CVRs examined by RLU(**g**) or GFP (**h**). Data are presented as mean ± s.d. (n = 3 independently infected cells), Statistical analysis was performed using One-way ANOVA analysis followed by Dunnett’s test. Data represented were performed in at least two independent experiments with similar results. **i,** Immunofluorescence analysis of the subcellular distribution of LCB1-Mxra8 TMC-based CVRs with or without EPM transiently expressed in HEK293T cells. The white dashed boxes highlight the cell surface distribution with a higher magnification. **j**, SARS-CoV-2 PSV entry efficiency in HEK293T cells transiently expressing CVRs with or without EPM. Data are mean ± s.d. (n = 3 independently infected cells) and analyzed with unpaired two-tailed Student’s *t*-tests, representative of two independent experiments with similar results. Scale bars: 100 μm in **b**, **d**, **h,** and **i**. *P<0.05, **P<0.01, ****P<0.0001.

To understand the contribution of different domains of human ACE2 (hACE2) to its receptor function, we first assessed whether we could replace the hACE2 head domain with a computationally designed SARS-CoV-2-targeting miniprotein while maintaining its receptor function. Four hACE2 chimeric proteins were created, with the head domain or the head and neck domains replaced with one of the previously reported miniproteins designated LCB1 and LCB3^24^. Using authentic SARS-CoV-2 infection assays, we demonstrated that these chimeric proteins effectively supported viral infection (Extended Data Fig. 1a,b).

**Fig. 1 |:**
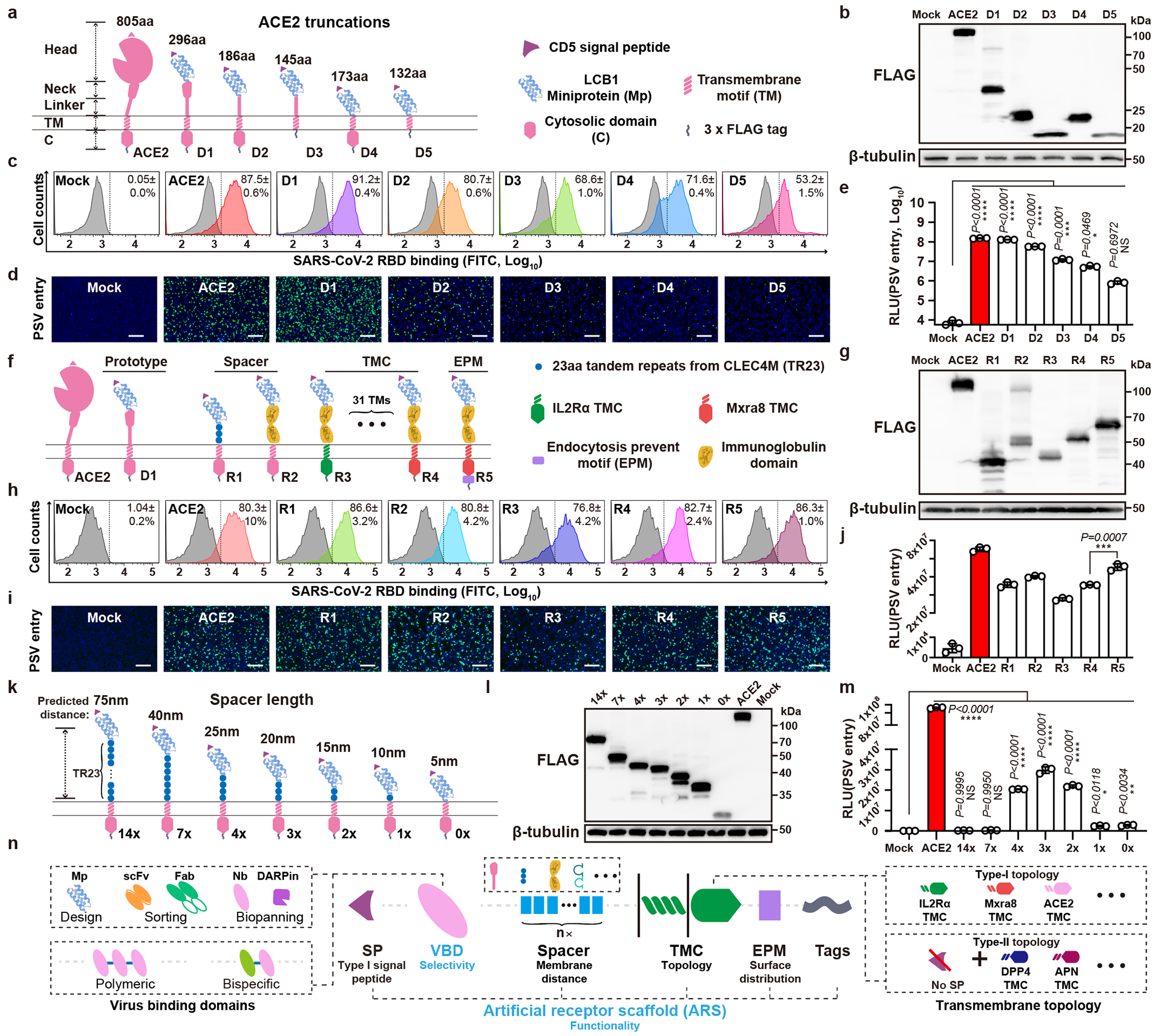
Modular design of customized viral receptors (CVRs) for efficient coronavirus entry. **a-e,** Dissecting the importance of ACE2 sequences for its viral receptor function. **a,** Schematic representation illustrates the LCB1-ACE2 chimera with stepwise truncated ACE2 sequences. Protein expression levels (**b**) and SARS-CoV-2 RBD binding efficiency (**c**) in HEK293T cells transiently expressing the specified chimeras. SARS-CoV-2 pseudovirus (PSV) entry in HEK293T cells expressing each chimera was demonstrated by GFP (**d**) or RLU (**e**), respectively. Scale bars: 200 μm. **f-j,** Functionality of chimeric receptors with remaining ACE2 sequences substituted by domains from other proteins. Schematic representation (**f**) delineates CVRs carrying exogenous spacer, transmembrane and cytosolic domain (TMC), and endocytosis-prevention motif (EPM) sequences. The CVR expression (**g**), SARS-CoV-2 RBD-mFc binding (**h**), and PSV entry (**i, j**) efficiencies in HEK293T transiently expressing the indicated receptors were shown. Scale bars: 200 μm. **k-m**, The impact of spacer length on CVR receptor function. Schematic representation (**k**) illustrates CVRs with various TR23 tandem repeats, displaying predicted spacer length. CVR expression (**l**) and SARS-CoV-2 PSV entry efficiency (**m**) were evaluated in HEK293T cells transiently expressing the indicated CVRs. **n,** Schematic illustration of the modular design strategy for CVRs. RLU: relative light units. Data representative of at least two independent transfections (Western blot for expression) and functional assays (RBD binding and PSV entry) with similar results for **a**-**e**, **f**-**j**, and **k**-**m**, respectively. Data are presented as mean ± s.d., biological triplicate of bound cells (dashed lines denote thresholds for positive binding) for **c** and **h,** and biological triplicates of infected cells for **e**, **j**, and **m**. One-way ANOVA analysis followed by Dunnett’s test for **e** and **j;** unpaired two-tailed Student’s *t*-tests for **m**. *P<0.05, **P<0.01, ***P<0.001, ****P<0.0001; NS, not significant (P>0.05).

We found even if we gradually reduce the remaining parts of ACE2 (Fig. 1a), these modified receptors maintained comparable binding efficiency to SARS-CoV-2 RBD (Fig. 1b,c). However, their ability to promote pseudovirus entry decreased concurrently with the shortening of the ACE2 anchor. The shortest 132aa protein maintained detectable receptor function but at a much lower level than that of full-length ACE2 (approximately 0.5%) (Fig. 1d,e and Supplementary Table 1). We subsequently designed modified receptors comprising parts from other viral or immune receptors (R1-R4), along with the addition of a receptor endocytosis-prevention motif (EPM) to enhance surface distribution (R5) (Fig. 1f)^25^. All chimeric receptors expressed well and bound to RBD efficiently (Fig. 1g–h). Particularly, the chimeric protein with the ACE2 neck domain substituted with triple (3×) 23aa tandem repeats (TR23) from CLEC4M or human IgG Fc (hFc) supported efficient entry (Fig. 1i–j). Further substituting the remaining sequence with IL2Rα corresponding sequences maintained similar entry efficiency, suggesting that ACE2 is entirely replaceable (Fig. 1i,j). Among 31 tested transmembrane (TM) segments and several cytosolic domains from various receptors, the transmembrane and cytosolic domain (TMC) from the matrix remodeling-associated protein 8 (Mxra8) exhibited the best performance (Extended Data Fig. 1c–h and Supplementary Table 2). Constructs with EPM showed improved cell surface localization and improved entry-supporting efficiency (Fig. 1i–j, Extended Data Fig. 1i,j).

**Extended Data Fig. 2 |:**
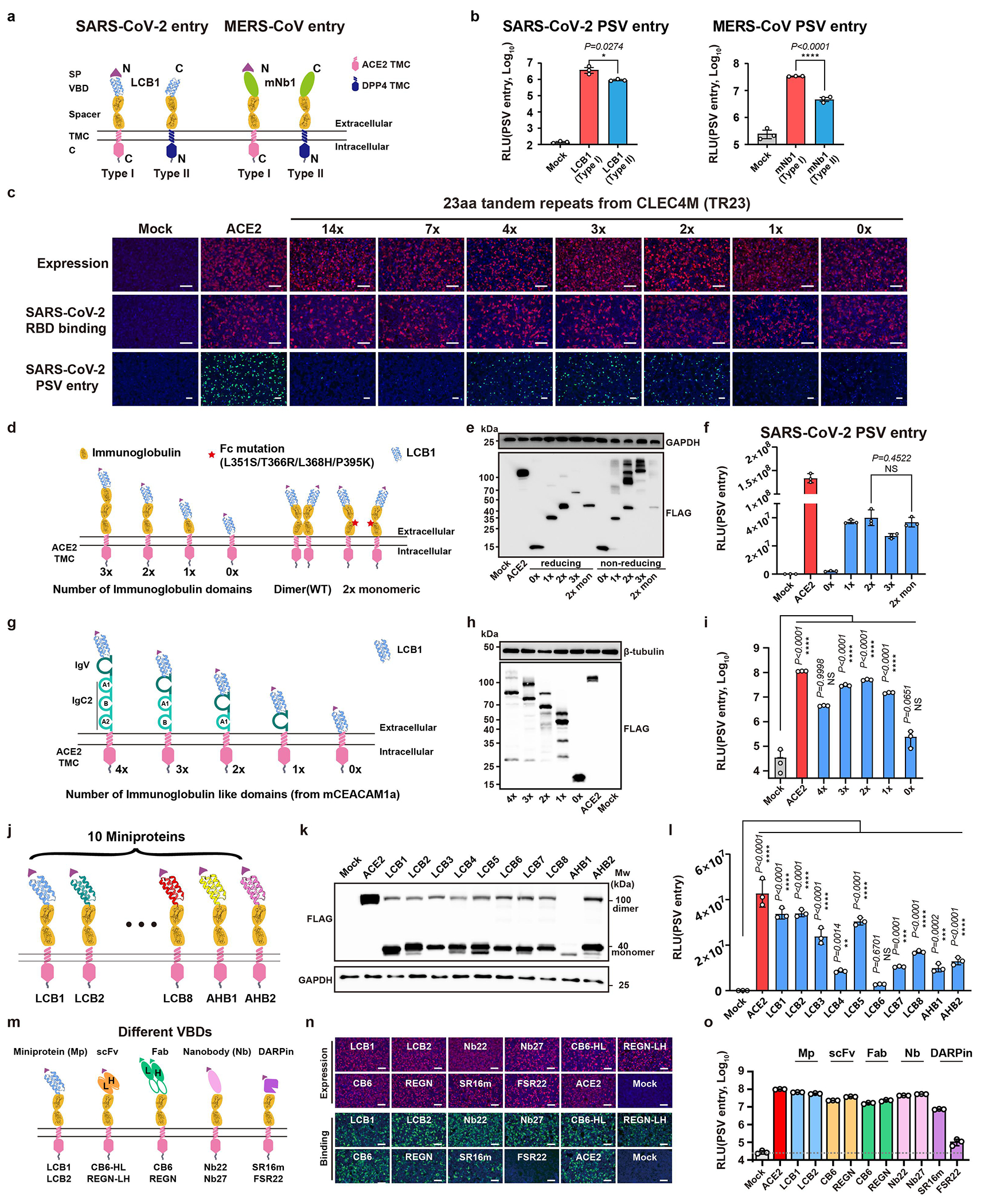
Exploring factors that contribute to the receptor function of CVRs with different topologies or modules. **a,** Schematic diagram showing CVRs carrying LCB1 or mNb1 displayed in either type I or type Ⅱ transmembrane topology. **b,** Evaluation of SARS-CoV-2 or MERS-CoV PSV entry efficiency supported by the indicated CVRs with different transmembrane topologies in HEK293T cells. Data are mean ± s.d. of biological triplicates examined over three independent infection assays. Unpaired two-tailed Student’s *t*-tests. **c,** Assessment of CVR expression, SARS-CoV-2 RBD-mFc binding, and PSV entry efficiency supported by the CVRs carrying varying copies of TR23 repeats transiently expressed in HEK293T cells. Data are representative of three independent experiments. Scale bars: 100 μm. **d,** Schematic representation of the CVRs carrying different numbers of immunoglobulin (Ig) domains (left) or an Fc mutant with abolished dimerization ability. **e,** Western blot analysis of CVRs expression in HEK293T cells under either reducing or non-reducing conditions, respectively. **f,** Assessment of SARS-CoV-2 PSV entry efficiency in HEK293T cells transiently expressing the indicated CVRs. Data are mean ± s.d. (n = 3 independently infected cells.) and analyzed by unpaired two-tailed Student’s *t*-tests. **g,** Schematic representation of the CVRs carrying different numbers of Ig-like domains (left) from mCEACAM1a. **h,** Western blot analysis of CVRs expression in HEK293T cells. **i,** SARS-CoV-2 PSV entry efficiency in HEK293T cells transiently expressing the indicated CVRs. Data are mean ± s.d. (n = 3 independently infected cells.). One-way ANOVA analysis followed by Dunnett’s test. **j**, Schematic representation of the CVRs carrying different SARS-CoV-2 RBD targeting miniproteins. **k, l**, Expression (**k**) and SARS-CoV-2 entry-supporting (**l**) ability of different CVRs in 293T cells. Data are mean ± s.d. (n = 3 independently infected cells). One-way ANOVA analysis followed by Dunnett’s test. **m**, Schematic diagram showing CVRs carrying different types of VBDs, the two representative VBDs for each type are indicated. **n,** Immunofluorescence analyzing the expression of the indicated CVRs transiently expressed in HEK293T cells by detecting the C-terminal fused 3×FLAG tags, and the SARS-CoV-2 RBD binding. Scale bars: 100 μm. **o,** PSV entry are supported by indicated CVRs transiently expressed in HEK293T cells. Data are mean ± s.d. (n = 3 independently infected cells). Data representative of two independent transfections, expression verification, and infection assays with similar results for **d**-**f**, **g**-**i**, **j**-**l,** and **m**-**o**, respectively. *P<0.05, **P<0.01, ***P<0.001, ****P<0.0001; NS, Not significant (*P*>0.05).

Moreover, constructs with a type-II transmembrane topology efficiently supported SARS-CoV-2 and MERS-CoV entry, indicating the feasibility of generating receptors with different transmembrane topologies (Extended Data Fig. 2a,b). We then explored the impact of spacer length and oligomerization on receptor function by testing spacers with various copies of TR23 tandem repeats or immunoglobulin-like domains from human IgG or mCEACAM1a (Fig. 1k–l, and Extended Data Fig. 2c–i). Results indicated the triple TR23 or two immunoglobulin (Ig) or Ig-like domains represent the optimal spacers while abolishing dimerization by Fc mutants has no significant impact on receptor function (Fig. 1m and Extended Data Fig. 2c–i)^26^.

**Extended Data Fig. 3 |:**
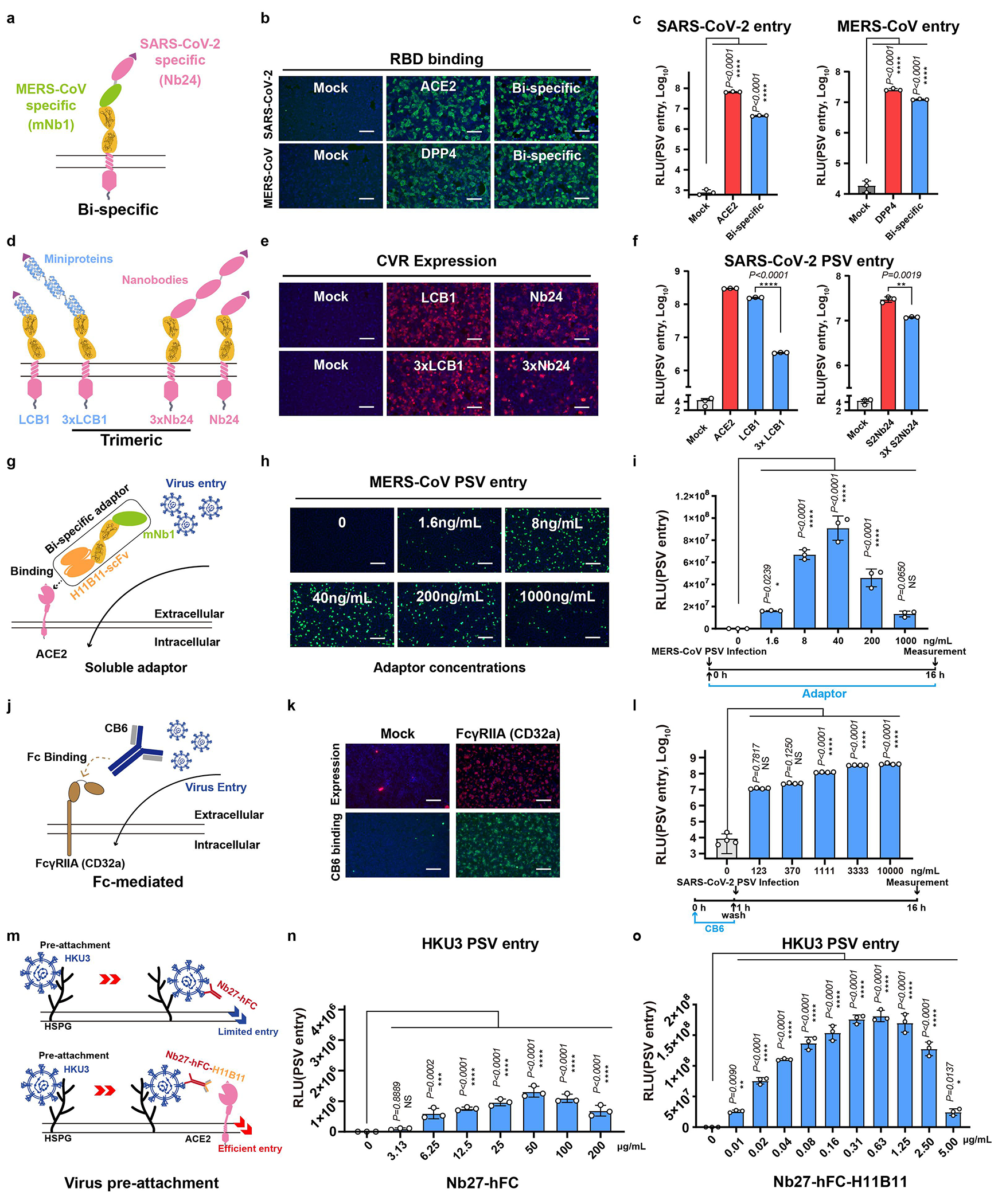
Investigating receptor function of CVRs with VBDs connected in various ways. **a-c,** Illustration (**a**), RBD binding efficiency (**b**), and PSV entry-supporting efficiency (**c**) of a SARS-CoV-2/MERS-CoV bi-specific CVR transiently expressed in HEK293T cells. Data are presented as mean ± s.d. (biological triplicates of infected cells), representative of three independent experiments with similar results. Unpaired two-tailed Student’s *t*-test. **d-f,** Illustration (**d**), expression (**e**), and PSV entry-supporting efficiencies (**f**) of CVRs carrying single or trimeric VBD. Data are mean ± s.d. (biological triplicates of infected cells), analyzed by unpaired two-tailed Student’s *t*-test. Representative of two independent experiments. **g-i,** Schematic illustration of bispecific adapter protein (**g**) and MERS-CoV PSV entry efficiency in BHK-21-hACE2 cells in the presence of indicated concentrations of adaptor proteins (h11B11-mNb1) throughout the infection. Entry efficiency is examined by GFP intensity (**h**) or RLU (**i**). Data are mean ± s.d. (biological triplicates of infected cells), representative of three independent infection assays. **j-l**, Schematic illustration of FcγR (CD32a) mediated antibody-dependent coronavirus entry (**j**). CD32a expression, antibody (CB6) binding (**k**), and SARS-CoV-2 PSV entry (**l**) into HEK293T-CD32a cells pretreated with indicated concentration (con.) of the CB6 antibodies. Data are mean ± s.d. (n=4 biologically independent cells), examined over two independent experiments. **m-o,** Entry of pre-attached PSV promoted by soluble neutralizing antibody (Nb27-hFc) or bi-specific neutralizing antibody with membrane-associating ability (Nb27-hFc-h11B11). Schematic illustration (**m**), Nb27-hFc promoted entry (**n**), and Nb27-hFc-h11B11 promoted PSV entry (**o**) in Caco2 cells with virus pre-attachment by 1500 rpm centrifugation at 4°C for 1 hour. Data are mean ± s.d. (biological triplicates of infected cells). Data are representative of three independent infection assays. Scale bars: 100 μm for **b**, **e**, and **k**, and 200 μm for **h**. One-way ANOVA analysis followed by Dunnett’s test for **i**, **l**, **n,** and **o**. *P<0.05, **P<0.01, ***P<0.001, ****P<0.0001; NS, Not significant (*P*>0.05).

Different types of SARS-CoV-2 RBD-targeting viral binding domains (VBDs) were tested for receptor grafting, including designed helical frameworks, designed ankyrin repeat proteins (DARPins), nanobodies, scFv, and Fab (Extended Data Fig. 2j,m). We found that all these VBD types were functional, with nanobodies being preferred due to their relatively small size, single-chain nature, and compatibility with bio-panning (Extended Data Fig. 2k–l,n–o). We also demonstrated the functionality of a bi-specific receptor carrying two VBDs of different specificity, and tandem trimeric VBDs (Extended Data Fig. 3a–f). Additionally, we show entry facilitated by soluble receptor adapters connecting viral RBD and ACE2 or FcγRIIa, respectively (Extended Data Fig. 3g–l). In contrast, soluble antibodies without membrane anchoring ability are unable to efficiently promote viral entry, even when viruses are pre-attached to the cells (Extended Data Fig. 3m–o).

**Extended Data Fig. 4 |:**
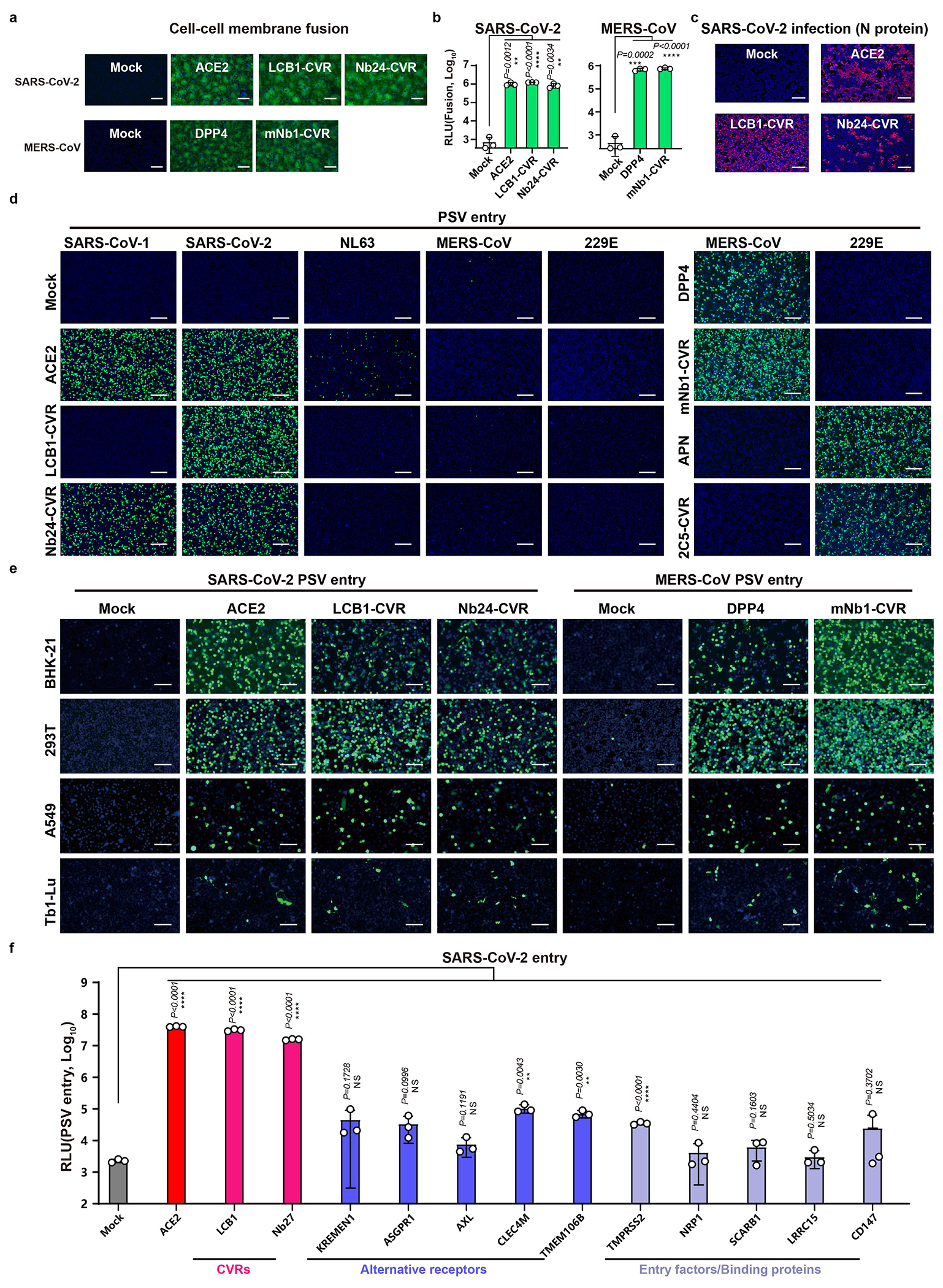
Comparing receptor function of CVRs with native receptors, or alternative receptors/coreceptors in different cell types. **a-c,** The ability of CVRs to promote cell-cell membrane fusion, and authentic SARS-CoV-2 infection is comparable to their native receptors. Spike and receptor-mediated cell-cell fusion was demonstrated by reconstituted GFP intensity (**a**) and relative light unit (RLU) of Renilla luciferase activity (**b**). Authentic SARS-CoV-2 infection was examined by immunostaining of intracellular N proteins at 24 hpi (**c**). Data are mean ± s.d. (biological triplicates) for **b**. Data are representative of two independent fusion assays or infection assays with similar results. **d,** Receptor specificity of different coronavirus PSVs in HEK293T stably expressing the native receptor or the indicated CVRs. **e,** SARS-CoV-2 and MERS-CoV PSV entry into various cell types expressing the indicated receptors. Data representative of two independent transfection and infection assays with similar results for **d** and **e**. **f,** SARS-CoV-2 PSV entry efficiencies in HEK293T cells expressing different receptors or entry factors. Data are presented as mean ± s.d. (n=3 independently infected cells.), representative of two independent transfection and infection experiments. **P<0.01, ***P<0.001, ****P<0.0001; NS, Not significant (*P*>0.05).

The functionality of CVRs compared with ACE2 was further demonstrated through a series of experiments showing membrane fusion, authentic SARS-CoV-2 infection, and virus specificity in different cell types (Extended Data Fig. 4a–e). The entry-promoting efficiencies of CVRs are significantly superior to several documented SARS-CoV-2 alternative receptors, coreceptors, entry factors, or binding proteins (Extended Data Fig.4f)^17^.

Together, we proposed a modular design strategy for generating CVRs comprising the customized VBD and modularized artificial receptor scaffolds (ARS) to support efficient coronavirus entry comparable to their native receptors (Fig. 1n).

### Mapping of functional CVR epitopes

**Extended Data Fig. 5 |:**
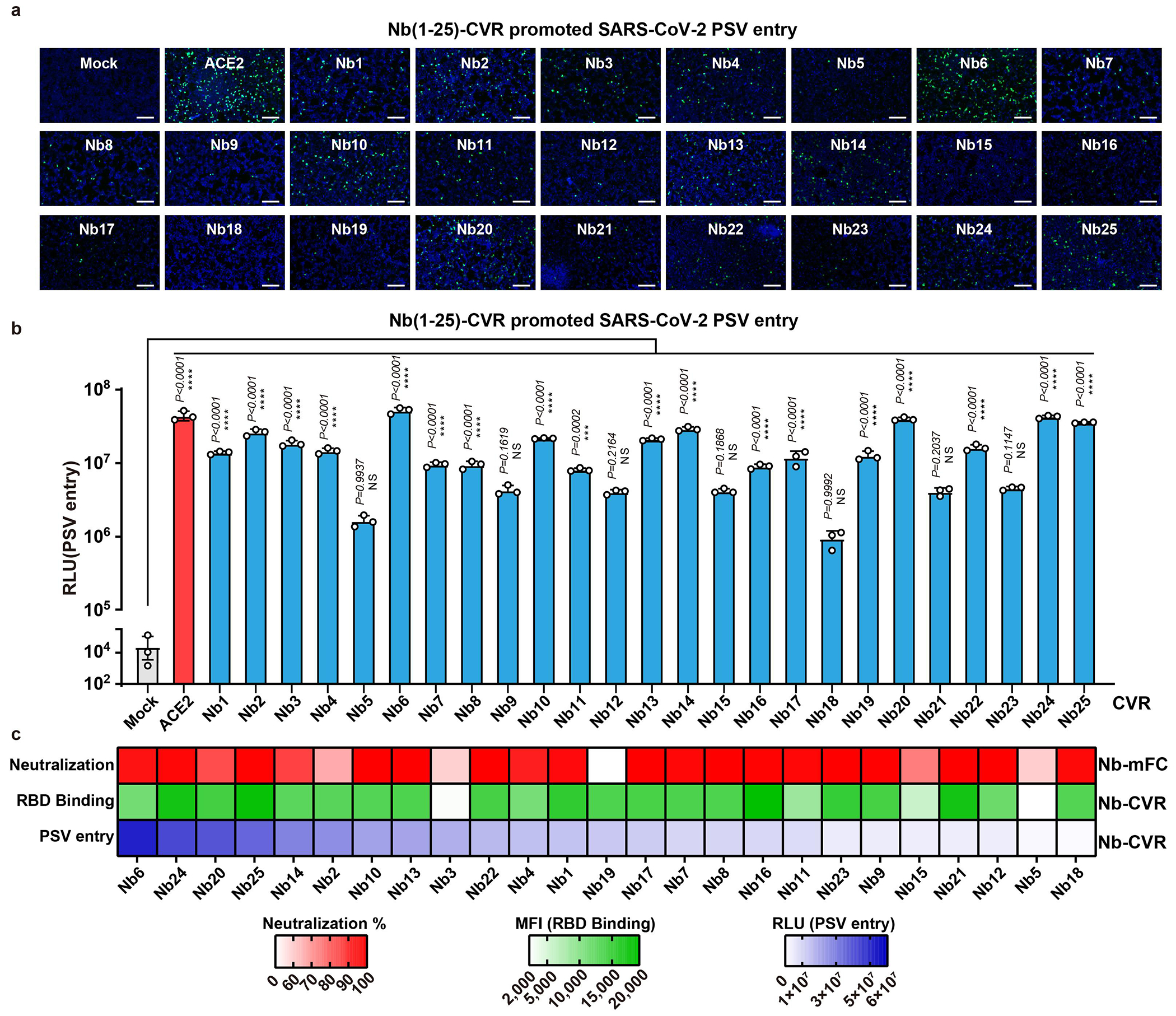
Relationship between the binding affinity, neutralizing activity, and CVR entry-promoting efficiency of 25 SARS-CoV-2 RBD-targeting nanobody-fused receptors. **a, b,** Assessment of the entry-promoting ability of 25 nanobody-CVRs in HEK293T cells, indicated by GFP (**a**) and the RLU (**b**), respectively. Data are presented as mean ± s.d. (n = 3 independently infected cells), representative of two independent experiments. One-way ANOVA analysis followed by Dunnett’s test. Scale bars: 200 μm. **c,** Comparing RBD binding, neutralization, and PSV entry-promoting ability of different nanobody-fused proteins in HEK293T cells. RBD-mFc binding and PSV entry assays were conducted in HEK293T transiently expressing the 25 nanobody-CVRs. The SARS-CoV-2 PSV neutralization assay was performed in HEK293T-ACE2 in the presence of indicated nanobody-Fc recombinant proteins (10 μg/mL). Data are representative results of two independent experiments with similar results and plotted by the mean (n = 3 independently infected/bound cells). ***P<0.001, ****P<0.0001; NS, Not significant (*P*>0.05).

In our initial exploration of the relationship between CVR receptor function and binding affinity or neutralizing activity, we evaluated 25 neutralizing nanobodies targeting the SARS-CoV-2 RBD^27^. However, we did not find a clear link between entry-supporting ability and binding affinity or neutralizing activity. These results suggest that there are other important factors at play, especially binding to epitopes that are not clearly defined for these 25 nanobodies (Extended Data Fig.5 and Supplementary Table 2).

**Extended Data Fig. 6 |:**
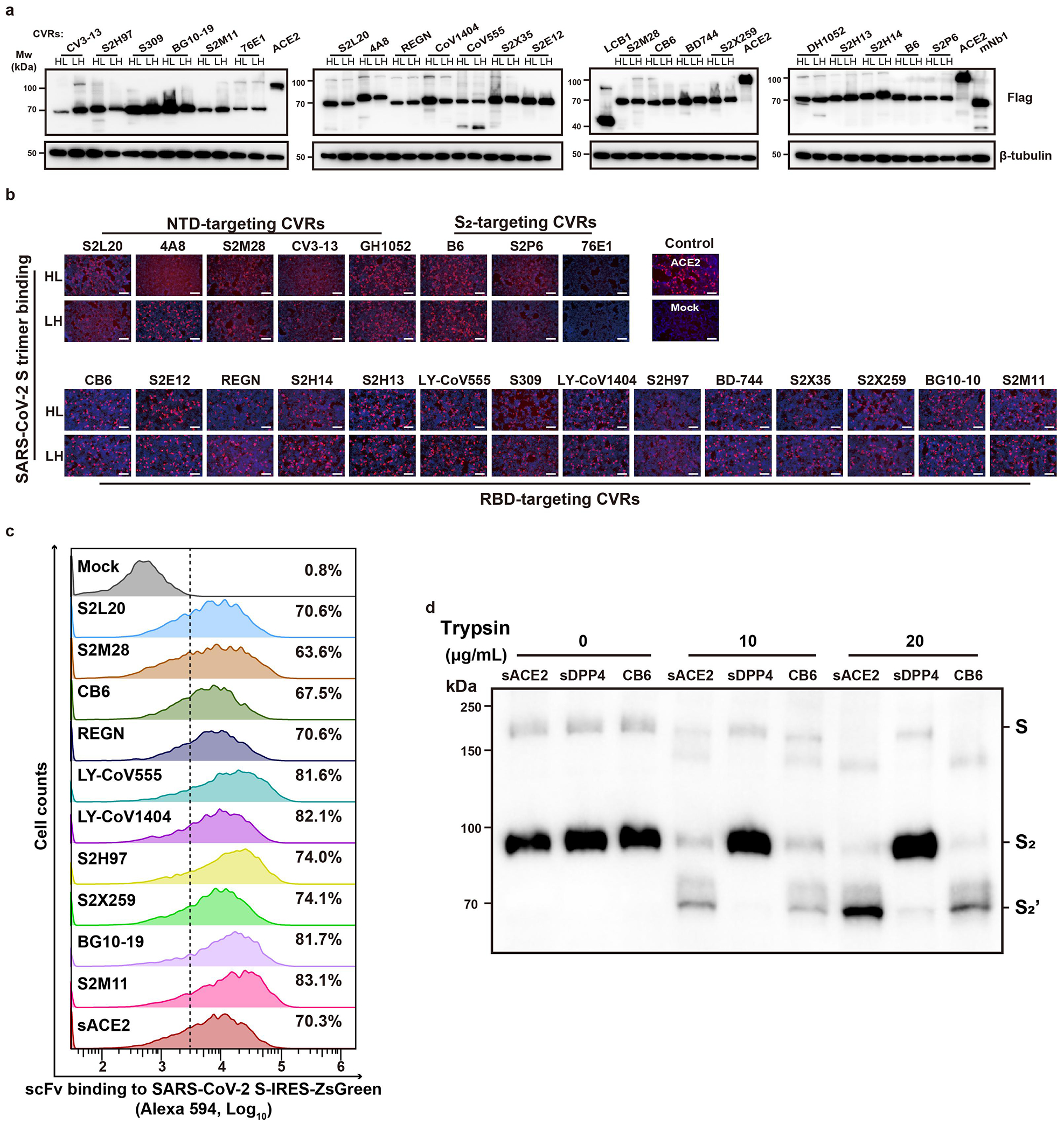
Expression and antigen-binding ability of CVRs targeting distinct SARS-CoV-2 neutralizing epitopes. **a,** Western blot analysis of the expression levels of indicated scFv-CVRs transiently expressed in HEK293T cells. Data are representative of two independent experiments. **b**, Binding of SARS-CoV-2 S-trimer to HEK293T cells expressing the indicated CVRs. Data are representative of three assays using independent preparations of proteins. **c**, Flow cytometry analysis of the binding efficiency of scFv-mFc with HEK293T cells transiently expressing the SARS-CoV-2 Spike proteins and ZsGreen simultaneously. The ZsGreen positive cells were gated for subsequent analysis of mFc binding efficiency. Data representative from a single experiment with mean values (n = 3 biologically independent cells) indicated. **d**, Trypsin-mediated S_2_’ cleavage of SARS2-CoV-2 PSV in the presence of soluble receptors or CB6-scFv-mFc. The concentrated SARS-CoV-2 PSV particles were incubated with 100 μg/mL of soluble receptors or CB6-scFv-mFc for 1 hour, followed by incubation with the indicated concentration of TPCK-treated trypsin for 30 minutes. Western blot analysis was conducted by detecting the S2P6 epitope in the S_2_ subunit. Data are representative of three independent assays with similar results.

**Fig. 2 |:**
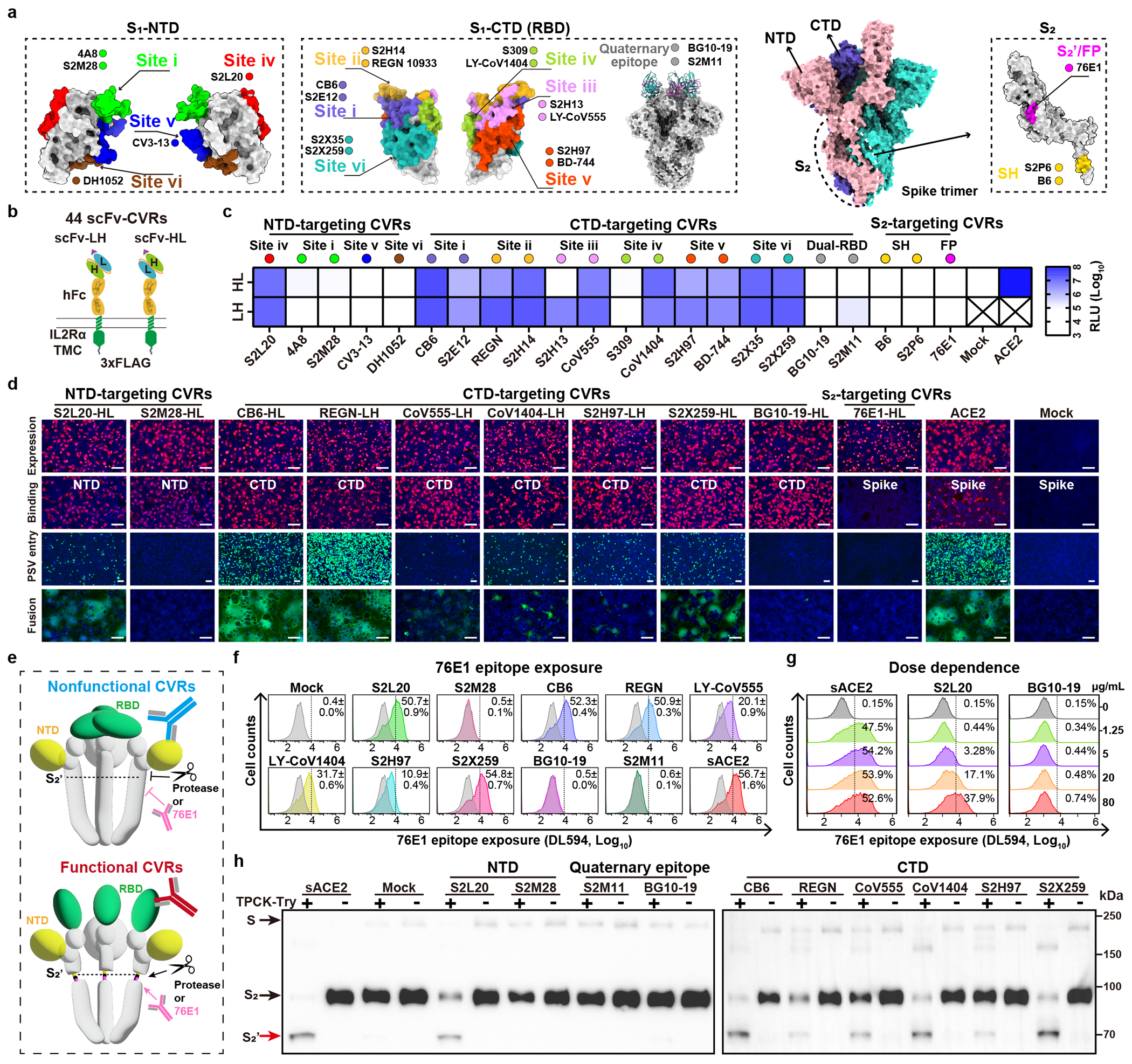
The impact of binding epitopes on receptor function and its molecular basis. **a,** Structural display of SARS-CoV-2 neutralizing epitopes in NTD, CTD, and S_2_ subunit, respectively. Various epitope types of 22 neutralizing antibodies are indicated based on a SARS-CoV-2 spike protein structure (6XR8). FP: fusion peptide. SH: stem helix. **b,** Schematic representation of 44 single-chain variable fragments (scFv)-CVRs with N-terminal light chain (LH) or heavy chain (HL), respectively. **c,** Heat map displaying SARS-CoV-2 PSV entry efficiency in HEK293T cells transiently expressing the indicated scFv-CVRs. Data are plotted by mean (n = 3 independently infected cells), representative of two independent experiments with similar results. SH: stem helix; FP: fusion peptide. **d,** Demonstration of CVR expression, antigen binding, PSV entry, and spike-mediated cell-cell fusion in HEK293T cells expressing indicated scFv-CVRs. Scale bars: 100 μm. Data presented were performed in two independent assays with similar results. **e,** Cartoon elucidates the functional receptor-mediated RBD conformational change and the subsequent exposure of 76E1 binding epitope and proteolytic cleavage at the S_2_’ cleavage sites. **f,** Flow cytometry analysis of 76E1 epitope exposure of SARS-CoV-2 S in the presence of indicated soluble scFv-mFc recombinant proteins. Data are presented as mean ± s.d. (n=3 biologically independent cells), representative of two independent experiments. **g,** Dose-dependent exposure of 76E1 epitope upon soluble hACE2 (sACE2) or S2L20 scFv-mFc coincubation, which was not detected in BG10-19 scFv-mFc. Data are representative of two independent experiments with similar results. **h,** Trypsin-mediated cleavage of S_2_’ site in SARS-CoV-2 pseudovirus particles in the presence of 100 μg/mL of indicated scFv-mFc. TPCK-try: 10 μg/mL. Blots representative of at least four independent cleavage assays with similar results. Dashed lines denote thresholds for positive ratio calculation for **f** and **g**.

Therefore, we engineered CVRs carrying scFvs derived from 22 well-characterized SARS-CoV-2 neutralizing antibodies covering a wide range of neutralizing epitopes (Fig. 2a)^28–35^. These antibodies were converted to scFv-based VBDs with N-terminal heavy chain (HL) or N-terminal light chain (LH), resulting in 44 CVRs (Fig. 2b). All CVRs were well-expressed and efficiently bound to the SARS-CoV-2 spike trimer, except for 76E1 which recognizes a conformationally masked epitope exposed after receptor binding (Extended Data Fig. 6a–b)^33,36^. The scFv-CVRs recognizing epitopes close to the canonical RBM (sites i, ii, and iii) supported efficient entry^37^, as was the case for many other RBD core domain-targeting CVRs (Fig. 2c). However, a subset of RBD epitopes were not suitable for CVR design, such as those recognized by S309 and two antibodies binding to a quaternary epitope spanning two neighboring RBDs and locking the spike in a closed conformation (BG10-19, S2M11)^28–30^. Unexpectedly, S2L20-CVR, which recognizes an NTD epitope (site iv), promoted robust entry, challenging the previous hypothesis that NTD-recognizing antibodies are insufficient to induce SARS-CoV-2 membrane fusion and entry without ACE2 (Fig. 2c)^22,32^. We further demonstrated the expression, antigen binding, pseudovirus entry, and membrane fusion supported by ten selected CVRs (Fig. 2d).

To elucidate the molecular basis of functional CVR design, we hypothesized that CVR function depends on their ability to induce proper conformational changes that lead to down-stream events required for membrane fusion, as previously proposed for the S230 SARS-CoV-1 mAb^38^, particularly RBD opening and exposure and cleavage of the S_2_’ cleavage site for fusion activation (Fig. 2e)^20,33^. Consistently, only scFv-mouse IgG Fc (scFv-mFc) corresponding to functional CVRs can induce the exposure of the 76E1 epitope in a dose-dependent manner (Fig. 2e–g, and Extended Data Fig. 6c), concurring with ACE2-induced exposure of the S_2_’ cleavage site and fusion peptide^36^. Furthermore, increased protease accessibility to and processing of the S_2_’ cleavage site induced by functional scFv-mFc concurs with the data from 76E1 epitope exposure assays (Fig. 2h and Extended Data Fig.6d).

### NTD-mediated entry by S2L20-CVR

**Extended Data Fig. 7 |:**
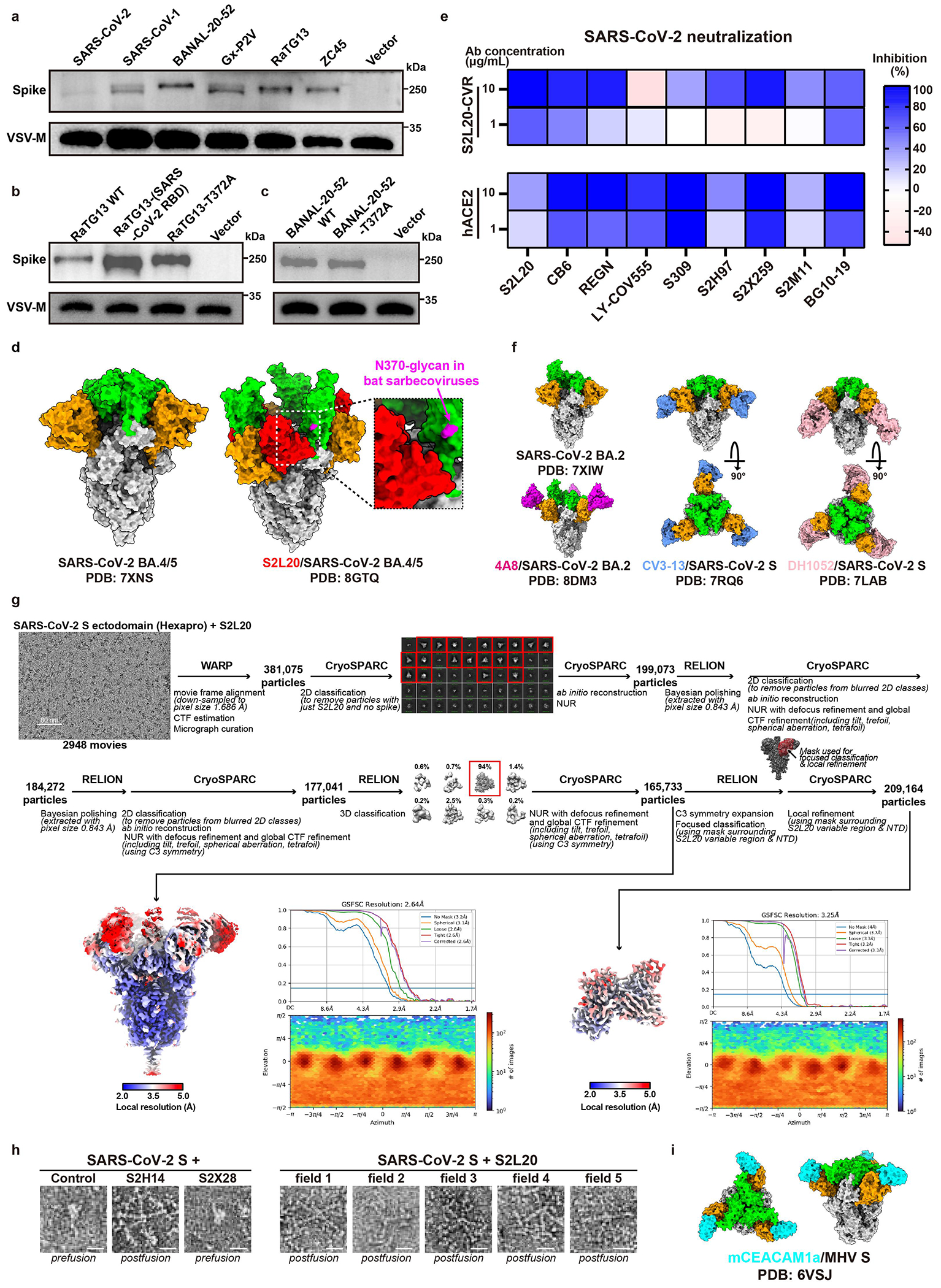
Molecular basis of NTD-mediated coronavirus entry. **a-c,** Package efficiency of PSVs carrying indicated sarbecoviruses spike glycoproteins (**a**) and the indicated mutants (**b, c**). Western blot was conducted by detecting the conserved S2P6 epitope. VSV-M serves as a loading control. Blots representative of two independent transfection assays for pseudovirus production. **d,** Structures of SARS-CoV-2 BA.4/5 spike trimer without antibody binding (left), or in complex with S2L20 (right). Dashed boxes highlighted the N370-glycan spatially proximate to the S2L20. **e,** Heatmap showing the inhibitory efficacy of indicated SARS-CoV-2 neutralizing antibodies against PSV entry in HEK293T-hACE2 or HEK293T-S2L20, with BSA as a control. Data are representative results of two independent neutralization assays and plotted by the mean (n=3 independently infected cells). **f,** Structures of SARS-CoV-2 BA.2 spike trimers with (upper) or without (lower) the binding of NTD-targeting 4A8, along with the side-view (top) and top-view (bottom) cryoEM structures of SARS-CoV-2 Wuhan-Hu-1 spike trimmers the binding of NTD-targeting CV3-13 and DH1052. Orange: NTD; Green: CTD; Red: S2L20; Magenta: 4A8; Blue: CV3-13; Pink: DH1052. **g,** CryoEM data processing workflow and validation of the S2L20-bound SARS-CoV-2 S CryoEM structure. **h,** Negative stain microscopy of prefusion SARS-CoV-2 S-glycoprotein (without stabilizing proline substitutions) incubated without antibody, or with S2H14, S2X28, or S2L20 as indicated. S2H14 is known to promote the transition to the postfusion state and was used as a control^37^. Scale bars: 10 nm. Data representative of images captured from two independent experiments with similar results. **i**, Top-view and side-view CryoEM structures depicting soluble mCEACAM1a (cyan) in complex with MHV spike trimer (gray). NTD and CTD of MHV S are indicated in orange and green, respectively.

**Fig. 3 |:**
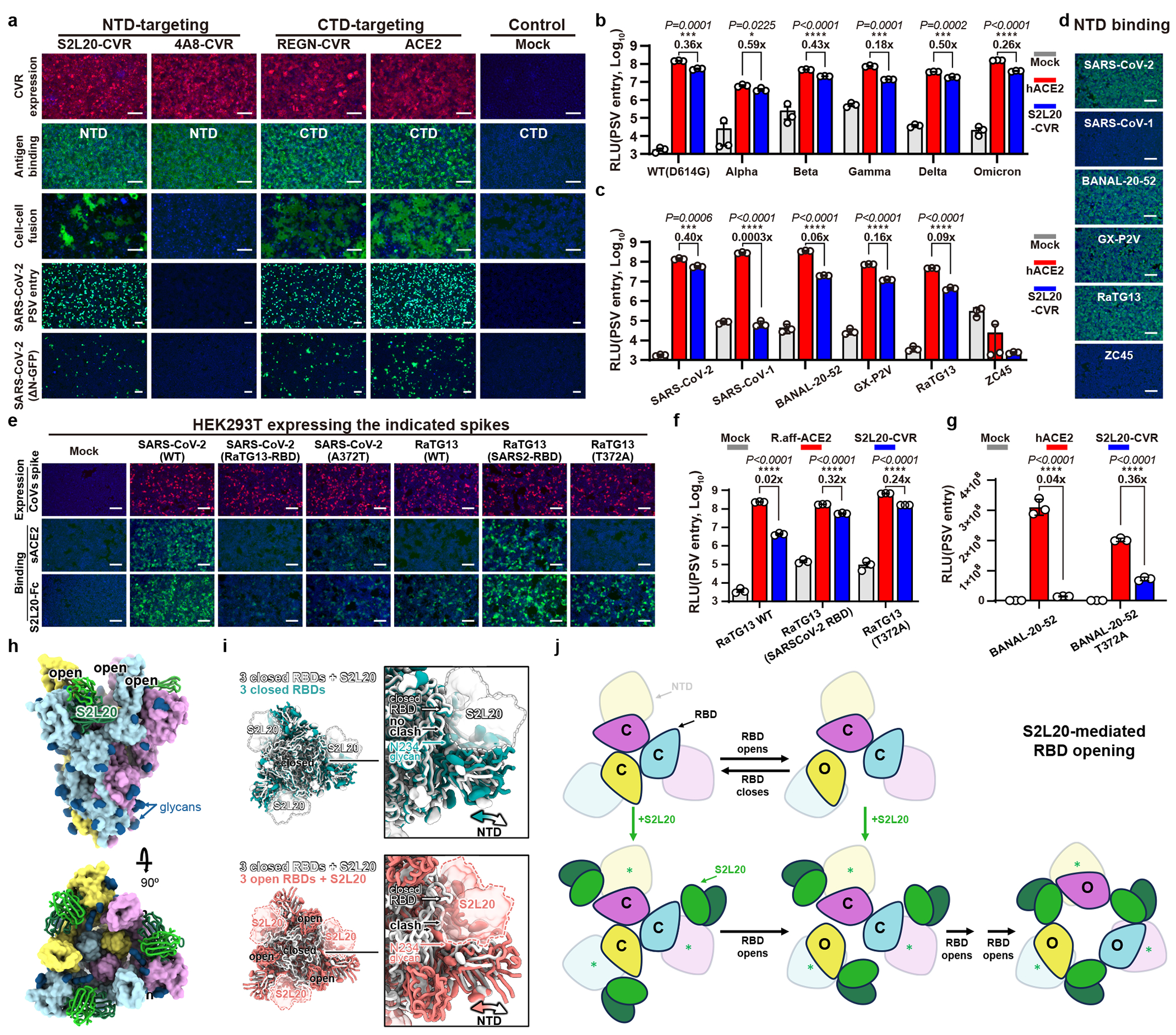
NTD-mediated sarbecovirus entry promoted by S2L20-CVR. **a,** CVR expression, SARS-CoV-2 NTD/CTD-mFc binding, cell-cell fusion, PSV entry, and SARS-CoV-2(ΔN-GFP) infection in HEK293T stably expressing indicated CVRs. Data representative of two independent transfection and functional assays. **b, c,** PSV entry of SARS-CoV-2 VOCs (**b**) or various sarbecoviruses (**c**) in HEK293T stably expressing hACE2 or S2L20-CVR. **d,** Sarbecoviruses NTD-mFc binding efficiencies in HEK293T-S2L20-CVR. Data representative of three independent assays. **e,** S expression levels and corresponding sACE2 or S2L20-mFc binding efficiencies. Data representative of two independent binding assays. **f, g,** Impact of T372A mutation on S2L20-CVR-supported PSV entry of RaTG13 (**f**) or BANNAL-20-52 (**g**). R.aff: *R.affinis* allele 9479. **h**, SARS-CoV-2 Wuhan-Hu-1 Hexapro S ectodomain trimer bound to the S2L20 Fab. S protomers: pink, cyan, and gold; N-linked glycans: dark blue; S2L20 Fab variable heavy and light chains: dark and light green, respectively. **i**, (Top) Superimposition (based on the S_2_ subunit) of the structures of S2L20-bound SARS-CoV-2 S with closed RBDs (white, 7N8H) and of S with closed RBDs (teal, PDB 7K43). (Bottom) Superimposition (based on the S_2_ subunit) of the structures of S2L20-bound SARS-CoV-2 S with closed RBDs (white, 7N8H) and of S2L20-bound SARS-CoV-2 S with open RBDs (this study). Insets: zoomed-in views of the interface between the NTD, RBD, and S2L20. S2L20 is shown as a semi-transparent surface. The S2M11 Fab is not shown for clarity in both panels. (**j**) Proposed mechanism of S2L20-mediated RBD opening and stabilization. C: closed; O: open. *: NTD repositioning. For **b**, **c**, **f**, and **g**, data are mean ± s.d. (n = 3 independently infected cells) out of two independent experiments. Ratios indicate PSV entry supported by S2L20-CVR compared to that supported by ACE2. Unpaired two-tailed Student’s *t*-tests. Scale bars: 100 μm. *P<0.05, ***P<0.001, ****P<0.0001.

We next sought to characterize the NTD-mediated entry promoted by S2L20-CVR. We confirmed that S2L20-CVR acts as a functional receptor for SARS-CoV-2, supporting membrane fusion, pseudovirus entry, and authentic virus infection (Fig. 3a). Additionally, S2L20-CVR effectively facilitated pseudovirus entry of the five SARS-CoV-2 variants of concern (VOCs) tested and of three other sarbecoviruses (BANAL-20-52, RaTG13, and GX_P2V) (Fig. 3b–d and Extended Data Fig.7a). SARS-CoV-1 and ZC45 cannot use S2L20-CVR for entry due to lack of binding^32^ (Fig. 3c,d).

Despite showing similar NTD-binding efficiency, S2L20 showed relatively lower efficiency in supporting RaTG13 and BANAL-20-52 entry than SARS-CoV-2 (Fig. 3c,d). Since the lack of an N370 glycan has been reported as a distinct feature of SARS-CoV-2, we generated several mutants to investigate the impact of CTD sequences and of the N370 glycan on S2L20-CVR promoted entry^39^. A SARS-CoV-2 spike harboring either the RaTG13 RBD or an A372T (glycan knock-in) substitution bound more weakly to the soluble forms of hACE2 (sACE2) or S2L20-mFc than SARS-CoV-2 RBD-harboring spikes. We note that RaTG13 has a lower affinity for hACE2 than for an ACE2 orthologue from its host species (*Rhinolophus. affinis*) (Fig. 3e). We hypothesized that the N370 glycan might sterically interfere with S2L20 binding due to its proximity to the NTD-bound S2L20 Fab fragment. Consistently, the T372A mutation in the RaTG13 or BANAL-20-52 spike, abolishing the N370 glycosylation, significantly enhanced S2L20-CVR mediated entry (Fig. 3f–g and Extended Data Fig. 7b–d)^40^.

We next investigated whether SARS-CoV-2 CTD-targeting neutralizing antibodies could interfere with NTD-mediated entry in cells expressing S2L20-CVR compared to hACE2-expressing cells. As expected, S2L20 exhibited higher neutralizing activity in S2L20-CVR-expressing cells. Importantly, although several antibodies (LY-CoV555, S309, and S2X259) showed reduced neutralizing efficiency in S2L20-CVR expressing cells, some CTD-binding antibodies exhibited similar neutralizing activity in both models (Extended Data Fig. 7e). These data suggest an association between RBD and S2L20-CVR-mediated entry.

A prior study described the CryoEM structure of S2L20 in complex with SARS-CoV-2 BA.5 S in which the three RBDs are in the “up” conformation, whereas the apo BA.5 S structure exhibits the three RBDs in the “down” conformation^41,42^. In contrast, other NTD-targeting antibodies, including 4A8, CV3-13, and DH1052, do not appear to induce the RBD “up” conformation (Extended Data Fig. 7f) ^43–45^. RBD opening can also be elicited by binding of ACE2, RBM-targeting antibodies and minibinders, or of any antibodies targeting an RBD epitope that is occluded in the closed state^24,38,46–49^. Therefore, we hypothesized that S2L20-CVR induces RBD opening thereby promoting viral entry, similar to ACE2,. To gain further insights into how this might occur, we determined a CryoEM structure of the S2L20 Fab bound to SARS-CoV-2 Wuhan-Hu-1 S ectodomain with stabilizing HexaPro mutations (Fig. 3h, Extended Data Fig. 7g and Extended Data Table 1). Our structure features one S2L20 Fab bound to each of the three spike NTDs and three open RBDs instead of the previously described mixture of up and down RBD states for the apo spike (Fig. 3h)^50^. However, we previously found that S2L20 can bind to the spike ectodomain when all the RBDs are locked closed due to the presence of the S2M11 Fab that recognizes a quaternary epitope spanning two neighboring RBDs (Fig.3i). Relative to closed S2L20-bound spike structures^51,52^, the open S2L20-bound spike structure exhibits a repositioning of the NTDs and bound S2L20 along with stabilization of a conformation of the N234 glycan that would be sterically incompatible with subsequent RBD closing (Fig.3i). Overall, these data support a molecular ratchet mechanism for S2L20-mediated RBD-opening, whereby (i) S2L20 can bind NTDs adjacent to closed RBDs, and when an RBD opens, the NTD and S2L20 shift to a position that blocks subsequent RBD closing or (ii) S2L20 could bind to NTDs adjacent to already open RBDs, thereby preventing them from adopting the closed state. Consistent with this hypothesis, incubation of purified spike ectodomain (without stabilizing proline substitutions) with S2L20 triggered spike fusogenic conformational changes yielding postfusion trimers (Fig.3j, and Extended Data Fig. 7h).

Although the induction of three-RBD “up” conformation upon S2L20 binding appears to be crucial for S2L20-CVR receptor functionality, CEACAM1a is an entry receptor for MHV despite recognizing an NTD surface proximal to that targeted by the 4A8 SARS-CoV-2 antibody, the latter binder does not alter RBD opening nor support viral entry (Extended Data Fig. 7i)^5,43,53^. This suggests that different coronaviruses might adopt distinct receptor recognition mechanisms to achieve NTD-mediated entry or that yet undiscovered triggering mechanisms lead to MHV RBD opening and viral entry, as recently discovered for HKU1^12,54,55^.

### CVR generalizability among coronaviruses

**Extended Data Fig. 8 |:**
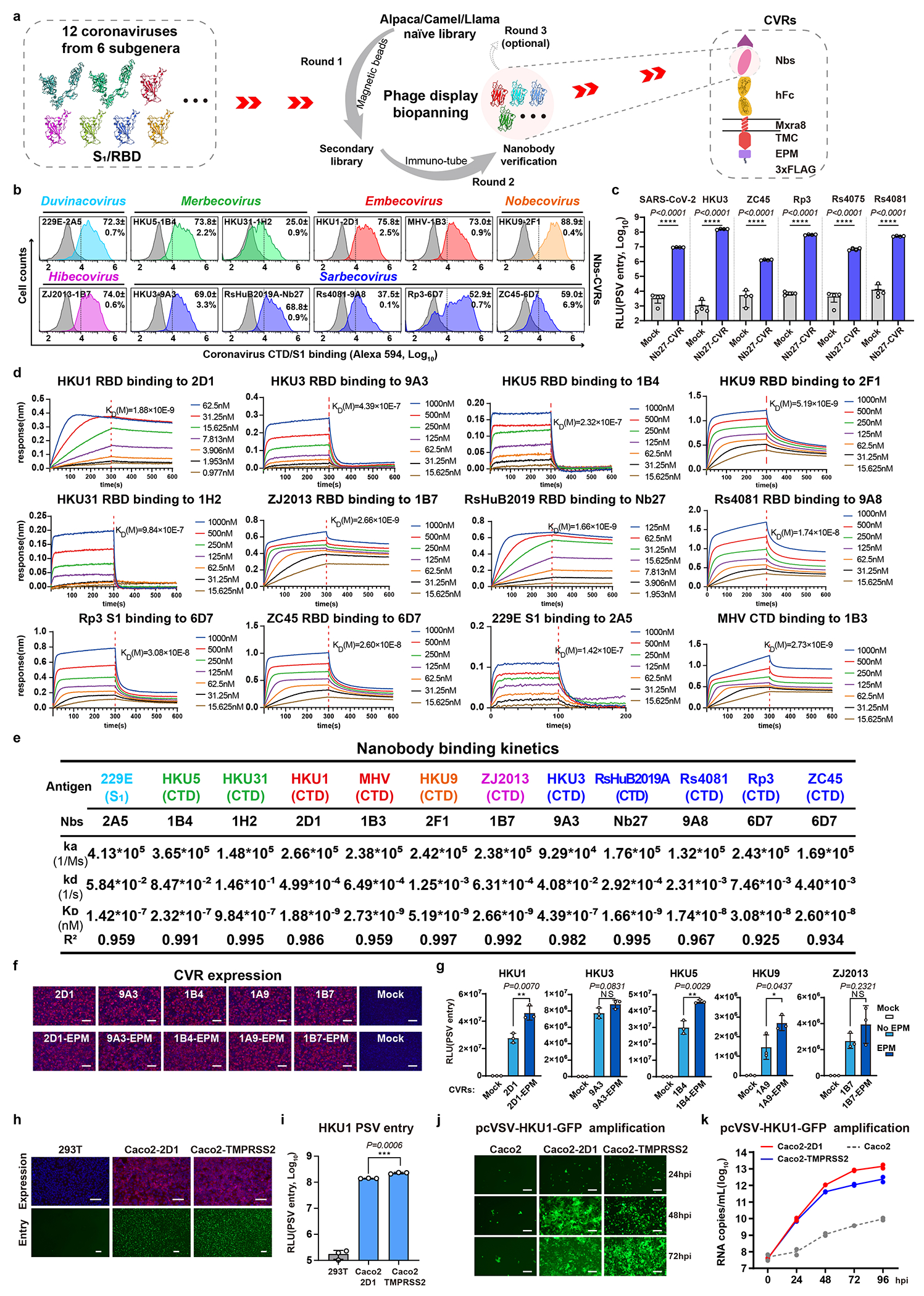
Generation and characterization of VBDs used for CVRs customized for various coronaviruses. **a**, Workflow demonstrating the customization of nanobody-based CVRs for specific coronaviruses. **b**, Coronavirus CTD or S_1_ binding in HEK293T cells transiently expressing the corresponding CVRs. Dashed lines indicate thresholds for positive ratio calculation. Data are presented as mean ± s.d. (n=3 biologically independent cells), representative of two independent experiments. **c,** The pan-sarbecovirus entry-promoting ability of CVR-Nb27 was evaluated by six different sarbecoviruses in 293T cells. Data are presented as mean ± s.d. (biological quadruples of infected cells), representative of two independent infection assays. Unpaired two-tailed Student’s *t*-tests. **d**, BLI analyses of binding kinetics of immobilized nanobody-Fc fusion proteins with the soluble RBD and S_1_-hFc (for 229E) of the indicated coronaviruses. **e,** a summary of binding kinetics of nanobodies bound to the immobilized virus antigens. **f, g,** Expression (**f**) and entry-supporting efficiency (**g**) of the CVRs with or without EPM transiently expressed in the HEK293T cells. EPM: endocytosis prevention motif. Data are mean ± s.d. (n = 3 independently infected cells). Unpaired two-tailed Student’s *t*-tests. Experiments were performed twice with similar results, and representative data were shown. **h-k,** HKU1 specific 2D1-CVR exhibited a comparable receptor function to TMPRSS2. The expression (**h**) and HKU1-PSV entry promoting efficiency (**h**, **i**) of the two receptors were examined. The amplification of propagation-competent rVSV-HKU1-GFP in Caco2 cells expressing 2D1-CVR and TMPRSS2 was demonstrated by GFP (**j**) and RNA accumulation (**k**). Scale bars: 100 μm for **f**, **h**, and **j**. Data are mean ± s.d. (biologically triplicates of infected cells) analyzed by unpaired two-tailed Student’s *t*-tests for **i**. Data presented are RNA copies of two independently infected cells with each point representing the mean of technical duplicates (RT-qPCR) for **k**. Experiments presented were independently performed twice with similar results for **h**-**j** and single time for **k**. *P<0.05, **P<0.01, ***P<0.001, ****P<0.0001; NS, Not significant (*P*>0.05).

**Fig. 4 |:**
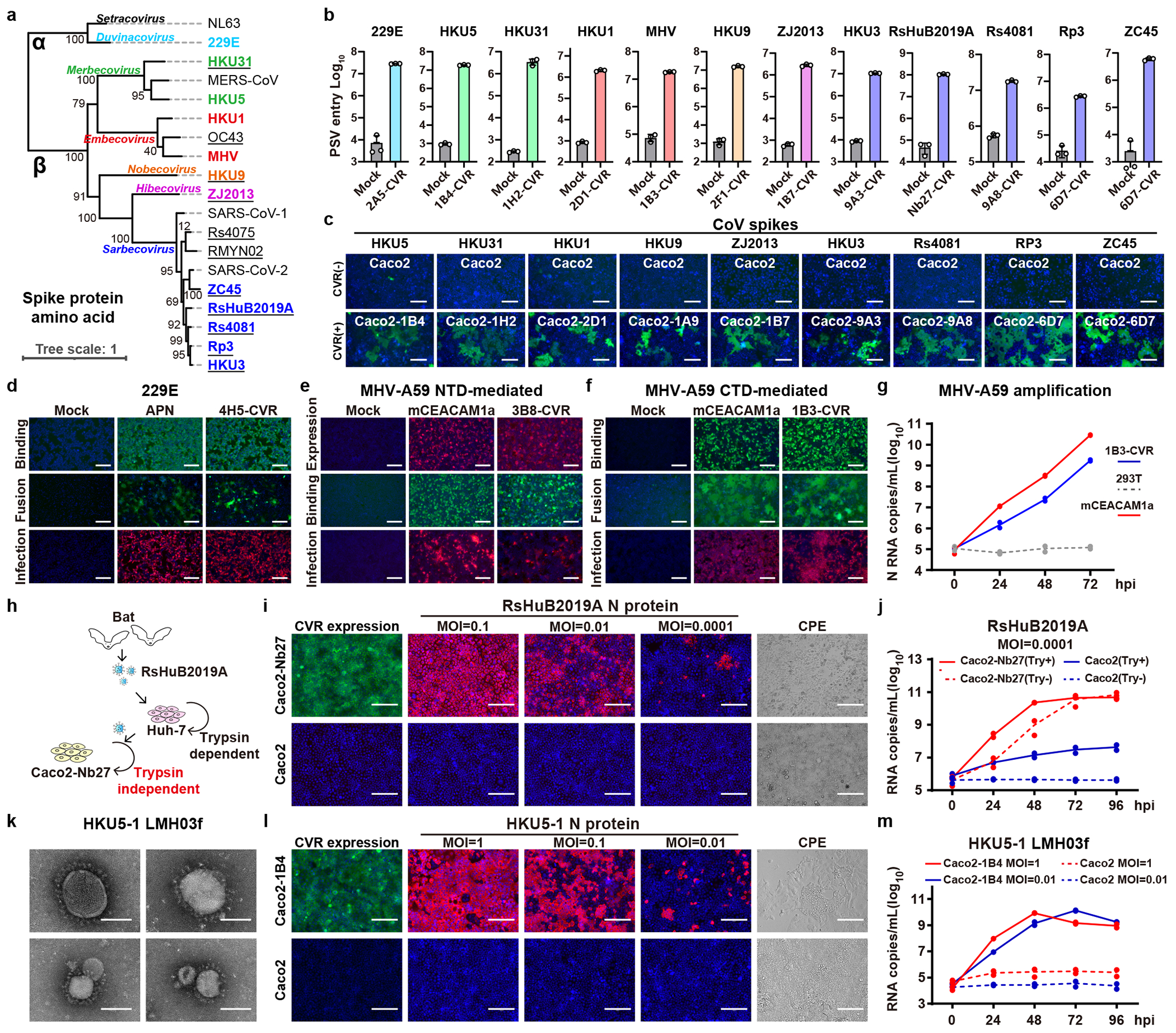
CVRs supported entry and amplification of various coronaviruses. **a,** Phylogenetic tree based on coronavirus S amino acid sequences. Underline: without known receptors. **b,** PSV entry of twelve coronaviruses in HEK293T transiently expressing indicated CVRs. Data are mean ± s.d. (biological triplicates), representative of three independent infection assays. **c,** Cell-cell fusion promoted by indicated coronavirus S in Caco2 cells with or without stable expression of corresponding CVRs with trypsin treatment. Data representative of three independent experiments. **d,** 229E-S_1_-mFc binding, cell-cell fusion, and authentic 229E infection in HEK293T stably expressing APN or 4H5-CVR. **e-f,** MHV-A59 antigen binding, cell-cell fusion, and authentic MHV infection in HEK293T stably expressing mCEACAM1a, NTD-targeting (**e**), or CTD-targeting (**f**) CVRs. **g**, MHV-A59 RNA copies in supernatant from infected cells expressing mCEACAM1a or 1B3-CVR. **h,** Cartoon illustrating different trypsin dependence of RsHuB2019A propagation in Huh-7 and Caco2-Nb27 cells. **i**, CVR expression, N protein, and CPE in cells inoculated with RsHuB2019A at indicated MOI (no trypsin). **j**, Accumulation of viral RNA in supernatant of indicated cells infected with RsHuB2019A. Try: 100 μg/mL Trypsin in DMEM+2%FBS. **k,** Transmission electron microscopy analysis of HKU5-1 virions. Representative images of a single experiment are shown. **l,** CVR expression, N protein, and CPE in indicated cells inoculated with HKU5-1 at indicated MOI. **m,** Accumulation of HKU5 RNA in supernatant of cells inoculated with HKU5-1 at indicated MOI. Infection was examined by S immunofluorescence for **d**, **e**, and **f** and N protein immunofluorescence for **i** and **l** at 24 hours post-infection (hpi). For **g, j, and m**, data presented are RNA copies of two independently infected cells with each point representing the mean of technical duplicates (qRT-PCR). For **d**-**g**, **i**-**j**, and **l**-**m,** data presented are from at least two independent experiments with similar results for each virus, respectively. Scale bars for c-f, 100 μm; for k, 60 nm; for i,l, 125 μm.

We extended our approach to generating CVRs for 12 coronaviruses from six subgenera, most of them without known receptors (Fig. 4a and Supplementary Table 1)^1^. To develop effective VBDs, we used magnetic beads and immunotube-assisted phage display biopanning to screen for coronavirus spike-specific nanobodies from naïve libraries (Extended Data Fig. 8a). Lead CVR nanobodies were identified through validated binding to the RBD or S_1_ subunit (Extended Data Fig. 8b and Supplementary Table 3). We also included a broadly-neutralizing nanobody Nb27, which cross-reacts with RsHuB2019A and several other sarbecoviruses (Extended Data Fig. 8c)^56^. We determined the binding kinetics of the selected nanobodies to the 12 coronavirus antigens through biolayer interferometry (BLI) assays (Extended Data Fig. 8d,e). Efficient S-mediated pseudovirus entry and cell-cell membrane fusion were demonstrated in cells stably expressing the indicated CVRs, achieving approximately 10^2^ to 10^4^-fold increase in pseudovirus entry compared with mock controls (Fig. 4b,c). The CVRs carrying EPM exhibited better cell surface localization and promoted even more efficient entry (Extended Data Fig. 8f,g). The HKU1-specific 2D1-CVR exhibited a comparably effective receptor function to TMPRSS2 (Extended Data Fig.8h–k). Moreover, several CVRs designed for 229E and MHV-A59 supported efficient membrane fusion and authentic virus propagation, including the 1B3-CVR targeting the MHV-CTD (Fig. 4d–g).

**Extended Data Fig. 9 |:**
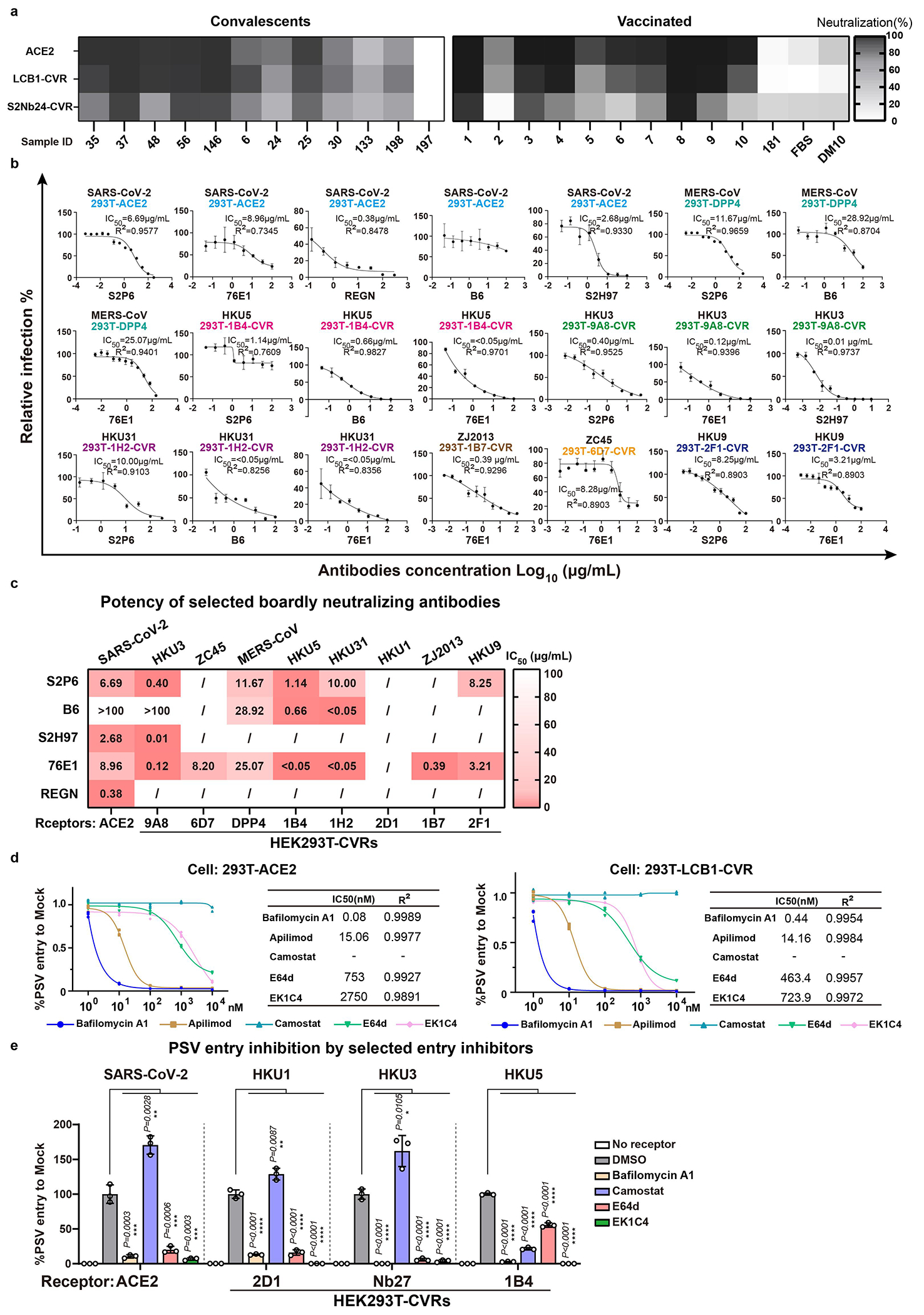
Neutralization and inhibition assays based on CVR-expressing HEK293T cells. **a**, Comparison of neutralization profiles of sera collected from COVID-19 convalescents (left) or vaccinated individuals (right) based on HEK293T cells expressing ACE2 or two different CVRs. Serum dilution: 1:200. Heatmap plotted by the mean values (biologically triplicates of infected cells), which are representative results out of two independent experiments. **b**, **c,** Neutralization assays of several broadly neutralizing antibodies against PSV entry of representative coronaviruses in HEK293T stably expressing the indicated CVRs. Neutralization curves (**b**) and a summary of IC_50_ (**c**) against each virus are shown. The RBD-targeting REGN 19033 (REGN) was employed as a control. /: no inhibition detected. Data are mean ± s.d. (biologically triplicates of infected cells). **d,** The IC_50_ of selected entry inhibitors against SARS-CoV-2 PSV entry was determined in both HEK293T-ACE2 or HEK293T-LCB1-CVR cells. Data are mean ± s.d. (biologically triplicates of infected cells). **e**, Inhibitory efficacy of inhibitors against PSV entry of SARS-CoV-2-D614G, HKU1, HKU3 and HKU5 in HEK293T cells stably expressing the indicated CVRs. Data are mean ± s.d. (biologically triplicates of infected cells), representative of two infection inhibition assays with similar results. *P<0.05, **P<0.01, ***P<0.001, ****P<0.0001; NS, Not significant (*P*>0.05).

We next evaluated the CVR-based infection models for neutralizing antibody assessment. We compared the neutralizing activity of sera, collected from COVID-19 convalescents or vaccinated individuals, in HEK293T cells expressing ACE2, LCB1-CVR, and Nb24-CVR^27^, respectively. We observed comparable overall serum inhibitory profiles testing the three receptors side-by-side, although slight differences were noted with Nb24-CVR, recognizing an RBD epitope distant from the RBM (Extended Data Fig.9a), possibly due to modulation of apparent neutralization potencies resulting from distinct receptor usage or expression levels. This indicates the utility of the CVR-based system for evaluating the effectiveness of humoral immunity, ideally with CVRs recognizing the RBM region. We evaluated the cross-reactivity of several broadly neutralizing β-CoV antibodies against a panel of coronaviruses and found that 76E1 exhibits the greatest neutralization breadth, consistent with the epitope conservation (Extended Data Fig.9b,c). Furthermore, we investigated the potential of the CVR-based infection system for evaluating antivirals targeting different entry steps, including proteolytic cleavage, endosome acidification, and membrane fusion^10,57–59^. Comparable inhibitory efficacy was observed when comparing spike-mediated entry via ACE2 and LCB1-CVR (Extended Data Fig.9d). We further tested these inhibitors against SARS-CoV-2, HKU1, HKU3, and HKU5 entry into HEK293T cells mediated by the corresponding CVRs. The overall efficiencies of the entry inhibitory tested were similar among the four viruses except for HKU5 (HKU5-1, LMH03f isolate) which exhibited a higher sensitivity to the TMPRSS2 inhibitor camostat rather than the cathepsin inhibitor E64d (Extended Data Fig.9e). The fusion inhibitor EK1C4^57^, the optimized form of OC43-HR2 lipopeptide, exhibited a broad-spectrum inhibitory effect (Extended Data Fig.9e).

**Extended Data Fig. 10 |:**
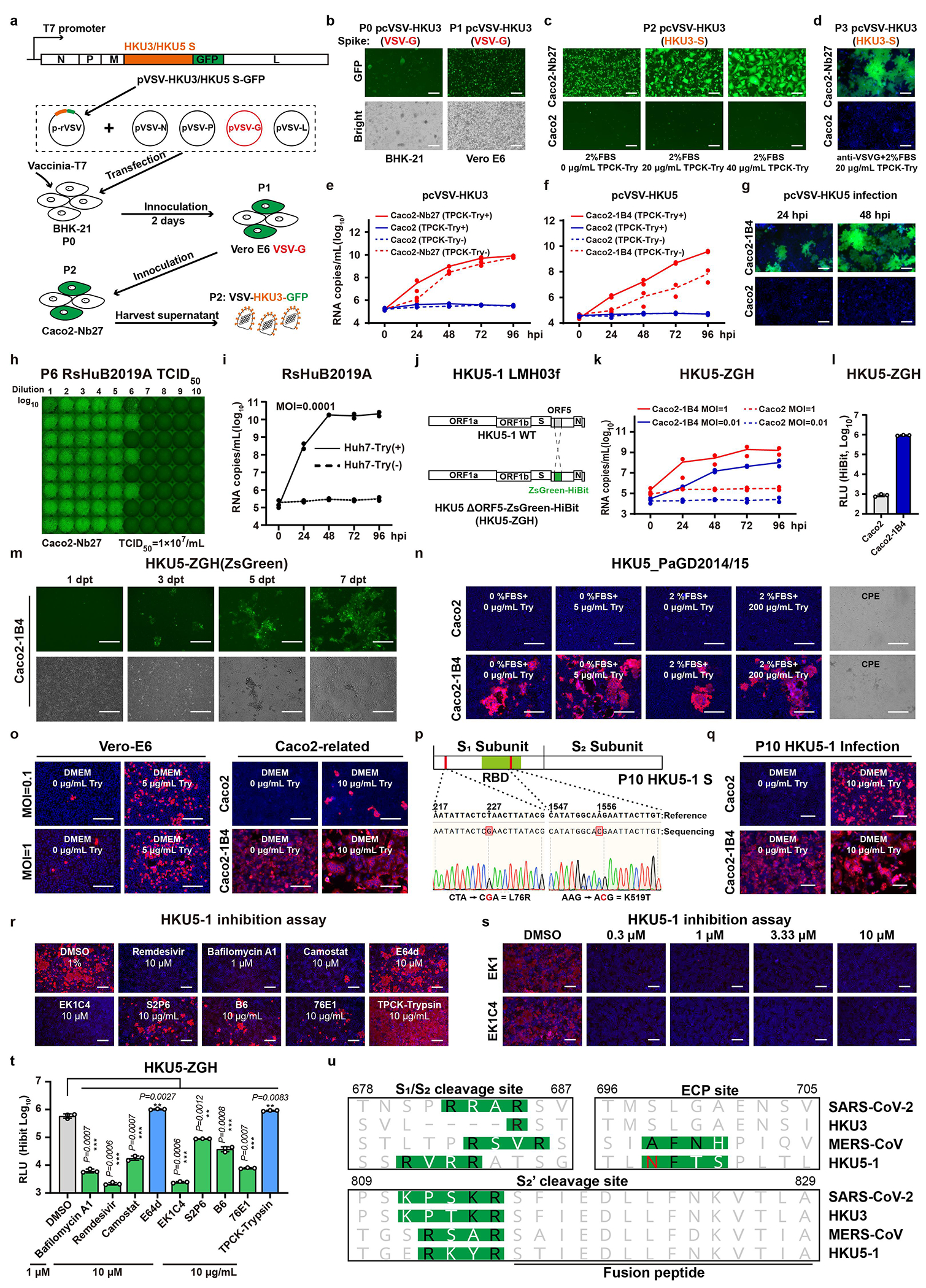
Characterization of CVR-promoted amplification of replication-competent pcVSV-CoVs or authentic coronaviruses. **a,** Genetic organizations and workflow for generating replicable pcVSV-HKU3 or pcVSV-HKU5. **b,** Successful rescue (P0) and amplification (P1) of pcVSV-HKU3 assisted by VSV-G. Representative images of an experiment that was conducted for a single time. **c**, **d**, Trypsin-enhanced cell-cell fusion (**c**) and VSV-G-independence (**d**) of pcVSV-HKU3 infection in Caco2-Nb27 (MOI: 0.001). Data are representative of four independent infection assays with similar results. **e**, **f**, Accumulation of pcVSV-HKU3 (**e**) or pcVSV-HKU5 (**f**) RNA in the supernatant at indicated time points. **g,** pcVSV-HKU5 mediated cell-cell fusion in Caco2 or Caco2-1B4 cells at MOI=0.1. TPCK-Try+: 20 μg/mL TPCK-treated Trypsin in DMEM+2% FBS. Representative of two independent experiments. **h**, TCID_50_ determination assay for RsHuB2019A in Caco2-Nb27 cells by the Red-Muench method. Caco2-Nb27 cells were inoculated with a 10-fold serial dilution of RsHuB2019A containing supernatant (Passage 6). The TCID_50_ was determined using immunofluorescence to detect the presence of N protein expression of the inoculated cells at 4 dpi. **i**, Trypsin-dependent amplification of RsHuB2019A in Huh-7 cells. The RsHuB2019A genomic RNA copies in the supernatant collected at indicated time points of infected Huh-7 cells were quantified by RT-qPCR using RdRp-specific primers. Inoculation was conducted at an MOI of 0.0001, with or without trypsin treatment. Try: 100 μg/mL Trypsin in DMEM. **j,** Genetic organizations of the HKU5 (HKU5-1 LMH03f) ΔORF5-ZsGreen-HiBit (HKU5-ZGH). **k-m**, Supernatant RNA copies of HKU5-ZGH (**k**) in Caco2-1B4 cells, ZsGreen-HitBit signal (**l**), and increase in ZsGreen intensity (P0) (**m**). Representative of two independent infection assays are shown. Data are mean ± s.d. (n = 3 independently infected cells) for **l**. **n**, Isolation of HKU5 (strain PaGD2014/15) from bat samples by Caco2-1B4 cells and its trypsin-dependent propagation. **o**, Vero E6 and Caco2 cells were infected with HKU5-1 (LMH03f) with or without trypsin treatment. Data are representative of three independent experiments. **p**, Sequencing results show L76R and K519T mutations in HKU5-1 spikes after ten passages in Caco2-1B4 cells. **q**, HKU5-1 after ten passages carrying mutations remains unable to infect Caco2 cells without exogenous trypsin treatment. Caco2-1B4 with CVR expression was included as a positive control. **r**, Efficacy of indicated antiviral reagents against HKU5-1 infection in Caco2-1B4 cells assessed by intracellular N proteins at 48 hpi. **s**, Efficient inhibition of HKU5 entry by EK1 and EK1C4 peptides in Caco2-1B4. **t**, Inhibitory effect of selected anti-viral reagents against authentic HKU5-ZGH infection in Caco2-1B4. Inhibitors were coincubated with either the cells or the viruses for 1h and present in the culture medium during infection. The HiBit-based luciferase activity was determined at 48 hpi to assess the inhibitory effect of selected anti-viral reagents against the infection of authentic HKU5-ZGH in Caco2-1B4. Data are representative of two independent infection assays for **r**-**t**. **u**, Overview of the protease cleavage sites of selected coronaviruses. The residue responsible for reduced endosomal cysteine protease activity (ECP) is marked in red, numbering based on SARS-CoV-2. The HKU5 infection efficiencies in **n**, **o**, **q**, **r**, and **s** were assessed using rabbit polyclonal antibodies targeting the HKU5 N protein (Cy3) at 48 hpi. Data presented are RNA copies of two independently infected cells with each point representing the mean of technical duplicates (RT-qPCR) for **e**, **f**, **i,** and **k**, representative of two independent experiments. Scale bars: 125 μm for all images.

To evaluate CVR-supported multiple-round virus propagation, we created propagation-competent (pc) VSV recombinant viruses by replacing the VSV-G gene with HKU3 or HKU5 S genes, and additionally incorporating a GFP-expressing cassette for visualization (Extended Data Fig.10a). The pcVSV-HKU3 and pcVSV-HKU5 were successfully rescued with the aid of VSV-G provided *in trans* (Extended Data Fig.10b). Efficient propagation of pcVSV-HKU3 and pcVSV-HKU5 was observed in CVR-expressing cells in a S-dependent manner, as evidenced by syncytia formation with green fluorescence and viral RNA accumulation, which was further enhanced by exogenous trypsin (Extended Data Fig.10c–g). Subsequently, we investigated whether CVRs can promote the propagation of authentic coronaviruses that are difficult to culture. RsHuB2019A is an ACE2-independent bat sarbecoviruses recently isolated from field samples^3^. Isolation and propagation of this virus were used to be carried out in Huh-7 under a serum-free culture condition with exogenous trypsin, with viral infection being difficult to detect while maintaining normal cell morphology (Fig. 4h). Our results demonstrate that Caco2-Nb27 efficiently supported RsHuB2019A propagation, even at very low multiplicity of infection (MOI) and in trypsin-free culture medium supplemented with 2% FBS, enabling observation of cytopathic effect (CPE) (Fig. 4i,j and Extended Data Fig. 10h,i).

Furthermore, we explored the feasibility of rescuing or isolating authentic HKU5 through CVR-expressing cells. Utilizing a coronavirus reverse genetics approach, we generated a full-length infectious clone of wild-type HKU5 (HKU5-1, LMH03f isolate), along with a fluorescence protein with ORF5 substituted by a ZsGreen-HiBit reporter (ZGH) (Extended Data Fig. 10j). Using the Caco2-1B4 cells, we successfully rescued both versions of HKU5-1 authentic viruses, and isolated another HKU5 strain (PaGD2014/15) from a bat anal swab sample (Fig. 4l, m and Extended Data Fig. 10k–n). Electron microscopy revealed typical morphology of “crown-shaped” HKU5-1 virions (Fig. 4k). Efficient amplification was observed in cells inoculated with HKU5-1 at different MOI, as indicated by nucleocapsid (N) protein immunostaining and the accumulation of viral RNA in the supernatant (Fig. 4l,m). Consistent with previous reports, HKU5 can weakly amplify in Vero E6 cells in the presence of exogenous trypsin (Extended Data Fig.10o)^60,61^. Two mutations (T76R and K519T) in HKU5-1 S were detected after ten passages in Caco2-1B4 cells without significant altered biological features of trypsin dependence in infecting Caco2 cells (Extended Data Fig. 10p,q). Lastly, we assessed the activities of several antiviral reagents against HKU5-1 infection in Caco2-1B4 cells. Consistent with the pseudovirus entry assay data (Extended data Fig. 9e), most inhibitors tested blocked HKU5-1 infection, especially for EK1^59^ and EK1C4 peptides (Extended data Fig. 10r–t). However, HKU5-1 infection was inhibited by Camostat but not E64d, in line with its sequence features at the typical protease cleavage sites^60^ (Extended Data Fig. 10r,t,u).

## Discussion:

**Extended Data Fig. 11 |:**
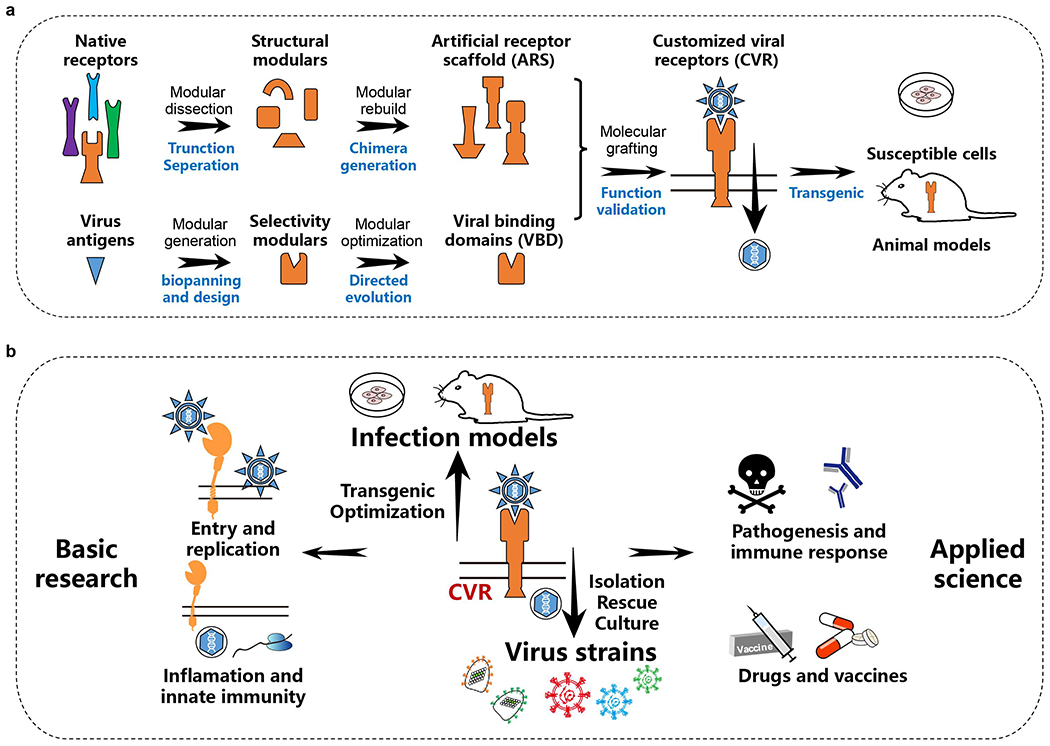
Schematic diagram of the modular design strategy for CVRs and the potential applications of this technique. **a**, Workflow outlining the process of creating artificial receptor scaffolds (ARS) and viral binding domains (VBDs) to construct functional customized viral receptors (CVRs) for establishing *in vitro* and *in vivo* infection models. **b**, The crucial role of CVRs in bridging infection models and virus strains, along with the potential applications of this technique in both basic research and applied science.

The COVID-19 pandemic has emphasized the immediate need for research on zoonotic risks of animal coronaviruses to prepare for future outbreaks. However, in-depth research and vaccine/antiviral development are impeded by the fact that we do not know the identity of the receptors used by many viruses and our resulting inability to isolate and culture these viruses^1^. Here, we introduce a technique for engineering functional viral receptors for various coronaviruses, which involves the modular design of artificial receptor scaffolds (ARS) for functionality and the customization of viral binding domains (VBDs) for selectivity (Extended Data Fig. 11). Theoretically, receptors can be designed for most naturally existing viruses if both ARS and VBD are optimized, although requirements and challenges in achieving optimal receptor function may vary among viruses. We demonstrate that single polypeptide chain structures like nanobodies or scFvs are preferred modules for VBD design compared to Fab fragments. We also highlight the potential of computational *de novo* design of VBDs to support future attempts. Besides efficient binding to the viral surface proteins, an optimal distance between the VBD and the cell membrane is crucial for CVR functionality, although this distance may vary for spacers with distinct structures, orientations, and flexibility. Additionally, we also showed the potential of the soluble adaptor strategy in supporting viral entry, achieved by a bi-specific adaptor retargeting the viruses to cell surface-displayed receptors.

The ability of specific coronavirus spike surfaces to function as receptor-binding motifs may depend on whether a VBD bound to this region can induce necessary conformational changes that lead to the activation of the fusion machinery. Therefore, CVRs targeting the S_2_ subunit, most NTD epitopes, and some CTD epitopes are nonfunctional. We found a strong connection between the CVR functionality and their ability to expose the 76E1 epitope, encompassing the S_2_’ cleavage site. This aligns with the endogenous receptor-induced allosteric S conformational changes leading to coronavirus entry^36,38,62^. However, the specific conformational changes required to expose this epitope remain unclear, although a transition of the RBD from the “down” to the “up” conformation seems crucial^9^. Consistently, CVRs recognizing the epitopes only present in the closed S trimer with the three RBD “down” (locking the spike shut), did not support entry^29,30^.

We demonstrated that coronaviruses can employ both NTD and CTD for receptor engagement, as exemplified by the NTD-targeting S2L20-CVR, as long as the aforementioned requirements are fulfilled. Additionally, we showed that MHV infection can be promoted by either NTD or CTD-targeting CVRs, suggesting the possibility of MHV, or its relatives, recognizing an alternative receptor through CTD. It is also possible that other coronaviruses rely on (or could evolve to use) NTD-mediated entry. Notably, two infection-enhancing antibodies targeting the SARS-CoV-2 NTD, CV3-13 and DH1052^44^, did not yield functional CVRs, highlighting differences in mechanisms between the soluble antibody-mediated antibody-dependent enhancement (ADE) and membrane-anchored CVRs-mediated viral entry *in vitro*. Our study has meaningful implications for understanding ADE, especially for the FcγR receptor-mediated mechanism that can be detected both *in vitro* and *in vivo^21–23^*. However, the efficiency of FcγR receptor-mediated entry is probably limited by multiple factors such as suboptimal membrane distance, suboptimal antibody concentration, neutralization effects, and the infection in FcγR endogenously expressing cells are usually non-productive^23^. This study also brings attention to the viral entry promoted by membrane-anchored antibodies, such as B cell receptors (BCR), yet the evidence of this phenomenon in vivo and its consequence remains to be investigated. Currently, there is no significant evidence of ADE restricting antibody therapy and vaccination against SARS-CoV-2. A rare case of ADE with negative outcomes in real-life situations occurs with the Feline Infectious Peritonitis Virus (FIPV), which targets macrophages^63^.

Our CVR strategy allows the manipulation of cell susceptibility to various viruses, thereby facilitating the isolation or rescue of viruses regardless of native receptor usage or cell tropism (Extended Data Fig. 11). Overcoming limitations of native receptors underscores the potential of this strategy, such as enhancing affinity, altering epitopes, adjusting specificity, changing structures, or getting rid of physiological functional interference and cell type restrictions. CVR-based infection models can be utilized as alternative tools for assessing antibodies or other antiviral reagents against viruses lacking known receptors and conventional infection models, thereby supporting pandemic preparedness. However, several limitations should be noted when employing CVR-based models. Differences in epitopes targeted and interaction modes may result in inconsistencies when evaluating neutralizing antibodies or vaccines. Furthermore, differences may exist in the entry pathway for some CVRs compared with native receptors. It is noteworthy that CVR transgenic mice could be useful for evaluating viral pathogenesis and vaccine/antiviral protection *in vivo.* However, the variations in tissue expression patterns may limit how closely this allows recapitulating natural infection, akin to k18/hACE2 mice.

To our knowledge, this study represents the first case of rescuing, isolating, and culturing coronaviruses based on genetically modified cell culture models independent of native receptors. Our findings pave the way for the design of novel viral infection models for difficult-to-culture viruses, including those beyond coronaviruses, facilitating further advances in basic research on various infectious diseases, including disease X, and accelerating the rapid development of countermeasures for the benefit of public health worldwide.

## Methods

### Cell lines

HEK293T (CRL-3216), Vero E6 (CRL-1586), A549 (CCL-185), BHK-21 (CCL-10), Caco2 (HTB-37), Neuro2a (CCL-131) and Tb 1 Lu (CCL-88) were purchased from the American Type Culture Collection (ATCC). Huh-7 (SCSP-526) was obtained from the Cell Bank of Type Culture Collection, Chinese Academy of Sciences. All cells were maintained in Dulbecco’s Modified Eagle Medium (DMEM, Monad), supplemented with 10% fetal bovine serum (FBS), and with or without 1× Penicillin G (100U/ml) /Streptomycin (100 μg/mL) (Pen/Strep). An I1-hybridoma cell line (CRL-2700), producing a neutralizing mouse monoclonal antibody against VSV-G, was cultured in minimum essential medium with Earle’s balanced salts solution, 2 mM L-glutamine (Gibco), and 5% FBS. All cell lines were incubated at 37°C in 5% CO_2_ with regular passage every 2-3 days.

### Virus and host gene sequences

All viral genome or gene sequences were sourced from GenBank or GISAID databases with the following accession numbers. Viruses: SARS-CoV-1 (NC_004718), SARS-CoV-2 (NC_045512), MERS-CoV (NC_019843), HKU3 (DQ022305), Rp3 (DQ071615), HKU5-1 LMH03f (NC_009020), HKU31 (MK907286), HKU9 (NC_009021), Zhejiang2013 (NC_025217), Rs4081 (KY417143), MHV-A59 (NC_048217), NL63 (JX504050), 229E (OQ920101), HKU1 (NC_006577), OC43 (AY391777), RmYN02 (EPI_ISL_412977), ZC45 (MG772933), RsHuB2019A (OQ503498), GX_P2V (MW532698.1), BANAL-20-52 (MZ937000.1). The spike protein for Rs4075 (KC880993), B.1.1.7/α (EPI-ISL-601443), B.1.351/β (EPI_ISL_678597), P.1/γ (EPI_ISL_906075), B.1.617.2/δ (EPI_ISL_2378732), BA.2 (EPI_ISL_7580387). Receptors: ACE2 (NM_001371415), *R.affinis* ACE2 (MT394208), DPP4 (NM_001935), APN (NM_001150), mCEACAM1a (NM_001039186), AXL (NM_001699), NRP1 (NM_001024628), SCARB1 (BC143319), KREMEN1 (NM_032045), ASGR1 (NM_001671), CD147 (AB085790), CLEC4M (KJ902090), LRRC15 (NM_001135057)^64^, TMEM106B (NM_018374)^18^, TMPRSS2 (NM_001135099). All receptor and viral gene sequences used in this study were commercially synthesized by Genewiz or GenScript. Supplementary Table 1 summarizes the detailed information on these genes.

### Plasmids

All plasmids expressing type-I transmembrane CVRs were constructed by inserting the human codon-optimized CVR sequences into a lentiviral transfer vector (pLVX-EF1a-Puro, Genewiz) with an N-terminal CD5 leading sequence (MPMGSLQPLATLYLLGMLVASVL) and C-terminal 3×FLAG tag (DYKDHD-G-DYKDHD-IDYKDDDDK). Type-II transmembrane CVRs were constructed by replacing the C-terminal ectodomains with corresponding CVR modules, along with a N-terminal 3×FLAG tag. Chimeric protein-coding sequences were generated using overlapping PCR, direct sequence synthesis, homologous recombination (Hieff Clone, YEASEN, 10922ES20), or restriction endonuclease digestion and ligation. All modules were connected with two or four amino acids (commonly G, S, T) that were encoded by one or two restriction endonuclease sites. The sequences of CVRs for illustrating the modular design strategy are summarized in Supplementary Table 2.

Plasmids expressing the S glycoproteins from various coronaviruses for VSV pseudotyping were constructed by inserting human codon-optimized S coding sequences into either the pCAGGS or pcDNA3.1(-) vectors with C-terminal 13-18 residues substituted with an HA tag (YPYDVPDYA) to enhance VSV pseudotyping efficiency and facilitate detection^65^. Several spike genes were also introduced into the pLVX-IRES-ZsGreen vectors for flow cytometry-related assays, including the scFv-mFc binding and the 76E1 epitope exposure assays.

Plasmids expressing secreted fusion proteins, including the coronavirus antigens, scFvs, and nanobodies fused with human or mouse Fc, were constructed by inserting the coding sequences into pCAGGS. These constructs featured an N-terminal CD5 secretion leading sequence (MPMGSLQPLATLYLLGMLVASVL) and a C-terminal Twin-Strep Tag II following 3×FLAG tandem sequences (WSHPQFEKGGGSGGGSGGSAWSHPQFEKGGGRSDYKDHDGDYKDHDIDYKDDDDK) for purification or detection. Plasmids encoding codon-optimized anti-ACE2 antibodies h11B11^66^, or SARS-CoV-2 S-targeting neutralizing antibodies B6, S2P6, 76E1, S2H97, S2L20, and REGN10933, LY-CoV555, S309, S2X259, S2M11, BG10-19 were constructed by integrating the heavy-chain and light-chain coding sequences into pCAGGS with an N-terminal CD5 leader sequences, respectively. For DSP-based cell-cell fusion assays, the split protein genes were inserted into pLVX-EF1a-Puro. The coding sequences for the dual reporter split proteins, namely RLuc (1-155)-sfGFP (1-157) and sfGFP (158-231)-RLuc (156-311), are previously described^13^.

### Stable cell lines

Cells stably expressing distinct CVRs and other receptors were established using lentivirus transduction and subsequent antibiotic selection. Lentiviruses carrying the target genes were generated by co-transfecting lentiviral transfer plasmid (pLVX-EF1a-Puro) with packaging plasmids pMD2G (Addgene, 12259) and psPAX2 (Addgene, 12260) into HEK293T cells through GeneTwin (Biomed, TG101). The lentivirus-containing supernatant was pooled at 24 and 48 hours post-transfection (hpt). Cell transduction was performed in the presence of 8 μg/mL polybrene. Stable cell lines were selected and maintained in a growth medium supplemented with puromycin (1 μg/mL). Typically, cells that remained stable for at least ten days were used in subsequent experiments.

### SARS-CoV-2 reactive polyclonal sera

SARS-CoV-2 polyclonal sera were obtained from vaccinated individuals (SARS-CoV-2 CoronaVac, Sinovac), approximately 21 days post-vaccination and Wuhan COVID-19 convalescents around one-year post-infection, respectively. Ethical approval for the vaccinated individuals was granted by the Ethics Committee (seal) of Beijing Youan Hospital, Capital Medical University, with approval number LL-2021-042-K. The collection of sera from Wuhan COVID-19 convalescents was conducted in collaboration with the Hubei Provincial Center for Disease Control and Prevention and Hubei Provincial Academy of Preventive Medicine (HBCDC), following written consent and under the approval of the Institutional Review Boards with the identification number 2021-012-01. Sera were heat-inactivated at 56°C for 30 minutes.

### Recombinant protein expression and purification

Proteins for binding, neutralizing, or biopanning-related assays were produced in HEK293T by transient transfection with plasmids using GeneTwin reagent (Biomed, TG101-01), following the manufacturer’s guidelines. Protein-containing supernatants were harvested every 2-3 days post-transfection, pooled, clarified, and proceeded to purification. Proteins fused with Fc were captured using Pierce Protein A/G Plus Agarose (Thermo Scientific, 20424), eluted with pH 3.0 glycine (100 mM in H_2_O), and immediately pH-balanced by 1/10 volume of UltraPure 1 M Tris-HCI, pH 8.0 (Thermo Fisher Scientific, 15568025). Proteins with Twin-Strep Tag II (strep) were enriched using Strep-Tactin XT 4Flow high-capacity resin (IBA, 2-5030-002), washed, and eluted with buffer BXT (100 mM Tris/HCl, pH 8.0, 150 mM NaCl, 1 mM EDTA, 50 mM biotin). The eluted proteins were concentrated and buffer-exchanged to PBS through ultrafiltration, aliquoted, and stored at −80°C. Protein concentrations were determined using the Omni-Easy Instant BCA Protein Assay Kit (Epizyme, ZJ102).

### Western blot

For detecting the cellular expression of CVRs or other receptors, cells were washed with PBS and lysed using RIPA buffer (50 mM Tris-pH 7.4, 150 mM NaCl, 1%TritonX-100, 0.5% sodium deoxycholate, 0.1 % SDS, 25 mM β-glycerophosphate, 1 mM EDTA, and 1 mM PMSF) on ice for 15 minutes. The lysate was clarified by centrifugation at 12,000g at 4°C for 15 minutes. The supernatant was combined with a 1:5 (v/v) ratio of 5×SDS-loading buffer and incubated at 95°C for 10 minutes. For detecting the spike packaging efficiency, the PSV-containing supernatant was concentrated with a 30% sucrose cushion (30% sucrose, 15 mM Tris-HCl, 100 mM NaCl, 0.5 mM EDTA) at 20,000×g for 1.5 hours at 4°C. The concentrated virus pellet was resuspended in 1×SDS loading buffer and incubated at 95°C for 30 minutes. For detecting the S2’ cleavage of PSV, the concentrated viruses were resuspended in DMEM in the presence of indicated concentrations of scFv-mFc or soluble ACE2 for 2 hours at 4 °C, and then treated with 10 μg/mL TPCK-trypsin (Sigma-Aldrich, T8820) for 30 minutes at 37°C, followed by mixing with a 1:5 (v/v) ratio 5×SDS-loading buffer and incubated at 95°C for 10 minutes.

After SDS-PAGE electrophoresis and PVDF membrane transfer, blots were blocked with 5% milk in PBS containing 0.1% Tween-20 (PBST) at room temperature for 1 h. Primary antibodies targeting FLAG tag (Sigma-Aldrich, F1804, 1:10,000), HA (MBL, M180-3, 1:5,000), VSV-M [23H12] (Kerafast, EB0011, 1:10,000), β-tubulin (Immunoway, YM3030, 1:10,000) and glyceraldehyde-3-phosphate dehydrogenase (GAPDH) (AntGene, ANT325, 1:10,000) were diluted in PBST with 1% milk and incubated with the blot overnight at 4 °C. The stem-helix targeting monoclonal antibody S2P6 for coronavirus spike detection was used at 1 μg/ml. After three washes with PBST, the blots were incubated with horseradish peroxidase (HRP)-conjugated secondary goat antibodies against human (Sigma-Aldrich, A0170), rabbit (Sino Biological, SSA003), or mouse (Jackson Lab, 15-035-003) IgG (1:5,000). After extensive wash, blots were visualized using the LI-COR Odyssey CLx or the Omni-ECL Femto Light Chemiluminescence Kit (EpiZyme, SQ201) and a ChemiDoc MP Imaging System (Bio-Rad). Image Lab (version 5.2.1) was utilized to analyze Western blot data. Uncropped and unprocessed full scans of gel source data are provided in Supplementary Fig. 1 and 2.

### Live-cell binding assays

For detecting coronavirus antigens binding to cell surface expressed CVRs, NTD/CTD/S_1_-Fc fusion proteins were diluted in DMEM and incubated with cells at 1 to 4 μg/mL for one hour at 37°C. Cells were washed twice with Hanks’ Balanced Salt solution (HBSS) and incubated with 2 μg/mL Alexa Fluor 594 or 488-conjugated goat anti-mouse IgG (Thermo Fisher Scientific; A32742/A32723) for visualization. For detecting the Twin-Strep Tag II labeled S-trimer or soluble ACE2 binding, the incubated cells were treated with 1 μg/mL anti-Twin-Strep Tag II monoclonal antibody (Abbkine; ABT2230) for 30 minutes at 4°C, washed twice with HBSS, and then subjected to fluorescence-labeled secondary antibody incubation. Finally, cells were incubated with Hoechst 33342 (1:5,000 in HBSS) for nuclear staining before imaging using a fluorescence microscope (MI52-N).

### Immunofluorescence for detecting receptor expression

Immunofluorescence assays were performed to assess the expression of the CVRs or other receptors carrying the C-terminal 3×FLAG tags. In general, cells expressing the proteins were fixed with 100% methanol at room temperature for 10 minutes, washed once with PBS, and incubated with a mouse monoclonal antibody M2 specific to the FLAG-tag (Sigma-Aldrich, F1804, 1:1000) in 1% BSA/PBS at 37°C for 1 hour. After another wash with PBS, cells were incubated with 2 μg/mL Alexa Fluor 594-conjugated goat anti-mouse IgG (Thermo Fisher Scientific, A32742) diluted in 1% BSA/PBS for 1 hour at 37°C. Nuclei were stained with Hoechst 33342 (1:5,000 dilution in PBS). Images were captured and merged using a fluorescence microscope (Mshot, MI52-N).

### Biolayer interferometry

Protein binding kinetics were evaluated through Bio-Layer Interferometry (BLI) assays conducted on the Octet RED96 instrument (Molecular Devices) at 25°C, and shaking at 1000 rpm. Briefly, 20 μg/mL of S_1_/NTD/CTD-hFc recombinant proteins were immobilized on protein A (ProA) biosensors (ForteBio, 18-5010). Subsequently, the biosensors were washed and incubated with 2-fold serial-diluted monomeric nanobodies (Twin-Strep Tag II only) in the kinetic buffer (PBST) to record the association kinetics, followed by recording the dissociation kinetics in the same Kinetic buffer. The background was established using a kinetic buffer without the binding proteins. The kinetic parameters and binding affinities were determined using the Octet Data Analysis software (v.12.2.0.20) with global curve fitting using a 1:1 binding model.

### VSV pseudovirus entry and inhibition assay

Single-round VSV-based pseudoviruses carrying the coronavirus spikes were produced following a modified version of a well-established protocol^67^. The VSV-ΔG carrying GFP and firefly luciferase (VSV-ΔG-fLuc-GFP) was rescued using a reverse genetics system in the laboratory, along with helper plasmids (pBS-N, pBS-P, pBS-G, and pBS-L) from Karafast. For packaging coronavirus PSV, HEK293T cells were transfected with plasmids overexpressing the spike proteins. At 24-36 hpt, cells were inoculated with 1×10^6^ TCID_50_/mL VSV-dG-fLuc-GFP for 4 hours at 37°C with 8 μg/mL polybrene. Following two DMEM washes, the culture medium was replenished with DMEM containing 1 μg/mL anti-VSV-G neutralizing antibody (from the I1-mouse hybridoma) to minimize background signals from parental viruses. The TCID_50_ of the PSV was calculated using the Reed-Muench method^68,69^.

For pseudovirus entry or entry inhibition assays, susceptible cells were cultured in 96-well plates at a density of 5×10^4^ cells per well and then incubated with around 1×10^6^ TCID_50_/mL of pseudovirus (PSV), with 100 μL per well. The incubation allowed for attachment and viral entry with or without the indicated concentrations of antibodies or other inhibitors. In some cases, TPCK-treated trypsin (Sigma-Aldrich, T8802) of indicated concentrations was added to the medium to enhance entry efficiency. At 16-20 hours post-infection (hpi), 40 μL of One-Glo-EX substrate (Promega) was added to the cells and incubated for at least 5 minutes on a plate shaker in the dark. Relative light units (RLU) were determined using the GloMax 20/20 Luminometer (Promega). GFP intensity was analyzed using a fluorescence microscope (Mshot, MI52-N).

### Recombinant VSV-CoV propagation assay

For VSV-CoV propagation assays, the propagation competent (pc) recombinant VSVs with VSV-G gene genomically replaced with HKU1, HKU3, or HKU5 spike genes (pcVSV-HKU1, pcVSV-HKU3, and pcVSV-HKU5) were generated by the VSV based reverse genetics system. The vector for the VSV genomes was modified based on pVSV-ΔG-fLuc-GFP, with fLuc replaced by the CoV S genes. In brief, the BHK-21 cells were infected with a recombinant vaccinia virus expressing T7 RNA polymerase (vvT7) for 45 minutes at 37°C (MOI=5). After removing vvT7, the cells were transfected with plasmids containing the pVSV-ΔG-GFP-HKU1/HKU3/HKU5-S vector and helper plasmids from Karafast. The virus-containing supernatant (P0) was collected 48 hpt and amplified in Vero E6 cells with in-trans provided VSV-G to yield P1 viruses. The P1 viruses were further amplified in Caco2-CVRs cells in a VSV-G independent manner and in the presence of anti-VSVG (I1-Hybridoma supernatant), generating P2 viruses that were dependent on the genomically encoded CoV S glycoproteins for amplification, which can be enhanced with the presence of 20 μg/mL TPCK-trypsin in DMEM+2% FBS.

### Production of recombinant spike glycoprotein

The prefusion SARS-CoV-2 S ectodomain contains the Wuhan-Hu-1 sequence plus Hexapro stabilizing mutations (F817P, A892P, A899P, A942P, K986P, V987P), S_1_/S_2_ cleavage site mutations (R682G, R683S, R685S) as well as C-terminal foldon trimerization domain, 3C cleavable HIS- and StrepII- tags^50^. For triggering assays, we used a construct encoding SARS-CoV-2 S G614 with a mu-phosphatase signal peptide placed before S residue Q14, with a mutated S_1_/S_2_ cleavage site (S_682_GAR_685_), and ending at residue K1211 followed by a TEV cleavage site, a foldon trimerization motif, and an 8× His tag cloned in a pCMV vector^70,71^. S trimers were produced in 200 mL cultures of Expi293F Cells (ThermoFisher Scientific) grown in suspension using Expi293 Expression Medium (ThermoFisher Scientific) at 37°C in a humidified 8% CO2 incubator rotating at 130 rpm. Cells grown to a density of 3 million cells per mL were transfected using the ExpiFectamine 293 Transfection Kit (ThermoFisher Scientific) and cultivated for 5 days at which point the supernatant was harvested. S ectodomains were purified from clarified supernatant using a Cobalt affinity column (Cytiva, HiTrap TALON crude), washing with 20 column volumes of 50 mM Tris-HCl pH 8.0, 150 mM NaCl, and 1 mM imidazole, and eluted with 50 mM Tris-HCl pH 8.0, 150 mM NaCl, and 600 mM imidazole. The S ectodomain was then concentrated using a 100 kDa centrifugal filter (Amicon Ultra 0.5 mL centrifugal filters, MilliporeSigma), residual imidazole was washed away by consecutive dilutions in the centrifugal filter unit with 50 mM Tris-HCl pH 8.0 and 150 mM NaCl, and finally concentrated to 1 mg/mL for use immediately after purification.

### CryoEM sample preparation and data collection

100 μL of 1 mg/mL prefusion-stabilized SARS-CoV-2 S ectodomain was incubated with 2.2 μL of 67 mg/mL S2L20 Fab for 15 min at 37°C. Most unbound Fab was then washed away with six consecutive dilutions in 400 μL of 50 mM Tris-HCl pH 8.0 and 150 mM NaCl and concentration using a 100 kDa centrifugal filter (Amicon Ultra 0.5 mL centrifugal filters, MilliporeSigma). The complex was concentrated to 3.5 mg/mL and 3 μL was immediately applied onto a freshly glow discharged 2.0/2.0 UltraFoil grid^72^ (200 mesh), plunge frozen using a vitrobot MarkIV (ThermoFisher Scientific) using a blot force of −1 and 6.0 s blot time at 100% humidity and 23°C. Data were acquired using the Leginon software^73^ to control an FEI Titan Krios transmission electron microscope equipped with a Gatan K3 direct detector and operated at 300 kV with a Gatan Quantum GIF energy filter. The dose rate was adjusted to 3.75 counts/super-resolution pixel/s, and each movie was acquired in 75 frames of 40 ms with a pixel size of 0.843 Å and a defocus range comprised between −0.2 and −2.0 μm.

### CryoEM data processing

Movie frame alignment (with a downsampled pixel size of 1.686 Å) was carried out using Warp^74^. Estimation of the microscope contrast-transfer function parameters followed by manual micrograph curation, Blob particle picking, particle extraction (with a box size of 256 pixels^2^), reference-free 2D classification to generate templates for template-based particle picking (templates and micrographs low-pass filtered to 20 Å resolution during template-based particle picking), particle re-extraction, and then reference-free 2D classification were performed in cryoSPARC^75^. Well-defined particle images ab initio reconstruction followed by non-uniform 3D refinement in CryoSPARC^76^ before subjecting particle images to Bayesian polishing using Relion^77^ during which particles were re-extracted with a box size of 512 Å at a pixel size of 0.843 Å. Reference-free 2D classification and non-uniform refinement in CryoSPARC followed by Bayesian polishing in Relion was repeated. At this stage, 3D classification with 50 iterations each (angular sampling 7.5 for 25 iterations and 1.8 with local search for 25 iterations) was carried out using Relion without imposing symmetry^78^. Next, 42 optics groups were defined based on the beam tilt angle used for data collection, and another round of non-uniform refinement in CryoSPARC was then performed concurrently with defocus refinement and Global CTF refinement, fitting beam-tilt and trefoil^79^. For focused classification of the NTD, particles were symmetry-expanded and 3D classified in Relion without alignment using a mask that encompasses the NTD and the S2L20 VH/VL region. Particles in well-defined 3D classes were then used for local refinement in CryoSPARC. Reported resolutions are based on the gold-standard Fourier shell correlation of 0.143 criterion and Fourier shell correlation curves were corrected for the effects of soft masking by high-resolution noise substitution^80,81^.

### CryoEM model building and analysis

UCSF Chimera^82^ and Coot^83^ were used to fit atomic models of S2L20 and SARS-CoV-2 S (PDB 7SOB and 7LXY) into the CryoEM maps. The model was then refined and rebuilt into the map using Coot^83^, Rosetta^84,85^, and ISOLDE^86^. Model validation and analysis used MolProbity^87^, EMRinger^88^, Privateer^89^, and Phenix version 1.21^90^. Figures were generated using UCSF ChimeraX^91^.

### Triggering assay

For antibody-triggered S refolding, 20 ʼl of 0.21 mg/ml SARS-CoV-2 S G614 ectodomain (without 2P or Hexapro stabilizing mutations) was incubated at 37 ºC for 1 hour with and without 0.21 mg/ml S2H14, S2X28, or S2L20, immediately prior to negative staining. Samples were diluted to 0.01 mg/mL immediately prior to adsorption to glow-discharged carbon-coated copper grids for 30 sec prior to staining with 2% uranyl formate. Micrographs were recorded using the Leginon software^73^ on a 120 kV FEI Tecnai G2 Spirit with a Gatan Ultrascan 4000 4k x 4k CCD camera at 67,000 nominal magnification. The defocus ranged from -1.0 to -2.0 ʼm and the pixel size was 1.6 Å.

### Cell-cell fusion assays

A cell-cell fusion assay based on dual split proteins (DSPs) was performed on HEK293T or Caco2 cells stably expressing the CVRs or the native receptors^13^. Group A cells were transfected (Lipofectamine 2000, Biosharp) with plasmids expressing spike proteins and RLucN (1-155)-sfGFP(1-157), while the group B cells were transfection with plasmids expressing spike proteins (same as in group A) and sfGFP(158-231)-RLuc(156-311). Cells from both groups were trypsinized and co-cultured in a 96-well plate at a density of approximately 1×10^5^ cells per 96-well 12 hpt. After 24 hours, cells were washed once by DMEM to remove FBS. Following incubation of DMEM with or without 100 μg/mL of TPCK-treated trypsin at 37°C for 15 minutes, and subsequent culture in DMEM+10% FBS for 24-36 hours, cell nuclei were stained with Hoechst 33342 (1:5,000 dilution in HBSS) for 30 minutes at 37 °C, and then fluorescence imaging using a fluorescence microscope (MI52-N; Mshot). For the assessment of live-cell luciferase activity after reconstitution of split RlucN, 20 μM of EnduRen live-cell substrate (Promega, E6481) was added to the cells in DMEM and incubated for at least 1 hour before detection using the Varioskan LUX Multi-well Luminometer (Thermo Fisher Scientific).

### Flow cytometry analysis

For live cell binding assays analyzing CoVs S_1_/RBD-mFc recombinant proteins binding, proteins were diluted in DMEM at the indicated concentrations and then incubated with HEK293T cells expressing the CVRs for 1 hour at 37°C, followed by incubation with Alexa Fluor 594-conjugated goat anti-mouse IgG (Thermo Fisher Scientific; A32742; 1:5000) detecting antigen binding and Alexa Fluor 488-conjugated goat anti-human IgG (Thermo Fisher Scientific; A11013; 1:5000) detecting CVRs (with cell surface hFc) expression. If hFc is absent, the intracellular FLAG tags were utilized to assess the receptor expression. Specifically, cells were washed once with HBSS and fixed with 4% PFA, permeabilized with 0.1% Triton X-100, blocked with 1% BSA/PBS at 4°C for 30 minutes, and subsequently stained with Rabbit anti-Flag tag mAb (CST, 14793S; 1:1000) diluted in 1% BSA/PBS for 1 hour at 4°C. Following extensive washing, the cells were incubated with Alexa Fluor 647-conjugated goat anti-rabbit IgG (Thermo Fisher Scientific; A32733; 1:5000) and Alexa Fluor 488-conjugated goat anti-mouse IgG (Thermo Fisher Scientific; A32723; 1:5000) for 1 hour at 4°C for detecting both FLAG tags and SARS-CoV-2 RBD-mFc binding.

For scFv-mFc binding assays, the SARS2-CoV-2-S IRES-ZsGreen expressing cells were incubated with indicated concentrations of scFv-mFc for 1 hour at 37°C in DMEM/1%BSA, washed once with PBS, and then incubated with Alexa Fluor 594-conjugated goat anti-mouse IgG (Thermo Fisher Scientific; A32742; 1:5000). For 76E1 epitope exposure assays, the SARS2-CoV-2-S IRES-ZsGreen expressing cells were first incubated with scFv-mFc or soluble ACE2 for 1 hour in DMEM/1%BSA, washed once and then incubated with 76E1 (1 μg/mL) at 37°C for another hour in DMEM/1%BSA. Subsequently, the washed cells were incubated with DyLight 594-conjugated goat anti-human IgG (Thermo Fisher Scientific; SA5-10136; 1:5000) to detect 76E1 binding.

After all staining procedures, cells were washed twice with PBS and then analyzed using the CytoFLEX Flow Cytometer (Beckman). In each case, 5,000 cells expressing either receptors or spikes, gated based on FLAG/hFc/ZsGreen-fluorescence and SSC/FSC, were analyzed with the CytoFLEX Flow Cytometer (Beckman). FlowJo (version 10) was employed to analyze the flow cytometry data. The representative gating strategies for each experiment are demonstrated in Supplementary Fig.3.

### HKU5 reverse genetics

The full-length cDNA clone of HKU5 (strain: HKU5-1 LMH03f, GenBank: NC_009020) was designed and synthesized as seven (from A to G) contiguous cDNAs flanked by unique class IIS restriction endonuclease site (BsaI or BsmBI) and cloned in pUC57 vector. Class II restriction endonuclease sites AvrII and AscI were introduced to the 5’ terminal of HKU5 A and the 3’ terminal of HKU5 G fragments, respectively. Several silent mutations were included to disrupt naturally occurring restriction cleavage sites. A poly-A (25 repeats) sequence was introduced to the 3’ terminal of the HKU5 G fragment. To assemble the full-length cDNA clone, HKU5 A-G fragments were digested by endonucleases, resolved on 1% agarose gels, purified with a gel extraction kit, extracted with chloroform, and precipitated with isopropyl alcohol. Digested HKU5 A-G inserts, and modified pBaloBAC11 vector were mixed, ligated overnight at 4°C, and transformed into DH10B competent cells. The correct full-length HKU5 cDNA clone was identified and verified by sequencing. The construction of HKU5-ZGH utilized the transformation-associated recombination (TAR) cloning technique. Specifically, a ZsGreen-HiBit (ZGH) DNA fragment was commercially synthesized (Tsingke) to replace the HKU5-ORF5. The PCC1 vector was used to clone the HKU5 genomic DNA carrying the ZGH substitution based on three segments amplified using the HKU5-WT infectious clone as a temperate. Subsequently, all the products were transformed into yeast using the high-efficiency lithium acetate/SS carrier DNA/PEG method. The yeast plasmid was extracted and transformed into EPI300 electrocompetent cells. The plasmid used for cell transfection was obtained from a 300 mL E. coli bacterial culture suspension. For transfection, 4 μg of both HKU5 WT and HKU5-ZGH plasmids were separately transfected into Caco2-1B4 cells (1×10^6^ cells) using GeneTwin (Biomed, TG101). Progeny viruses collected from the supernatant at 72 hpt (P0) were utilized to generate stocks for subsequent analyses.

### HKU5 sampling and isolation

Bats were trapped in their natural habitat as previously described^92,93^. An anal swab (Sample ID: NL140575) with confirmed HKU5 genomic sequence (strain HKU5_PaGD2014/15) was collected from a *Pipistrellus abramus* bat in Guangzhou City, Guangdong province on June 28, 2014. The sample was preserved in viral transport medium (VTM) comprising Hank’s balanced salt solution, BSA (1%), amphotericin (15 μg/mL), penicillin G (100 U/mL), and streptomycin (50 μg/mL)^94^, transported to the laboratory and stored at −80°C until use. All bats trapped for this study were released back to their habitat after anal swab collection. All sampling processes were carried out by veterinarians with approval from the Animal Ethics Committee of the Wuhan Institute of Virology (WIVH05210201).

To isolate the HKU5, the sample preserved in VTM was centrifuged at 10,000g at 4°C for 10 minutes. Then, 100μl of the 0.45 μm-filtered sample was mixed with 100 μL DMEM + 2% FBS + 2× Pen/Strep and incubated with Caco2-1B4 cells at 37°C for 2 hours. This was followed by supplementation of 400 μL fresh DMEM + 2% FBS + 2× Pen/Strep. The cytopathic effect was monitored daily, and the supernatant (passage 1, P1) was collected on day 5 post-infection. Subsequently, 200 μL of the P1 supernatant was incubated with freshly plated Caco2-1B4 cells at 37°C for 1 hour. The inocula were then removed and replenished with 400 μL fresh DMEM with 2% FBS. The HKU5-containing supernatant (passage 2, P2) was collected on day 3 and day 6 post-infection and stored at −80°C. The Caco2 or Caco2-1B4 cells were challenged with P2 supernatant (collected on day 6) and fixed with methanol for 40 minutes at room temperature on day 3 post-infection, and HKU5 N proteins were detected by rabbit anti-HKU5 N protein serum (diluted at 1:4000), followed by Cy3-conjugated goat anti-rabbit IgG (Abcam, 1:250) staining. All HKU5 isolation-related experiments were conducted in the certified negative-pressure Biosafety Level 2 laboratory at the Wuhan Institute of Virology.

### Transmission electron microscopy

Viral culture supernatant was fixed with formaldehyde (working concentration 0.1%) at 4°C overnight. Subsequently, it was concentrated by ultracentrifugation through OptiPrepTM Density Gradient Medium (D1556) at 154,000 g at 4°C for 2.5 hours using a SW41Ti rotor (Beckman). The pelleted viral particles were suspended in 100 μL of PBS, stained with 2% phosphotungstic acid (pH 7.0), and examined using a transmission electron microscope (Thermo Fisher, Talos L120C) at 120 kV.

### Authentic coronavirus infection assays

Human coronavirus 229E (VR-740) is obtained from ATCC and amplified in Huh-7 cells. MHV-A59 is a gift from Professor Yu Chen’s lab (Wuhan University) and is amplified in Neuro2a cells. The SARS-CoV-2-(ΔN-GFP) with N protein substituted with GFP is rescued using an established protocol, and cultured in Caco2 cells overexpressing the SARS-CoV-2 N protein^95^. All experiments involving RsHuB2019A, and HKU5-related authentic viruses infection were conducted in the certified negative-pressure Biosafety Level 2 laboratory at Wuhan Institute of Virology. RsHuB2019A is amplified in either Huh-7 or in Caco2-Nb27 cells. HKU5-related strains are amplified in Caco2-1B4 cells.

For authentic virus infection assays, target cells were seeded in 24-well plates, washed once with DMEM, and then inoculated with the indicated MOI of authentic viruses for 1 hour. Subsequently, the infected cells were washed with DMEM once and replenished with DMEM with or without 2% FBS. RsHuB2019A infection was conducted in Huh-7 cells in DMEM (must be FBS-free) with 100 μg/mL trypsin (Sigma-Aldrich, T4549). RsHuB2019A or HKU5-1 infection in Caco2-CVR cells was conducted with or without the presence of 100-200 μg/mL trypsin (in DMEM+2% FBS) or 5-10 μg/mL trypsin (in DMEM without FBS) during and post-inoculation. For qRT-PCR analysis of viral RNA accumulation, cell-free supernatants (50 μL per well each time) were collected at indicated time points post-infection and stored at −80°C. Viral RNA was extracted using Virus DNA/RNA Extraction Kit (Vazyme: RM501) and subjected to qRT-PCR as previously described^96^. qRT-PCR was conducted using the C1000 Touch Thermal Cycler/CFX96 Real-time system (Bio-Red) and analyzed with Bio-Rad CFX Maestro (4.1.2433.1219) software. Primers for RsHuB2019A RdRp: 5’-TTGTTCTTGCTCGCAAACATA-3’ (forward) and 5’-CACACATGACCATCTCACTTAA-3’ (reverse). Primer for HKU5 nsp2: 5’-CTGCGCTTAATGCCCCATTC-3’ (forward) and 5’-GACGTGTAGACGTAGAGCCG-3’ (reverse). Primers for VSV L protein, forward primer: 5’-TCTTGAGTTGTGGAGACGGC-3’ (forward) and 5’- ACCGTCTTGAACATGGGACC-3’ (reverse). Primers for MHV-A59 N protein:5’-TATAAGAGTGATTGGCGTCC-3’(forward) and 5’-GAGTAATGGGGAACCACACT-3’ (reverse). All samples were analyzed in duplicate on two independent runs.

For immunofluorescence assays, cells were fixed with 4% PFA for at least 20 minutes at 25°C for 24-48 hpi at specified time points. N proteins for RsHuB2019A and HKU5 were detected using rabbit anti-Rp3 N protein serum (1:2000) and rabbit anti-HKU5 N protein serum (1:4000), respectively, followed by Cy3-conjugated goat anti-rabbit IgG (Abcam, 1:250) staining. Images were captured using an EVOS M5000 fluorescence microscope (Thermo Fisher Scientific). For MHV-A59 and 229E-VR740, spike proteins were detected using their spike-targeting nanobodies (1A1-mFc for 229E-VR740; 1F7-mFc for MHV-A59), followed by DL594-conjugated goat anti-mouse IgG antibodies (Thermo Fisher, 1:1000). Images were captured and merged using a fluorescence microscope (Mshot, MI52-N). The titer of the authentic viruses was determined by immunofluorescence at 4 days post-infection, and the TCID_50_ was calculated using the Reed-Muench method^68,69^.

### Nanobody bio-panning

Specific viral antigens (30-100 μg) were immobilized on streptavidin-conjugated magnetic beads for one-hour incubation at 37°C and extensively washed to remove unbound antigens. Subsequently, the beads were incubated with the nanobody library (1×10^10^ PFU) (Naïve VHH libraries from Camelus bactrianus, Alpaca, and Llama from NBbiolab, China) for 1 hour. The bound phages were eluted using an Elution Buffer (50 mM Tris-pH 7.4, 150 mM NaCl, 50 mM biotin) after extensive washing with PBST to eliminate nonspecific binders. The eluted phage encoding the specific nanobodies was proliferated in E. coli (TG1). After one round of magnetic beads-based selection, 1-3 additional rounds of phage biopanning were conducted using magnetic beads or immunotubes. The positive clones were identified through the enzyme-linked immunosorbent assays (ELISA), sequenced, and verified by cell-based binding assays. Nanobody information was summarized in Supplementary Table 3.

### Biosafety statement

Experiments related to authentic human coronavirus 229E, SARS-CoV-2-ΔN, and murine MHV-A59 were authorized by the Biosafety Committee of the State Key Laboratory of Virology, Wuhan University and conducted in accordance with standard operating procedures (SOPs) in a BSL-2 laboratory. SARS-CoV-2 authentic viruses-related experiments were conducted in the ABSL-3 facility at Wuhan University with the approval of the Biosafety Committee of the ABSL-3 laboratory. The SARS-CoV-2 WT strain (IVCAS 6.7512) was provided by the National Virus Resource, Wuhan Institute of Virology, Chinese Academy of Sciences and amplified in Vero E6 cells in the ABSL-3 facility at Wuhan University. Experiments related to authentic viruses RsHuB2019A, HKU5-WT, and HKU5-ZGH were approved by the Wuhan Institute of Virology (WIV) IBCs and performed in the BSL-2 laboratory according to SOPs at WIV facilities. *In vitro* experiments related to RsHuB2019A infection, HKU5-1 (LMH03f) rescue, and HKU5 (PaGD2014/15 strains) isolation were performed in the BSL-2 laboratory following SOPs with necessary personal protection and approved by the Wuhan Institute of Virology (WIV) IBCs according to a biorisk assessment procedure. *In vitro* experiments involving these bat coronaviruses were assessed as BSL-2 level primarily based on their narrow cell tropism and limited efficiency in using human receptors. The complete assessment includes the viral genome sequence and its phylogenetic relationship to known human viruses, human receptor utilization, cell tropism, prevalence in domestic animals and humans, and pathogenicity and transmission in animal models. All the facilities at both Wuhan University and WIV for this work adhere strictly to the safety requirements recommended by the China National Accreditation Service for Conformity Assessment.

We recommend that future research activities involving the use of CVR-expressing cells to isolate, rescue, or propagate uncharacterised authentic viruses should be authorized first and strictly adhere to biosafety requirements based on the biorisk assessment conducted by the institutional Biosafety Committee on a case-by-case basis, in accordance with the biosafety regulations of different countries. We recommend experiments based on pseudoviruses or replication-deficient viruses be conducted first whenever possible to gather essential data for biorisk assessment before isolating or rescuing authentic viruses. Considering the potential mutations during the propagation of viruses in human cells, biological feature changes during passages should be closely monitored, and viral genome sequencing should be conducted to check whether key adaptive mutations occurred. Furthermore, non-human cells expressing CVR can also be used for virus propagation to minimize the risk of viruses adapting to human cells. Large-scale viral propagation of the newly isolated/rescued uncharacterized viruses and initial virus characterization in animal models are suggested to be performed in a BSL-3 containment. The biorisk classification and containment levels of previously uncharacterized viruses should be re-assessed based on newly acquired data related to the biological and epidemiological characterizations to comply with the biosafety regulations of the country.

### Bioinformatic and computational analyses

Multi-sequence alignment was analyzed by Mafft (v7.313) and Geneious Prime (2023.0.1) with default parameters. Phylogenetic trees were constructed by IQ-TREE (v2.0.6) with the WAG substitution model (1,000 Bootstraps) and rendered with iTOL (v6) (http://itol.embl.de).

### Statistical analysis and reproducibility

Most experiments were repeated independently 2-4 times with similar results, each with approximately 2-4 biologically independent replicates (most are n=3). Results are presented as mean ± standard deviation (s.d.) as specified in the figure legends. Statistical analyses were primarily performed using GraphPad Prism (Version 8.0.2) through unpaired two-tailed Student’s *t*-tests for two independent groups or One-way Analysis of variance (ANOVA) analysis followed by Dunnett’s test for multiple comparisons. No adjustments were made for all multiple comparisons. *P*<0.05 was considered statistically significant; *P<0.05; **P<0.01; ***P<0.001; ****P< 0.0001; NS, non-significant, *P*>0.05.

## Extended Data

**Extended Data Table 1 |:** CryoEM data collection, processing, and model refinement statistics

**Data collection and processing**		

Magnification (nominal)	130,000	

Voltage (kV)	300	

Electron exposure (e^−^/Å^2^)	63	

Defocus range (μm)	0.3-2.0	

Pixel size (Å)	0.843	

Processing type	Global	Local

Symmetry imposed	*C*3	none

Initial particle images (no.)	381,075	497,199

Final particle images (no.)	165,733	209,164

Map resolution (Å)	2.6	3.2
FSC threshold	0.143	0.143

Map sharpening *B* factor (Å^2^)	85.7	114.8

		

**Refinement**		

Initial model used (PDB code)	7LXY	7SOB

Model resolution (Å)	2.8	3.4
FSC threshold	0.5	0.5

Model composition		
Nonhydrogen atoms	26,337	4006
Protein residues	3,768	496
Glycan residues	60	9

*B* factors (Å^2^)		
Protein	34.9	17.0
Glycans	22.7	21.9

R.m.s. deviations		
Bond lengths (Å)	0.01	0.01
Bond angles (°)	1.03	1.03

		

**Validation**		

MolProbity score	0.8	0.7

Clashscore	0.83	0.26

Rotamer outliers (%)	0.3	0.0

Privateer glycan issues (%)	0.0	0.0

Ramachandran plot		
Favored (%)	98.0	97.5
Allowed (%)	2.0	2.5
Outliers (%)	0.0	0.0

EMRinger score	4.9	5.1

		

**Data Availability**		

EMDB	EMD-45174	EMD-45175

## Supplementary Material

PDB Validation Report 9C44

PDB Validation Report 9C45

SI guide

Supp figures 1 to 3

Supp table 1

Supp table 2

Supp table 3

Source Data Fig1

Source Data Fig2

Source Data Fig3

Source Data Fig4

Source Data ED Fig1

Source Data ED Fig2

Source Data ED Fig3

Source Data ED Fig4

Source Data ED Fig5

Source Data ED Fig7

Source Data ED Fig8

Source Data ED FIg9

Source Data ED Fig10

Supplementary Information is available for this paper.

## Data Availability

The CryoEM maps and models have been deposited to the Electron Microscopy Data Bank and Protein Data Bank (PDB) with accession numbers EMD-45174/PDB ID 9C44 (global refinement of the S2L20-bound S trimer) and EMD-45175/PDB ID 9C45 (local refinement of the NTD and S2L20 Fab variable regions). The accession numbers (NCBI GenBank or GISAID), and protein sequence information of receptor, virus, antibody, domains, and reporter genes are provided in the Methods and Supplementary Tables. All other data supporting the findings of this study are available with the Article and the Supplementary Information. Source data are provided with this paper.
