# Supplementary material for: Design of customized coronavirus receptors
Source: Nature. Author manuscript; Available in PMC 2025 Jun 24. (PMC12187079; doi:10.1038/s41586-024-08121-5)
Supplement: PDB Validation Report 9C44 [file NIHMS2083933-supplement-PDB_Validation_Report_9C44.pdf]

Full wwPDB EM Validation Report (i)

Jun 4, 2024 – 11:40 AM EDT

PDB ID	:	9C44
EMDB ID	:	EMD-45174
Title	:	SARS-CoV-2 S + S2L20
Deposited on	:	2024-06-03
Resolution	:	2.60 Å(reported)

This wwPDB validation report is for manuscript review

For Manuscript Review

This is a Full wwPDB EM Validation Report.

This report is produced by the wwPDB biocuration pipeline after annotation of the structure.

We welcome your comments at *validation@mail.wwpdb.org*
A user guide is available at
https://www.wwpdb.org/validation/2017/EMValidationReportHelp
with specific help available everywhere you see the (i) symbol.

The types of validation reports are described at
http://www.wwpdb.org/validation/2017/FAQs#types.

The following versions of software and data (see references (i)) were used in the production of this report:

EMDB validation analysis	:	0.0.1.dev92
Mogul	:	1.8.5 (274361), CSD as541be (2020)
MolProbity	:	4.02b-467
Percentile statistics	:	20191225.v01 (using entries in the PDB archive December 25th 2019)
MapQ	:	1.9.13
Ideal geometry (proteins)	:	Engh & Huber (2001)
Ideal geometry (DNA, RNA)	:	Parkinson et al. (1996)
Validation Pipeline (wwPDB-VP)	:	2.36.2

1 Overall quality at a glance (i)

The following experimental techniques were used to determine the structure:
ELECTRON MICROSCOPY

The reported resolution of this entry is 2.60 Å.

Percentile scores (ranging between 0-100) for global validation metrics of the entry are shown in the following graphic. The table shows the number of entries on which the scores are based.

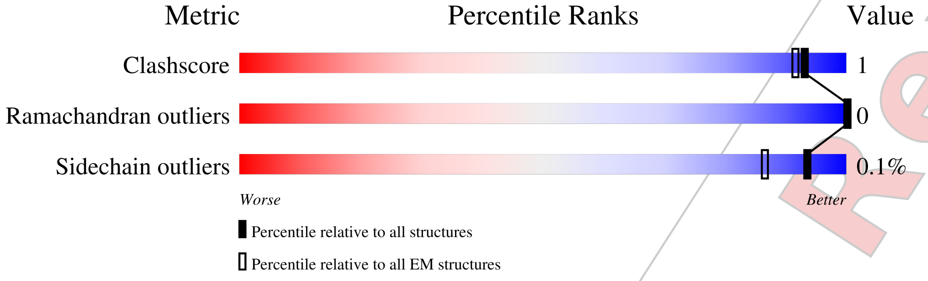

Metric	Whole archive (#Entries)	EM structures (#Entries)
Clashscore	158937	4297
Ramachandran outliers	154571	4023
Sidechain outliers	154315	3826

The table below summarises the geometric issues observed across the polymeric chains and their fit to the map. The red, orange, yellow and green segments of the bar indicate the fraction of residues that contain outliers for >=3, 2, 1 and 0 types of geometric quality criteria respectively. A grey segment represents the fraction of residues that are not modelled. The numeric value for each fraction is indicated below the corresponding segment, with a dot representing fractions <=5% The upper red bar (where present) indicates the fraction of residues that have poor fit to the EM map (all-atom inclusion < 40%). The numeric value is given above the bar.

Mol	Chain	Length	Quality of chain
1	A	1288	18% / 76% • 22%
1	B	1288	18% / 76% • 22%
1	F	1288	18% / 76% • 22%
2	C	121	31% / 100%
2	D	121	30% / 100%
2	G	121	32% / 100%
3	E	107	64% / 98% •
3	H	107	66% / 100%

Continued on next page...

For Manuscript Review

Continued from previous page...

Mol	Chain	Length	Quality of chain
3	L	107	65% / 98% •
4	I	2	100% / 100%
4	K	2	100% / 100%
4	N	2	100% / 100%
5	J	3	100% / 67% 33%
5	M	3	100% / 67% 33%
5	O	3	100% / 67% 33%

For Manuscript Review

2 Entry composition (i)

There are 6 unique types of molecules in this entry. The entry contains 25845 atoms, of which 0 are hydrogens and 0 are deuteriums.

In the tables below, the AltConf column contains the number of residues with at least one atom in alternate conformation and the Trace column contains the number of residues modelled with at most 2 atoms.

- Molecule 1 is a protein called Spike glycoprotein.

Mol	Chain	Residues	Atoms					AltConf	Trace
			Total	C	N	O	S		
1	A	1008	6968	4420	1195	1319	34	0	0
1	B	1008	6968	4420	1195	1319	34	0	0
1	F	1008	6968	4420	1195	1319	34	0	0

There are 267 discrepancies between the modelled and reference sequences:

Chain	Residue	Modelled	Actual	Comment	Reference
A	682	GLY	ARG	engineered mutation	UNP P0DTC2
A	683	SER	ARG	engineered mutation	UNP P0DTC2
A	685	SER	ARG	engineered mutation	UNP P0DTC2
A	817	PRO	PHE	engineered mutation	UNP P0DTC2
A	892	PRO	ALA	engineered mutation	UNP P0DTC2
A	899	PRO	ALA	engineered mutation	UNP P0DTC2
A	942	PRO	ALA	engineered mutation	UNP P0DTC2
A	986	PRO	LYS	engineered mutation	UNP P0DTC2
A	987	PRO	VAL	engineered mutation	UNP P0DTC2
A	1209	GLY	-	expression tag	UNP P0DTC2
A	1210	SER	-	expression tag	UNP P0DTC2
A	1211	GLY	-	expression tag	UNP P0DTC2
A	1212	TYR	-	expression tag	UNP P0DTC2
A	1213	ILE	-	expression tag	UNP P0DTC2
A	1214	PRO	-	expression tag	UNP P0DTC2
A	1215	GLU	-	expression tag	UNP P0DTC2
A	1216	ALA	-	expression tag	UNP P0DTC2
A	1217	PRO	-	expression tag	UNP P0DTC2
A	1218	ARG	-	expression tag	UNP P0DTC2
A	1219	ASP	-	expression tag	UNP P0DTC2
A	1220	GLY	-	expression tag	UNP P0DTC2
A	1221	GLN	-	expression tag	UNP P0DTC2
A	1222	ALA	-	expression tag	UNP P0DTC2
A	1223	TYR	-	expression tag	UNP P0DTC2

Continued on next page...

For Manuscript Review

Continued from previous page...

Chain	Residue	Modelled	Actual	Comment	Reference
A	1224	VAL	-	expression tag	UNP P0DTC2
A	1225	ARG	-	expression tag	UNP P0DTC2
A	1226	LYS	-	expression tag	UNP P0DTC2
A	1227	ASP	-	expression tag	UNP P0DTC2
A	1228	GLY	-	expression tag	UNP P0DTC2
A	1229	GLU	-	expression tag	UNP P0DTC2
A	1230	TRP	-	expression tag	UNP P0DTC2
A	1231	VAL	-	expression tag	UNP P0DTC2
A	1232	LEU	-	expression tag	UNP P0DTC2
A	1233	LEU	-	expression tag	UNP P0DTC2
A	1234	SER	-	expression tag	UNP P0DTC2
A	1235	THR	-	expression tag	UNP P0DTC2
A	1236	PHE	-	expression tag	UNP P0DTC2
A	1237	LEU	-	expression tag	UNP P0DTC2
A	1238	GLY	-	expression tag	UNP P0DTC2
A	1239	ARG	-	expression tag	UNP P0DTC2
A	1240	SER	-	expression tag	UNP P0DTC2
A	1241	LEU	-	expression tag	UNP P0DTC2
A	1242	GLU	-	expression tag	UNP P0DTC2
A	1243	VAL	-	expression tag	UNP P0DTC2
A	1244	LEU	-	expression tag	UNP P0DTC2
A	1245	PHE	-	expression tag	UNP P0DTC2
A	1246	GLN	-	expression tag	UNP P0DTC2
A	1247	GLY	-	expression tag	UNP P0DTC2
A	1248	PRO	-	expression tag	UNP P0DTC2
A	1249	GLY	-	expression tag	UNP P0DTC2
A	1250	HIS	-	expression tag	UNP P0DTC2
A	1251	HIS	-	expression tag	UNP P0DTC2
A	1252	HIS	-	expression tag	UNP P0DTC2
A	1253	HIS	-	expression tag	UNP P0DTC2
A	1254	HIS	-	expression tag	UNP P0DTC2
A	1255	HIS	-	expression tag	UNP P0DTC2
A	1256	HIS	-	expression tag	UNP P0DTC2
A	1257	HIS	-	expression tag	UNP P0DTC2
A	1258	SER	-	expression tag	UNP P0DTC2
A	1259	ALA	-	expression tag	UNP P0DTC2
A	1260	TRP	-	expression tag	UNP P0DTC2
A	1261	SER	-	expression tag	UNP P0DTC2
A	1262	HIS	-	expression tag	UNP P0DTC2
A	1263	PRO	-	expression tag	UNP P0DTC2
A	1264	GLN	-	expression tag	UNP P0DTC2
A	1265	PHE	-	expression tag	UNP P0DTC2

Continued on next page...

For Manuscript Review

Continued from previous page...

Chain	Residue	Modelled	Actual	Comment	Reference
A	1266	GLU	-	expression tag	UNP P0DTC2
A	1267	LYS	-	expression tag	UNP P0DTC2
A	1268	GLY	-	expression tag	UNP P0DTC2
A	1269	GLY	-	expression tag	UNP P0DTC2
A	1270	GLY	-	expression tag	UNP P0DTC2
A	1271	SER	-	expression tag	UNP P0DTC2
A	1272	GLY	-	expression tag	UNP P0DTC2
A	1273	GLY	-	expression tag	UNP P0DTC2
A	1274	GLY	-	expression tag	UNP P0DTC2
A	1275	GLY	-	expression tag	UNP P0DTC2
A	1276	SER	-	expression tag	UNP P0DTC2
A	1277	GLY	-	expression tag	UNP P0DTC2
A	1278	GLY	-	expression tag	UNP P0DTC2
A	1279	SER	-	expression tag	UNP P0DTC2
A	1280	ALA	-	expression tag	UNP P0DTC2
A	1281	TRP	-	expression tag	UNP P0DTC2
A	1282	SER	-	expression tag	UNP P0DTC2
A	1283	HIS	-	expression tag	UNP P0DTC2
A	1284	PRO	-	expression tag	UNP P0DTC2
A	1285	GLN	-	expression tag	UNP P0DTC2
A	1286	PHE	-	expression tag	UNP P0DTC2
A	1287	GLU	-	expression tag	UNP P0DTC2
A	1288	LYS	-	expression tag	UNP P0DTC2
B	682	GLY	ARG	engineered mutation	UNP P0DTC2
B	683	SER	ARG	engineered mutation	UNP P0DTC2
B	685	SER	ARG	engineered mutation	UNP P0DTC2
B	817	PRO	PHE	engineered mutation	UNP P0DTC2
B	892	PRO	ALA	engineered mutation	UNP P0DTC2
B	899	PRO	ALA	engineered mutation	UNP P0DTC2
B	942	PRO	ALA	engineered mutation	UNP P0DTC2
B	986	PRO	LYS	engineered mutation	UNP P0DTC2
B	987	PRO	VAL	engineered mutation	UNP P0DTC2
B	1209	GLY	-	expression tag	UNP P0DTC2
B	1210	SER	-	expression tag	UNP P0DTC2
B	1211	GLY	-	expression tag	UNP P0DTC2
B	1212	TYR	-	expression tag	UNP P0DTC2
B	1213	ILE	-	expression tag	UNP P0DTC2
B	1214	PRO	-	expression tag	UNP P0DTC2
B	1215	GLU	-	expression tag	UNP P0DTC2
B	1216	ALA	-	expression tag	UNP P0DTC2
B	1217	PRO	-	expression tag	UNP P0DTC2
B	1218	ARG	-	expression tag	UNP P0DTC2

Continued on next page...

For Manuscript Review

Continued from previous page...

Chain	Residue	Modelled	Actual	Comment	Reference
B	1219	ASP	-	expression tag	UNP P0DTC2
B	1220	GLY	-	expression tag	UNP P0DTC2
B	1221	GLN	-	expression tag	UNP P0DTC2
B	1222	ALA	-	expression tag	UNP P0DTC2
B	1223	TYR	-	expression tag	UNP P0DTC2
B	1224	VAL	-	expression tag	UNP P0DTC2
B	1225	ARG	-	expression tag	UNP P0DTC2
B	1226	LYS	-	expression tag	UNP P0DTC2
B	1227	ASP	-	expression tag	UNP P0DTC2
B	1228	GLY	-	expression tag	UNP P0DTC2
B	1229	GLU	-	expression tag	UNP P0DTC2
B	1230	TRP	-	expression tag	UNP P0DTC2
B	1231	VAL	-	expression tag	UNP P0DTC2
B	1232	LEU	-	expression tag	UNP P0DTC2
B	1233	LEU	-	expression tag	UNP P0DTC2
B	1234	SER	-	expression tag	UNP P0DTC2
B	1235	THR	-	expression tag	UNP P0DTC2
B	1236	PHE	-	expression tag	UNP P0DTC2
B	1237	LEU	-	expression tag	UNP P0DTC2
B	1238	GLY	-	expression tag	UNP P0DTC2
B	1239	ARG	-	expression tag	UNP P0DTC2
B	1240	SER	-	expression tag	UNP P0DTC2
B	1241	LEU	-	expression tag	UNP P0DTC2
B	1242	GLU	-	expression tag	UNP P0DTC2
B	1243	VAL	-	expression tag	UNP P0DTC2
B	1244	LEU	-	expression tag	UNP P0DTC2
B	1245	PHE	-	expression tag	UNP P0DTC2
B	1246	GLN	-	expression tag	UNP P0DTC2
B	1247	GLY	-	expression tag	UNP P0DTC2
B	1248	PRO	-	expression tag	UNP P0DTC2
B	1249	GLY	-	expression tag	UNP P0DTC2
B	1250	HIS	-	expression tag	UNP P0DTC2
B	1251	HIS	-	expression tag	UNP P0DTC2
B	1252	HIS	-	expression tag	UNP P0DTC2
B	1253	HIS	-	expression tag	UNP P0DTC2
B	1254	HIS	-	expression tag	UNP P0DTC2
B	1255	HIS	-	expression tag	UNP P0DTC2
B	1256	HIS	-	expression tag	UNP P0DTC2
B	1257	HIS	-	expression tag	UNP P0DTC2
B	1258	SER	-	expression tag	UNP P0DTC2
B	1259	ALA	-	expression tag	UNP P0DTC2
B	1260	TRP	-	expression tag	UNP P0DTC2

Continued on next page...

For Manuscript Review

Continued from previous page...

Chain	Residue	Modelled	Actual	Comment	Reference
B	1261	SER	-	expression tag	UNP P0DTC2
B	1262	HIS	-	expression tag	UNP P0DTC2
B	1263	PRO	-	expression tag	UNP P0DTC2
B	1264	GLN	-	expression tag	UNP P0DTC2
B	1265	PHE	-	expression tag	UNP P0DTC2
B	1266	GLU	-	expression tag	UNP P0DTC2
B	1267	LYS	-	expression tag	UNP P0DTC2
B	1268	GLY	-	expression tag	UNP P0DTC2
B	1269	GLY	-	expression tag	UNP P0DTC2
B	1270	GLY	-	expression tag	UNP P0DTC2
B	1271	SER	-	expression tag	UNP P0DTC2
B	1272	GLY	-	expression tag	UNP P0DTC2
B	1273	GLY	-	expression tag	UNP P0DTC2
B	1274	GLY	-	expression tag	UNP P0DTC2
B	1275	GLY	-	expression tag	UNP P0DTC2
B	1276	SER	-	expression tag	UNP P0DTC2
B	1277	GLY	-	expression tag	UNP P0DTC2
B	1278	GLY	-	expression tag	UNP P0DTC2
B	1279	SER	-	expression tag	UNP P0DTC2
B	1280	ALA	-	expression tag	UNP P0DTC2
B	1281	TRP	-	expression tag	UNP P0DTC2
B	1282	SER	-	expression tag	UNP P0DTC2
B	1283	HIS	-	expression tag	UNP P0DTC2
B	1284	PRO	-	expression tag	UNP P0DTC2
B	1285	GLN	-	expression tag	UNP P0DTC2
B	1286	PHE	-	expression tag	UNP P0DTC2
B	1287	GLU	-	expression tag	UNP P0DTC2
B	1288	LYS	-	expression tag	UNP P0DTC2
F	682	GLY	ARG	engineered mutation	UNP P0DTC2
F	683	SER	ARG	engineered mutation	UNP P0DTC2
F	685	SER	ARG	engineered mutation	UNP P0DTC2
F	817	PRO	PHE	engineered mutation	UNP P0DTC2
F	892	PRO	ALA	engineered mutation	UNP P0DTC2
F	899	PRO	ALA	engineered mutation	UNP P0DTC2
F	942	PRO	ALA	engineered mutation	UNP P0DTC2
F	986	PRO	LYS	engineered mutation	UNP P0DTC2
F	987	PRO	VAL	engineered mutation	UNP P0DTC2
F	1209	GLY	-	expression tag	UNP P0DTC2
F	1210	SER	-	expression tag	UNP P0DTC2
F	1211	GLY	-	expression tag	UNP P0DTC2
F	1212	TYR	-	expression tag	UNP P0DTC2
F	1213	ILE	-	expression tag	UNP P0DTC2

Continued on next page...

For Manuscript Review

Continued from previous page...

Chain	Residue	Modelled	Actual	Comment	Reference
F	1214	PRO	-	expression tag	UNP P0DTC2
F	1215	GLU	-	expression tag	UNP P0DTC2
F	1216	ALA	-	expression tag	UNP P0DTC2
F	1217	PRO	-	expression tag	UNP P0DTC2
F	1218	ARG	-	expression tag	UNP P0DTC2
F	1219	ASP	-	expression tag	UNP P0DTC2
F	1220	GLY	-	expression tag	UNP P0DTC2
F	1221	GLN	-	expression tag	UNP P0DTC2
F	1222	ALA	-	expression tag	UNP P0DTC2
F	1223	TYR	-	expression tag	UNP P0DTC2
F	1224	VAL	-	expression tag	UNP P0DTC2
F	1225	ARG	-	expression tag	UNP P0DTC2
F	1226	LYS	-	expression tag	UNP P0DTC2
F	1227	ASP	-	expression tag	UNP P0DTC2
F	1228	GLY	-	expression tag	UNP P0DTC2
F	1229	GLU	-	expression tag	UNP P0DTC2
F	1230	TRP	-	expression tag	UNP P0DTC2
F	1231	VAL	-	expression tag	UNP P0DTC2
F	1232	LEU	-	expression tag	UNP P0DTC2
F	1233	LEU	-	expression tag	UNP P0DTC2
F	1234	SER	-	expression tag	UNP P0DTC2
F	1235	THR	-	expression tag	UNP P0DTC2
F	1236	PHE	-	expression tag	UNP P0DTC2
F	1237	LEU	-	expression tag	UNP P0DTC2
F	1238	GLY	-	expression tag	UNP P0DTC2
F	1239	ARG	-	expression tag	UNP P0DTC2
F	1240	SER	-	expression tag	UNP P0DTC2
F	1241	LEU	-	expression tag	UNP P0DTC2
F	1242	GLU	-	expression tag	UNP P0DTC2
F	1243	VAL	-	expression tag	UNP P0DTC2
F	1244	LEU	-	expression tag	UNP P0DTC2
F	1245	PHE	-	expression tag	UNP P0DTC2
F	1246	GLN	-	expression tag	UNP P0DTC2
F	1247	GLY	-	expression tag	UNP P0DTC2
F	1248	PRO	-	expression tag	UNP P0DTC2
F	1249	GLY	-	expression tag	UNP P0DTC2
F	1250	HIS	-	expression tag	UNP P0DTC2
F	1251	HIS	-	expression tag	UNP P0DTC2
F	1252	HIS	-	expression tag	UNP P0DTC2
F	1253	HIS	-	expression tag	UNP P0DTC2
F	1254	HIS	-	expression tag	UNP P0DTC2
F	1255	HIS	-	expression tag	UNP P0DTC2

Continued on next page...

For Manuscript Review

Continued from previous page...

Chain	Residue	Modelled	Actual	Comment	Reference
F	1256	HIS	-	expression tag	UNP P0DTC2
F	1257	HIS	-	expression tag	UNP P0DTC2
F	1258	SER	-	expression tag	UNP P0DTC2
F	1259	ALA	-	expression tag	UNP P0DTC2
F	1260	TRP	-	expression tag	UNP P0DTC2
F	1261	SER	-	expression tag	UNP P0DTC2
F	1262	HIS	-	expression tag	UNP P0DTC2
F	1263	PRO	-	expression tag	UNP P0DTC2
F	1264	GLN	-	expression tag	UNP P0DTC2
F	1265	PHE	-	expression tag	UNP P0DTC2
F	1266	GLU	-	expression tag	UNP P0DTC2
F	1267	LYS	-	expression tag	UNP P0DTC2
F	1268	GLY	-	expression tag	UNP P0DTC2
F	1269	GLY	-	expression tag	UNP P0DTC2
F	1270	GLY	-	expression tag	UNP P0DTC2
F	1271	SER	-	expression tag	UNP P0DTC2
F	1272	GLY	-	expression tag	UNP P0DTC2
F	1273	GLY	-	expression tag	UNP P0DTC2
F	1274	GLY	-	expression tag	UNP P0DTC2
F	1275	GLY	-	expression tag	UNP P0DTC2
F	1276	SER	-	expression tag	UNP P0DTC2
F	1277	GLY	-	expression tag	UNP P0DTC2
F	1278	GLY	-	expression tag	UNP P0DTC2
F	1279	SER	-	expression tag	UNP P0DTC2
F	1280	ALA	-	expression tag	UNP P0DTC2
F	1281	TRP	-	expression tag	UNP P0DTC2
F	1282	SER	-	expression tag	UNP P0DTC2
F	1283	HIS	-	expression tag	UNP P0DTC2
F	1284	PRO	-	expression tag	UNP P0DTC2
F	1285	GLN	-	expression tag	UNP P0DTC2
F	1286	PHE	-	expression tag	UNP P0DTC2
F	1287	GLU	-	expression tag	UNP P0DTC2
F	1288	LYS	-	expression tag	UNP P0DTC2

- Molecule 2 is a protein called S2L20 Fab Heavy Chain Variable.

Mol	Chain	Residues	Atoms					AltConf	Trace
			Total	C	N	O	S		
2	C	121	827	528	143	153	3	0	0
2	D	121	827	528	143	153	3	0	0
2	G	121	827	528	143	153	3	0	0

For Manuscript Review

- Molecule 3 is a protein called S2L20 Fab Light Chain Variable Region.

Mol	Chain	Residues	Atoms					AltConf	Trace
			Total	C	N	O	S		
3	L	107	543	327	107	107	2	0	0
3	E	107	543	327	107	107	2	0	0
3	H	107	543	327	107	107	2	0	0

- Molecule 4 is an oligosaccharide called 2-acetamido-2-deoxy-beta-D-glucopyranose-(1-4)-2-acetamido-2-deoxy-beta-D-glucopyranose.

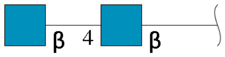

Mol	Chain	Residues	Atoms				AltConf	Trace
			Total	C	N	O		
4	I	2	28	16	2	10	0	0
4	K	2	28	16	2	10	0	0
4	N	2	28	16	2	10	0	0

- Molecule 5 is an oligosaccharide called beta-D-mannopyranose-(1-4)-2-acetamido-2-deoxy-beta-D-glucopyranose-(1-4)-2-acetamido-2-deoxy-beta-D-glucopyranose.

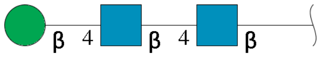

Mol	Chain	Residues	Atoms				AltConf	Trace
			Total	C	N	O		
5	J	3	39	22	2	15	0	0
5	M	3	39	22	2	15	0	0
5	O	3	39	22	2	15	0	0

- Molecule 6 is 2-acetamido-2-deoxy-beta-D-glucopyranose (three-letter code: NAG) (formula: $C_8H_{15}NO_6$).

For Manuscript Review

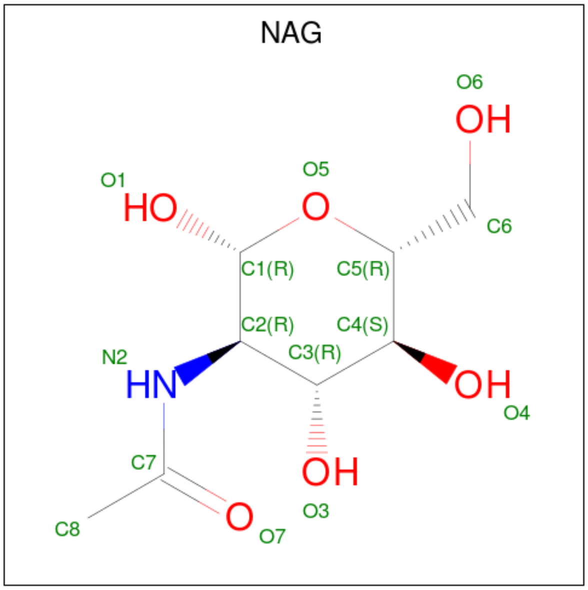

Mol	Chain	Residues	Atoms				AltConf
6	A	1	Total 14	C 8	N 1	O 5	0
6	A	1	Total 14	C 8	N 1	O 5	0
6	A	1	Total 14	C 8	N 1	O 5	0
6	A	1	Total 14	C 8	N 1	O 5	0
6	A	1	Total 14	C 8	N 1	O 5	0
6	A	1	Total 14	C 8	N 1	O 5	0
6	A	1	Total 14	C 8	N 1	O 5	0
6	A	1	Total 14	C 8	N 1	O 5	0
6	A	1	Total 14	C 8	N 1	O 5	0
6	A	1	Total 14	C 8	N 1	O 5	0
6	A	1	Total 14	C 8	N 1	O 5	0
6	A	1	Total 14	C 8	N 1	O 5	0
6	A	1	Total 14	C 8	N 1	O 5	0
6	A	1	Total 14	C 8	N 1	O 5	0

Continued on next page...

For Manuscript Review

Continued from previous page...

Mol	Chain	Residues	Atoms	AltConf
6	A	1	Total C N O<br>14 8 1 5	0
6	B	1	Total C N O<br>14 8 1 5	0
6	B	1	Total C N O<br>14 8 1 5	0
6	B	1	Total C N O<br>14 8 1 5	0
6	B	1	Total C N O<br>14 8 1 5	0
6	B	1	Total C N O<br>14 8 1 5	0
6	B	1	Total C N O<br>14 8 1 5	0
6	B	1	Total C N O<br>14 8 1 5	0
6	B	1	Total C N O<br>14 8 1 5	0
6	B	1	Total C N O<br>14 8 1 5	0
6	B	1	Total C N O<br>14 8 1 5	0
6	B	1	Total C N O<br>14 8 1 5	0
6	B	1	Total C N O<br>14 8 1 5	0
6	B	1	Total C N O<br>14 8 1 5	0
6	B	1	Total C N O<br>14 8 1 5	0
6	B	1	Total C N O<br>14 8 1 5	0
6	F	1	Total C N O<br>14 8 1 5	0
6	F	1	Total C N O<br>14 8 1 5	0
6	F	1	Total C N O<br>14 8 1 5	0
6	F	1	Total C N O<br>14 8 1 5	0
6	F	1	Total C N O<br>14 8 1 5	0

Continued on next page...

For Manuscript Review

Continued from previous page...

Mol	Chain	Residues	Atoms	AltConf
6	F	1	Total C N O<br>14 8 1 5	0
6	F	1	Total C N O<br>14 8 1 5	0
6	F	1	Total C N O<br>14 8 1 5	0
6	F	1	Total C N O<br>14 8 1 5	0
6	F	1	Total C N O<br>14 8 1 5	0
6	F	1	Total C N O<br>14 8 1 5	0
6	F	1	Total C N O<br>14 8 1 5	0
6	F	1	Total C N O<br>14 8 1 5	0
6	F	1	Total C N O<br>14 8 1 5	0
6	F	1	Total C N O<br>14 8 1 5	0

For Manuscript Review

3 Residue-property plots (i)

These plots are drawn for all protein, RNA, DNA and oligosaccharide chains in the entry. The first graphic for a chain summarises the proportions of the various outlier classes displayed in the second graphic. The second graphic shows the sequence view annotated by issues in geometry and atom inclusion in map density. Residues are color-coded according to the number of geometric quality criteria for which they contain at least one outlier: green = 0, yellow = 1, orange = 2 and red = 3 or more. A red diamond above a residue indicates a poor fit to the EM map for this residue (all-atom inclusion < 40%). Stretches of 2 or more consecutive residues without any outlier are shown as a green connector. Residues present in the sample, but not in the model, are shown in grey.

- Molecule 1: Spike glycoprotein

Chain A:

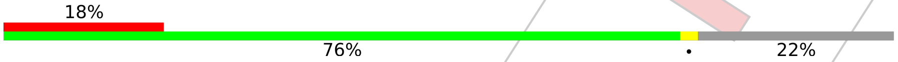

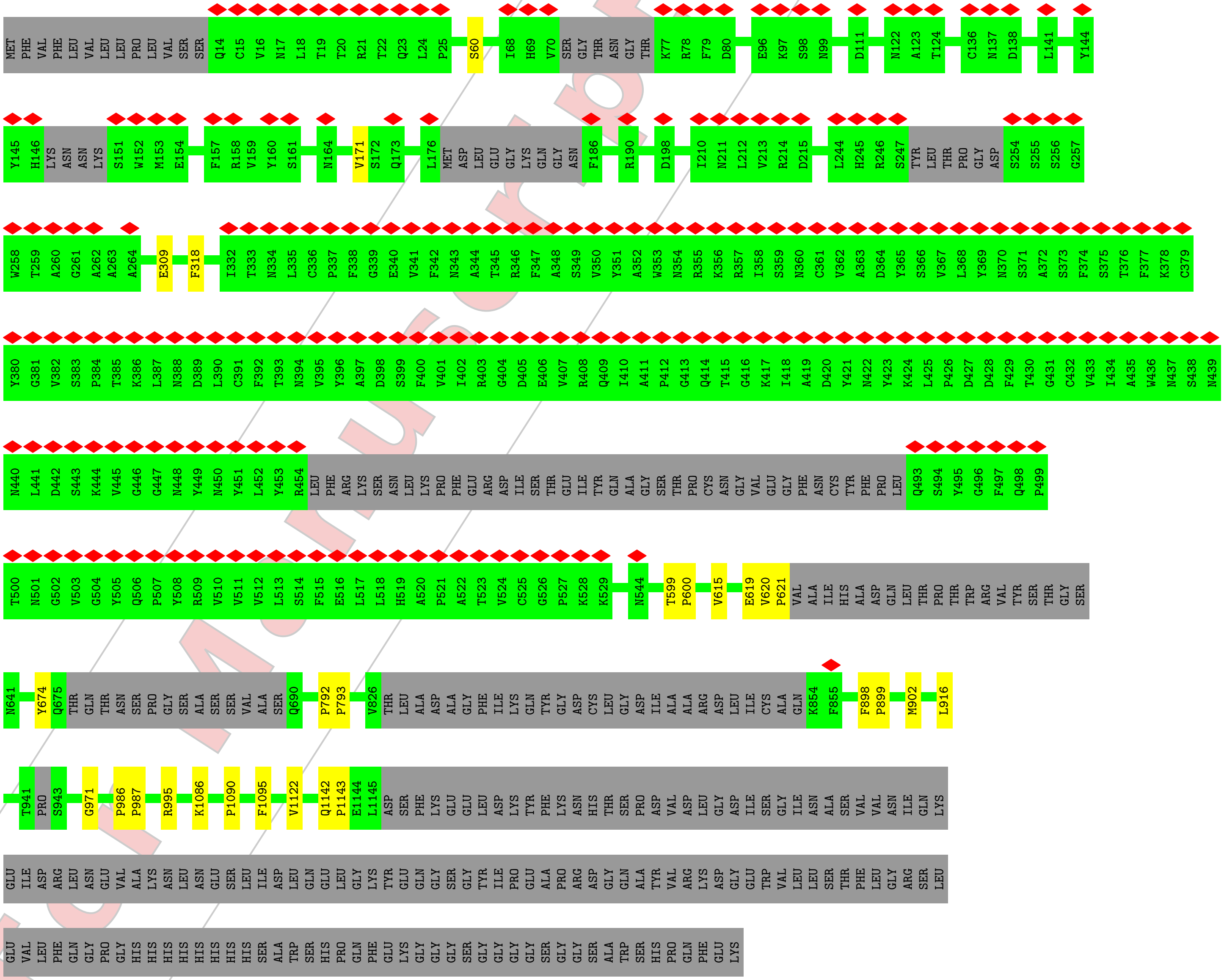

For Manuscript Review

- Molecule 1: Spike glycoprotein

Chain B:

18%

76% • 22%

MET PHE VAL PHE LEU VAL LEU LEU PRO LEU VAL SER SER Q14 C15 V16 N17 L18 T19 T20 R21 T22 Q23 L24 P25 S60 I68 H69 V70 SER GLY THR ASN GLY THR K77 R78 F79 D80 E96 K97 S98 N99 D111 N122 A123 T124 C136 N137 D138 L141 Y144

Y145 H146 LYS ASN ASN LYS S151 W152 M153 E154 F157 R158 V159 Y160 S161 N164 V171 S172 Q173 L176 MET ASP LEU GLU GLY LYS GLN GLY ASN F186 K187 N188 L189 R190 D198 I210 N211 L212 V213 R214 D215 L244 H245 R246 S247 TYR LEU THR PRO GLY ASP S254 S255

S256 G257 W258 T259 A260 G261 A262 A263 A264 E309 F318 I332 T333 N334 L335 C336 P337 F338 G339 E340 V341 F342 N343 A344 T345 R346 F347 A348 S349 V350 Y351 A352 W353 N354 R355 K356 R357 I358 S359 N360 C361 V362 A363 D364 Y365 S366 V367 L368 Y369 N370 S371 A372 S373 F374 S375 T376 F377

K378 C379 Y380 G381 V382 S383 P384 T385 K386 L387 N388 D389 L390 C391 F392 T393 N394 V395 Y396 A397 D398 S399 F400 V401 I402 R403 G404 D405 E406 V407 R408 Q409 I410 A411 P412 G413 Q414 T415 G416 K417 I418 A419 D420 Y421 N422 Y423 K424 L425 P426 D427 D428 F429 T430 G431 C432 V433 I434 A435 W436 N437

S438 N439 N440 L441 D442 S443 K444 V445 G446 G447 N448 Y449 N450 Y451 L452 Y453 R454 LEU PHE ARG LYS SER ASN LEU LYS PRO PHE GLU ARG ASP ILE SER THR GLU ILE TYR GLN ALA GLY SER THR PRO CYS ASN GLY VAL GLU GLY PHE ASN CYS TYR PHE PRO LEU Q493 S494 Y495 G496 F497

Q498 P499 T500 N501 G502 V503 G504 Y505 Q506 P507 Y508 R509 V510 V511 V512 L513 S514 F515 E516 L517 L518 H519 A520 P521 A522 T523 V524 C525 G526 P527 K528 K529 N544 T599 V615 E619 V620 P621 VAL ALA ILE HIS ALA ASP GLN LEU THR PRO THR TRP ARG VAL TYR SER THR GLY

SER N641 Q675 THR GLN THR ASN SER PRO GLY SER ALA SER SER VAL ALA SER Q690 I714 P715 V826 THR LEU ALA ASP ALA GLY PHE ILE LYS GLN TYR GLY ASP CYS LEU GLY ASP ILE ALA ALA ARG ASP LEU ILE CYS ALA GLN K854 F855 F898 P899 M902 L916

T941 PRO S943 G971 P986 P987 R995 K1086 P1090 F1095 V1122 Q1142 P1143 E1144 L1145 ASP SER PHE LYS GLU GLU LEU ASP LYS TYR PHE LYS ASN HIS THR SER PRO ASP VAL ASP LEU GLY ASP ILE SER GLY ILE ASN ALA SER VAL VAL ASN ILE GLN LYS

GLU ILE ASP ARG LEU ASN GLU VAL ALA LYS ASN LEU ASN GLU SER LEU ILE ASP LEU GLN GLU LEU GLY LYS TYR GLU GLN GLY SER GLY TYR ILE PRO GLU ALA PRO ARG ASP GLY GLN ALA TYR VAL ARG LYS ASP GLY GLU TRP VAL LEU LEU SER THR PHE LEU GLY ARG SER LEU

GLU VAL LEU PHE GLN GLY PRO GLY HIS HIS HIS HIS HIS HIS SER ALA TRP SER HIS PRO GLN PHE GLU LYS GLY GLY GLY SER GLY GLY GLY SER GLY GLY SER ALA TRP SER HIS PRO GLN PHE GLU LYS

- Molecule 1: Spike glycoprotein

Chain F:

18%

76% • 22%

MET PHE VAL PHE LEU VAL LEU LEU PRO LEU VAL SER SER Q14 C15 V16 N17 L18 T19 T20 R21 T22 Q23 L24 P25 S60 I68 H69 V70 SER GLY THR ASN GLY THR K77 R78 F79 D80 E96 K97 S98 N99 D111 N122 A123 T124 C136 N137 D138 L141 Y144

Y145 H146 LYS ASN ASN LYS S151 W152 M153 E154 F157 R158 V159 Y160 S161 N164 V171 S172 Q173 L176 MET ASP LEU GLU GLY LYS GLN GLY ASN F186 K187 N188 L189 R190 D198 I210 N211 L212 V213 R214 D215 L244 H245 R246 S247 TYR LEU THR PRO GLY ASP S254 S255

For Manuscript Review

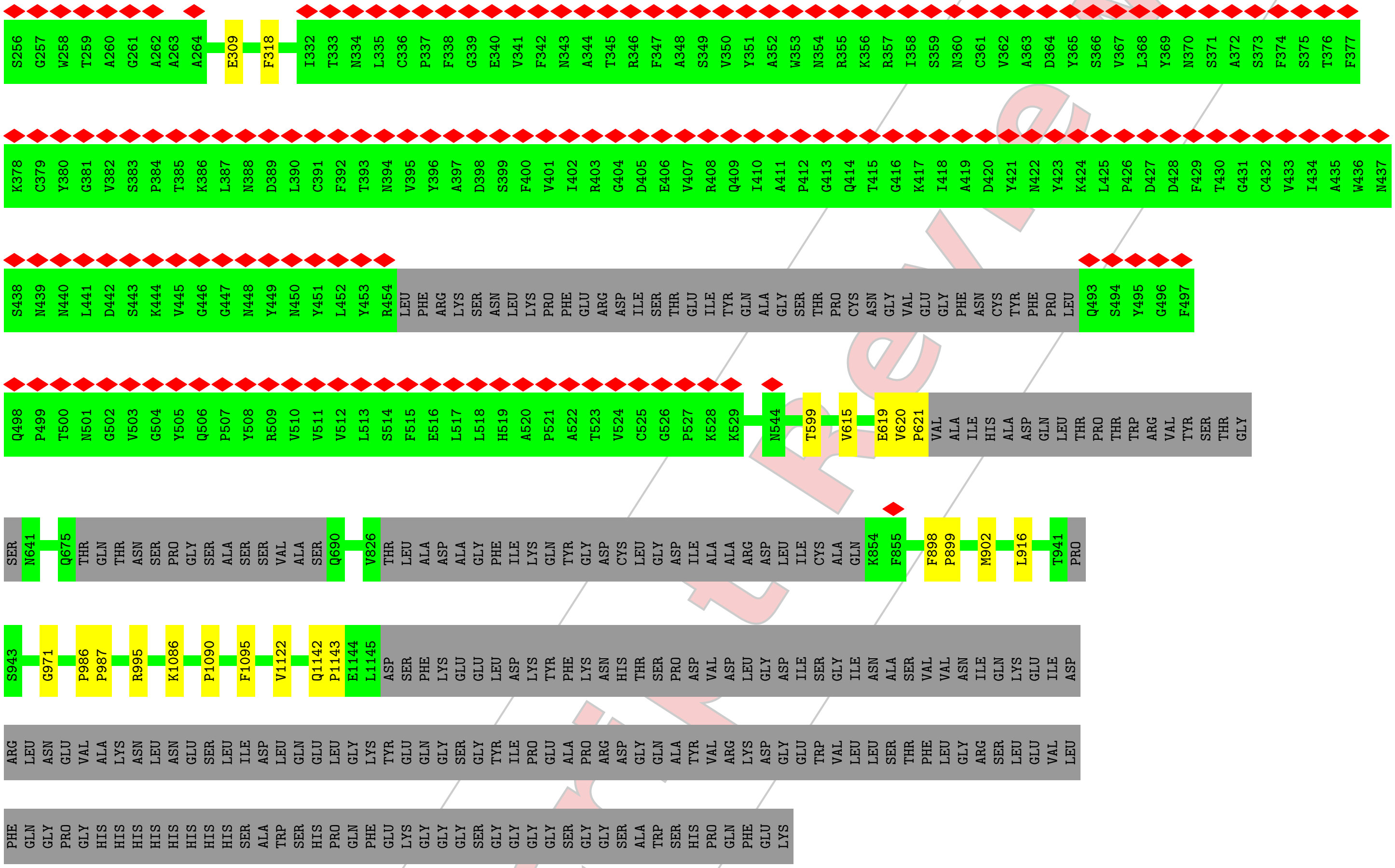

- Molecule 2: S2L20 Fab Heavy Chain Variable

Chain C:

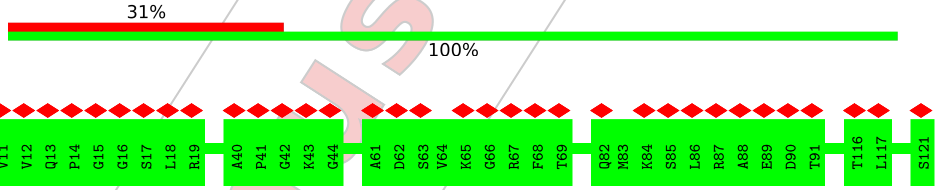

- Molecule 2: S2L20 Fab Heavy Chain Variable

Chain D:

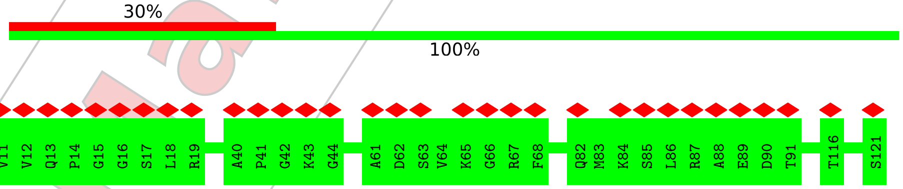

- Molecule 2: S2L20 Fab Heavy Chain Variable

Chain G:

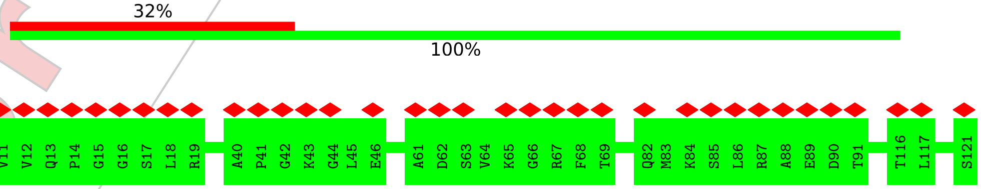

- Molecule 3: S2L20 Fab Light Chain Variable Region

For Manuscript Review

Chain L:

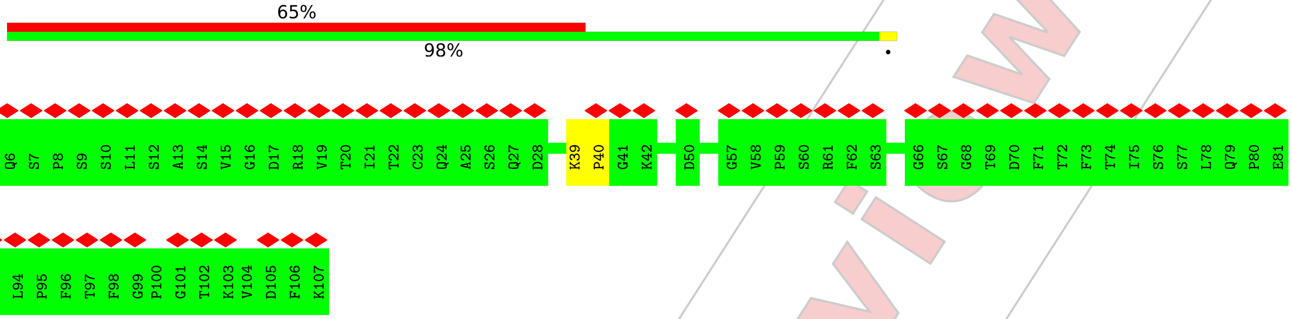

• Molecule 3: S2L20 Fab Light Chain Variable Region

Chain E:

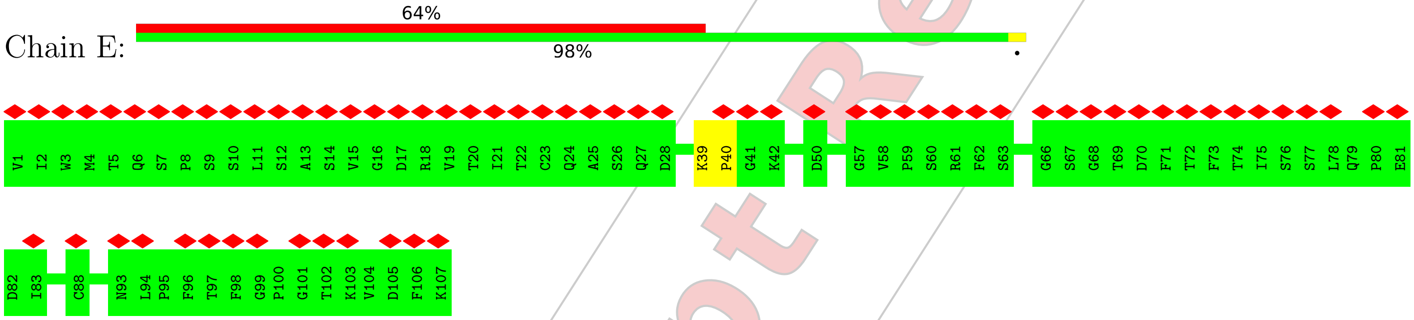

• Molecule 3: S2L20 Fab Light Chain Variable Region

Chain H:

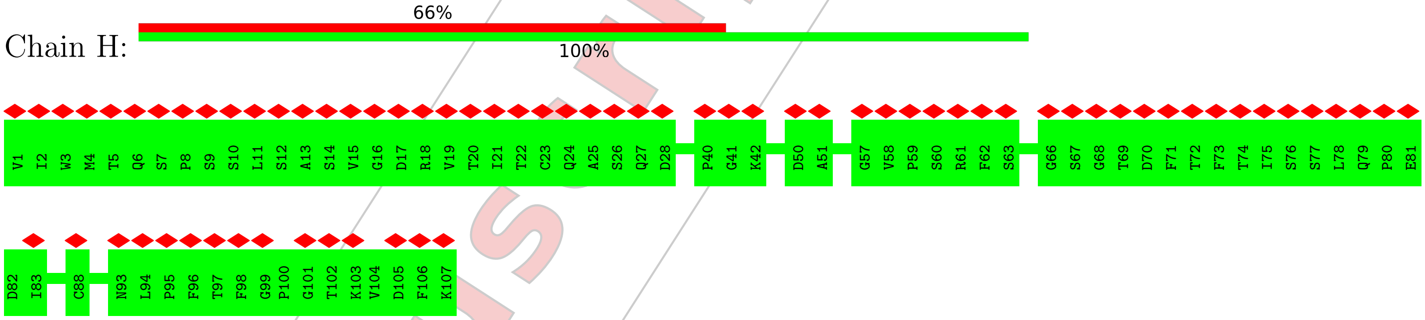

• Molecule 4: 2-acetamido-2-deoxy-beta-D-glucopyranose-(1-4)-2-acetamido-2-deoxy-beta-D-glucopyranose

Chain I:

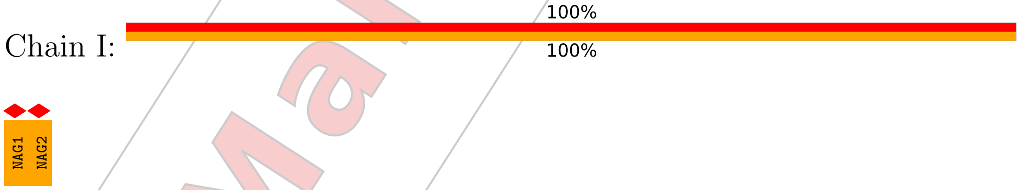

• Molecule 4: 2-acetamido-2-deoxy-beta-D-glucopyranose-(1-4)-2-acetamido-2-deoxy-beta-D-glucopyranose

Chain K:

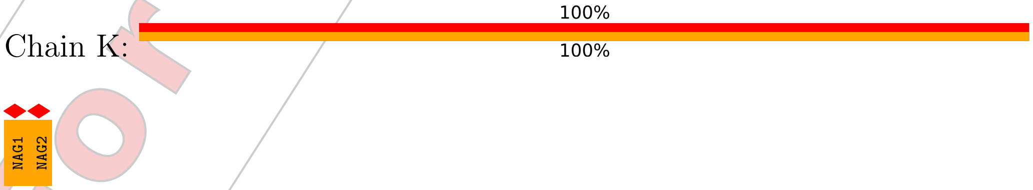

For Manuscript Review

• Molecule 4: 2-acetamido-2-deoxy-beta-D-glucopyranose-(1-4)-2-acetamido-2-deoxy-beta-D-gluc opyranose

Chain N:
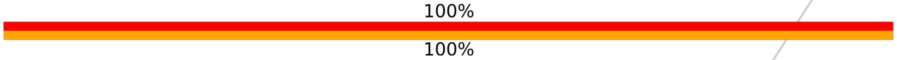

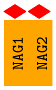

• Molecule 5: beta-D-mannopyranose-(1-4)-2-acetamido-2-deoxy-beta-D-glucopyranose-(1-4)-2-ac etamido-2-deoxy-beta-D-glucopyranose

Chain J:
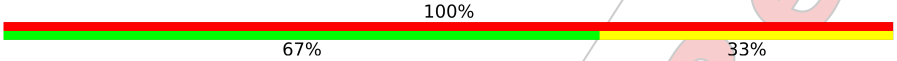

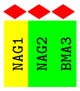

• Molecule 5: beta-D-mannopyranose-(1-4)-2-acetamido-2-deoxy-beta-D-glucopyranose-(1-4)-2-ac etamido-2-deoxy-beta-D-glucopyranose

Chain M:
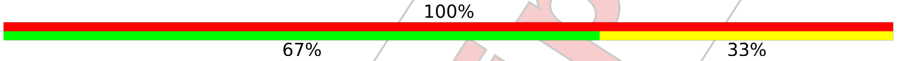

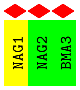

• Molecule 5: beta-D-mannopyranose-(1-4)-2-acetamido-2-deoxy-beta-D-glucopyranose-(1-4)-2-ac etamido-2-deoxy-beta-D-glucopyranose

Chain O:
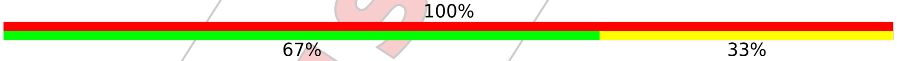

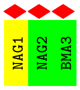

For Manuscript Review

4 Experimental information ⓘ

Property	Value	Source
EM reconstruction method	SINGLE PARTICLE	Depositor
Imposed symmetry	POINT, Not provided	
Number of particles used	165733	Depositor
Resolution determination method	FSC 0.143 CUT-OFF	Depositor
CTF correction method	PHASE FLIPPING AND AMPLITUDE CORRECTION	Depositor
Microscope	FEI TITAN KRIOS	Depositor
Voltage (kV)	300	Depositor
Electron dose $(e^-/\text{Å}^2)$	63	Depositor
Minimum defocus (nm)	2000	Depositor
Maximum defocus (nm)	20000	Depositor
Magnification	Not provided	
Image detector	GATAN K3 (6k x 4k)	Depositor
Maximum map value	10.265	Depositor
Minimum map value	-6.278	Depositor
Average map value	0.003	Depositor
Map value standard deviation	0.086	Depositor
Recommended contour level	0.6	Depositor
Map size (Å)	431.616, 431.616, 431.616	wwPDB
Map dimensions	512, 512, 512	wwPDB
Map angles (°)	90.0, 90.0, 90.0	wwPDB
Pixel spacing (Å)	0.843, 0.843, 0.843	Depositor

For Manuscript Review

5 Model quality (i)

5.1 Standard geometry (i)

Bond lengths and bond angles in the following residue types are not validated in this section: NAG, BMA

The Z score for a bond length (or angle) is the number of standard deviations the observed value is removed from the expected value. A bond length (or angle) with $|Z| > 5$ is considered an outlier worth inspection. RMSZ is the root-mean-square of all Z scores of the bond lengths (or angles).

Mol	Chain	Bond lengths		Bond angles	
		RMSZ	#|Z| >5	RMSZ	#|Z| >5
1	A	0.55	3/7105 (0.0%)	0.51	0/9755
1	B	0.55	3/7105 (0.0%)	0.51	0/9755
1	F	0.55	3/7105 (0.0%)	0.51	0/9755
2	C	0.52	0/849	0.58	0/1162
2	D	0.52	0/849	0.58	0/1162
2	G	0.51	0/849	0.58	0/1162
3	E	0.35	0/549	0.46	0/767
3	H	0.36	0/549	0.46	0/767
3	L	0.35	0/549	0.46	0/767
All	All	0.53	9/25509 (0.0%)	0.51	0/35052

All (9) bond length outliers are listed below:

Mol	Chain	Res	Type	Atoms	Z	Observed(Å)	Ideal(Å)
1	B	619	GLU	CD-OE1	-5.78	1.19	1.25
1	F	619	GLU	CD-OE1	-5.75	1.19	1.25
1	A	619	GLU	CD-OE1	-5.74	1.19	1.25
1	B	309	GLU	CD-OE2	-5.40	1.19	1.25
1	F	309	GLU	CD-OE2	-5.38	1.19	1.25
1	A	309	GLU	CD-OE2	-5.37	1.19	1.25
1	B	309	GLU	CD-OE1	-5.10	1.20	1.25
1	F	309	GLU	CD-OE1	-5.10	1.20	1.25
1	A	309	GLU	CD-OE1	-5.10	1.20	1.25

There are no bond angle outliers.

There are no chirality outliers.

There are no planarity outliers.

For Manuscript Review

5.2 Too-close contacts (i)

In the following table, the Non-H and H(model) columns list the number of non-hydrogen atoms and hydrogen atoms in the chain respectively. The H(added) column lists the number of hydrogen atoms added and optimized by MolProbity. The Clashes column lists the number of clashes within the asymmetric unit, whereas Symm-Clashes lists symmetry-related clashes.

Mol	Chain	Non-H	H(model)	H(added)	Clashes	Symm-Clashes
1	A	6968	0	6093	16	0
1	B	6968	0	6093	15	0
1	F	6968	0	6093	14	0
2	C	827	0	662	0	0
2	D	827	0	662	0	0
2	G	827	0	662	0	0
3	E	543	0	278	1	0
3	H	543	0	278	0	0
3	L	543	0	278	1	0
4	I	28	0	25	2	0
4	K	28	0	25	2	0
4	N	28	0	25	2	0
5	J	39	0	34	0	0
5	M	39	0	34	0	0
5	O	39	0	34	0	0
6	A	210	0	193	5	0
6	B	210	0	193	6	0
6	F	210	0	193	5	0
All	All	25845	0	21855	63	0

The all-atom clashscore is defined as the number of clashes found per 1000 atoms (including hydrogen atoms). The all-atom clashscore for this structure is 1.

All (63) close contacts within the same asymmetric unit are listed below, sorted by their clash magnitude.

Atom-1	Atom-2	Interatomic distance (Å)	Clash overlap (Å)
6:A:1308:NAG:O4	6:A:1309:NAG:C1	1.64	1.46
6:F:1308:NAG:O4	6:F:1309:NAG:C1	1.64	1.46
6:B:1310:NAG:O4	6:B:1311:NAG:C1	1.65	1.45
6:F:1310:NAG:O4	6:F:1311:NAG:C1	1.64	1.45
6:B:1308:NAG:O4	6:B:1309:NAG:C1	1.64	1.43
6:A:1310:NAG:O4	6:A:1311:NAG:C1	1.65	1.41
6:F:1308:NAG:C4	6:F:1309:NAG:C1	2.49	0.91
6:A:1308:NAG:C4	6:A:1309:NAG:C1	2.49	0.90
6:A:1310:NAG:C4	6:A:1311:NAG:C1	2.50	0.90

Continued on next page...

For Manuscript Review

Continued from previous page...

Atom-1	Atom-2	Interatomic distance (Å)	Clash overlap (Å)
6:F:1310:NAG:C4	6:F:1311:NAG:C1	2.50	0.90
6:B:1310:NAG:C4	6:B:1311:NAG:C1	2.50	0.89
6:B:1308:NAG:C4	6:B:1309:NAG:C1	2.49	0.89
1:B:60:SER:HA	6:B:1301:NAG:H82	1.72	0.71
1:A:60:SER:HA	6:A:1301:NAG:H82	1.72	0.71
1:F:60:SER:HA	6:F:1301:NAG:H82	1.72	0.69
1:F:171:VAL:HG11	4:N:2:NAG:H82	1.85	0.58
1:A:171:VAL:HG11	4:I:2:NAG:H82	1.85	0.58
1:B:171:VAL:HG11	4:K:2:NAG:H82	1.85	0.57
1:A:1142:GLN:N	1:A:1143:PRO:CD	2.70	0.55
1:F:1142:GLN:N	1:F:1143:PRO:CD	2.70	0.54
1:B:1142:GLN:N	1:B:1143:PRO:CD	2.70	0.53
1:A:898:PHE:N	1:A:899:PRO:CD	2.75	0.50
1:F:898:PHE:N	1:F:899:PRO:CD	2.75	0.50
1:B:898:PHE:N	1:B:899:PRO:CD	2.75	0.49
1:B:971:GLY:HA3	1:B:995:ARG:HH11	1.78	0.48
1:A:902:MET:HG3	1:A:916:LEU:CD1	2.44	0.48
1:F:971:GLY:HA3	1:F:995:ARG:HH11	1.78	0.47
1:B:902:MET:HG3	1:B:916:LEU:CD1	2.44	0.47
1:A:971:GLY:HA3	1:A:995:ARG:HH11	1.78	0.47
1:F:902:MET:HG3	1:F:916:LEU:CD1	2.44	0.47
1:A:1086:LYS:HD2	1:A:1122:VAL:HG11	1.97	0.47
1:B:1086:LYS:HD2	1:B:1122:VAL:HG11	1.97	0.46
1:F:1086:LYS:HD2	1:F:1122:VAL:HG11	1.97	0.46
1:F:620:VAL:HB	1:F:621:PRO:HD3	1.99	0.45
1:F:986:PRO:N	1:F:987:PRO:CD	2.80	0.45
4:I:1:NAG:H61	4:I:2:NAG:C7	2.47	0.45
4:N:1:NAG:H61	4:N:2:NAG:C7	2.47	0.45
1:F:986:PRO:HB2	1:F:987:PRO:HD3	1.99	0.45
1:A:986:PRO:N	1:A:987:PRO:CD	2.80	0.45
1:B:620:VAL:HB	1:B:621:PRO:HD3	1.99	0.45
4:K:1:NAG:H61	4:K:2:NAG:C7	2.47	0.45
1:B:986:PRO:HB2	1:B:987:PRO:HD3	1.99	0.44
1:A:620:VAL:HB	1:A:621:PRO:HD3	1.99	0.44
1:B:986:PRO:N	1:B:987:PRO:CD	2.80	0.43
1:A:986:PRO:HB2	1:A:987:PRO:HD3	1.99	0.43
1:A:792:PRO:HA	1:A:793:PRO:HD3	1.88	0.43
1:B:898:PHE:HB3	1:B:899:PRO:HD3	2.01	0.43
1:F:620:VAL:N	1:F:621:PRO:HD2	2.34	0.42
1:B:620:VAL:N	1:B:621:PRO:HD2	2.34	0.42
1:A:620:VAL:N	1:A:621:PRO:HD2	2.34	0.42

Continued on next page...

For Manuscript Review

Continued from previous page...

Atom-1	Atom-2	Interatomic distance (Å)	Clash overlap (Å)
1:A:898:PHE:HB3	1:A:899:PRO:HD3	2.01	0.42
6:B:1308:NAG:H4	6:B:1309:NAG:C1	2.47	0.42
1:F:898:PHE:HB3	1:F:899:PRO:HD3	2.01	0.42
1:A:318:PHE:CZ	1:A:615:VAL:HB	2.55	0.41
1:A:1090:PRO:HD3	1:A:1095:PHE:CE2	2.55	0.41
1:B:318:PHE:CZ	1:B:615:VAL:HB	2.55	0.41
1:F:318:PHE:CZ	1:F:615:VAL:HB	2.55	0.41
3:L:39:LYS:HA	3:L:40:PRO:HD3	1.92	0.41
1:F:1090:PRO:HD3	1:F:1095:PHE:CE2	2.55	0.41
1:B:714:ILE:HA	1:B:715:PRO:HD3	1.89	0.40
1:B:1090:PRO:HD3	1:B:1095:PHE:CE2	2.55	0.40
3:E:39:LYS:HA	3:E:40:PRO:HD3	1.92	0.40
1:A:600:PRO:HB3	1:A:674:TYR:HB2	2.04	0.40

There are no symmetry-related clashes.

5.3 Torsion angles (i)

5.3.1 Protein backbone (i)

In the following table, the Percentiles column shows the percent Ramachandran outliers of the chain as a percentile score with respect to all PDB entries followed by that with respect to all EM entries.

The Analysed column shows the number of residues for which the backbone conformation was analysed, and the total number of residues.

Mol	Chain	Analysed	Favoured	Allowed	Outliers	Percentiles	
1	A	988/1288 (77%)	966 (98%)	22 (2%)	0	100	100
1	B	988/1288 (77%)	966 (98%)	22 (2%)	0	100	100
1	F	988/1288 (77%)	966 (98%)	22 (2%)	0	100	100
2	C	119/121 (98%)	117 (98%)	2 (2%)	0	100	100
2	D	119/121 (98%)	117 (98%)	2 (2%)	0	100	100
2	G	119/121 (98%)	117 (98%)	2 (2%)	0	100	100
3	E	105/107 (98%)	101 (96%)	4 (4%)	0	100	100
3	H	105/107 (98%)	101 (96%)	4 (4%)	0	100	100
3	L	105/107 (98%)	101 (96%)	4 (4%)	0	100	100
All	All	3636/4548 (80%)	3552 (98%)	84 (2%)	0	100	100

For Manuscript Review

There are no Ramachandran outliers to report.

5.3.2 Protein sidechains (i)

In the following table, the Percentiles column shows the percent sidechain outliers of the chain as a percentile score with respect to all PDB entries followed by that with respect to all EM entries.

The Analysed column shows the number of residues for which the sidechain conformation was analysed, and the total number of residues.

Mol	Chain	Analysed	Rotameric	Outliers	Percentiles	
1	A	634/1116 (57%)	633 (100%)	1 (0%)	93	98
1	B	634/1116 (57%)	633 (100%)	1 (0%)	93	98
1	F	634/1116 (57%)	633 (100%)	1 (0%)	93	98
2	C	60/98 (61%)	60 (100%)	0	100	100
2	D	60/98 (61%)	60 (100%)	0	100	100
2	G	60/98 (61%)	60 (100%)	0	100	100
3	E	9/94 (10%)	9 (100%)	0	100	100
3	H	9/94 (10%)	9 (100%)	0	100	100
3	L	9/94 (10%)	9 (100%)	0	100	100
All	All	2109/3924 (54%)	2106 (100%)	3 (0%)	93	98

All (3) residues with a non-rotameric sidechain are listed below:

Mol	Chain	Res	Type
1	A	599	THR
1	B	599	THR
1	F	599	THR

Sometimes sidechains can be flipped to improve hydrogen bonding and reduce clashes. All (6) such sidechains are listed below:

Mol	Chain	Res	Type
1	A	655	HIS
1	A	1011	GLN
1	B	655	HIS
1	B	1011	GLN
1	F	655	HIS
1	F	1011	GLN

For Manuscript Review

5.3.3 RNA (i)

There are no RNA molecules in this entry.

5.4 Non-standard residues in protein, DNA, RNA chains (i)

There are no non-standard protein/DNA/RNA residues in this entry.

5.5 Carbohydrates (i)

15 monosaccharides are modelled in this entry.

In the following table, the Counts columns list the number of bonds (or angles) for which Mogul statistics could be retrieved, the number of bonds (or angles) that are observed in the model and the number of bonds (or angles) that are defined in the Chemical Component Dictionary. The Link column lists molecule types, if any, to which the group is linked. The Z score for a bond length (or angle) is the number of standard deviations the observed value is removed from the expected value. A bond length (or angle) with $|Z| > 2$ is considered an outlier worth inspection. RMSZ is the root-mean-square of all Z scores of the bond lengths (or angles).

Mol	Type	Chain	Res	Link	Bond lengths			Bond angles		
					Counts	RMSZ	$\#\|Z\| > 2$	Counts	RMSZ	$\#\|Z\| > 2$
4	NAG	I	1	1,4	14,14,15	0.45	0	17,19,21	0.94	2 (11%)
4	NAG	I	2	4	14,14,15	0.42	0	17,19,21	0.63	1 (5%)
5	NAG	J	1	1,5	14,14,15	0.44	0	17,19,21	0.82	1 (5%)
5	NAG	J	2	5	14,14,15	0.44	0	17,19,21	0.69	0
5	BMA	J	3	5	11,11,12	0.39	0	15,15,17	0.55	0
4	NAG	K	1	1,4	14,14,15	0.45	0	17,19,21	0.94	2 (11%)
4	NAG	K	2	4	14,14,15	0.41	0	17,19,21	0.63	1 (5%)
5	NAG	M	1	1,5	14,14,15	0.44	0	17,19,21	0.82	1 (5%)
5	NAG	M	2	5	14,14,15	0.43	0	17,19,21	0.69	0
5	BMA	M	3	5	11,11,12	0.39	0	15,15,17	0.55	0
4	NAG	N	1	1,4	14,14,15	0.45	0	17,19,21	0.94	2 (11%)
4	NAG	N	2	4	14,14,15	0.42	0	17,19,21	0.64	1 (5%)
5	NAG	O	1	1,5	14,14,15	0.43	0	17,19,21	0.82	1 (5%)
5	NAG	O	2	5	14,14,15	0.43	0	17,19,21	0.68	0
5	BMA	O	3	5	11,11,12	0.39	0	15,15,17	0.55	0

In the following table, the Chirals column lists the number of chiral outliers, the number of chiral centers analysed, the number of these observed in the model and the number defined in the Chemical Component Dictionary. Similar counts are reported in the Torsion and Rings columns. '-' means no outliers of that kind were identified.

For Manuscript Review

Mol	Type	Chain	Res	Link	Chirals	Torsions	Rings
4	NAG	I	1	1,4	-	0/6/23/26	0/1/1/1
4	NAG	I	2	4	-	0/6/23/26	0/1/1/1
5	NAG	J	1	1,5	-	0/6/23/26	0/1/1/1
5	NAG	J	2	5	-	0/6/23/26	0/1/1/1
5	BMA	J	3	5	-	0/2/19/22	0/1/1/1
4	NAG	K	1	1,4	-	0/6/23/26	0/1/1/1
4	NAG	K	2	4	-	0/6/23/26	0/1/1/1
5	NAG	M	1	1,5	-	0/6/23/26	0/1/1/1
5	NAG	M	2	5	-	0/6/23/26	0/1/1/1
5	BMA	M	3	5	-	0/2/19/22	0/1/1/1
4	NAG	N	1	1,4	-	0/6/23/26	0/1/1/1
4	NAG	N	2	4	-	0/6/23/26	0/1/1/1
5	NAG	O	1	1,5	-	0/6/23/26	0/1/1/1
5	NAG	O	2	5	-	0/6/23/26	0/1/1/1
5	BMA	O	3	5	-	0/2/19/22	0/1/1/1

There are no bond length outliers.

All (12) bond angle outliers are listed below:

Mol	Chain	Res	Type	Atoms	Z	Observed(°)	Ideal(°)
4	I	1	NAG	C2-N2-C7	-2.58	119.24	122.90
4	N	1	NAG	C2-N2-C7	-2.56	119.25	122.90
4	K	1	NAG	C2-N2-C7	-2.56	119.26	122.90
5	J	1	NAG	C2-N2-C7	-2.32	119.60	122.90
5	O	1	NAG	C2-N2-C7	-2.29	119.64	122.90
5	M	1	NAG	C2-N2-C7	-2.29	119.64	122.90
4	K	2	NAG	C2-N2-C7	-2.04	119.99	122.90
4	N	2	NAG	C2-N2-C7	-2.04	120.00	122.90
4	I	2	NAG	C2-N2-C7	-2.03	120.02	122.90
4	N	1	NAG	O4-C4-C3	-2.03	105.67	110.35
4	K	1	NAG	O4-C4-C3	-2.02	105.67	110.35
4	I	1	NAG	O4-C4-C3	-2.02	105.67	110.35

There are no chirality outliers.

There are no torsion outliers.

There are no ring outliers.

6 monomers are involved in 6 short contacts:

Mol	Chain	Res	Type	Clashes	Symm-Clashes
4	K	1	NAG	1	0
4	N	2	NAG	2	0

Continued on next page...

For Manuscript Review

Continued from previous page...

Mol	Chain	Res	Type	Clashes	Symm-Clashes
4	K	2	NAG	2	0
4	I	2	NAG	2	0
4	I	1	NAG	1	0
4	N	1	NAG	1	0

The following is a two-dimensional graphical depiction of Mogul quality analysis of bond lengths, bond angles, torsion angles, and ring geometry for oligosaccharide.

Oligosaccharide Chain I

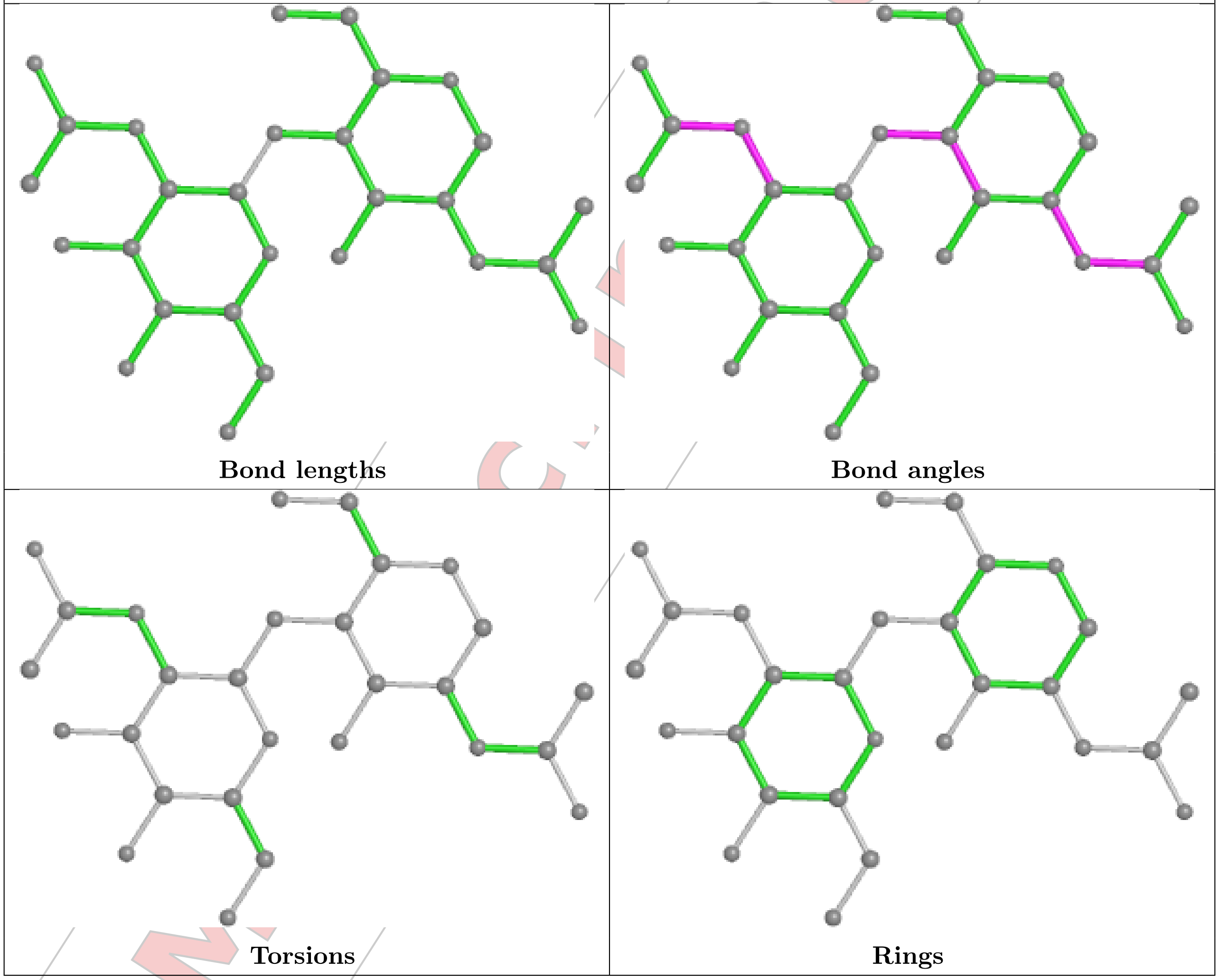

For Manuscript Review

Oligosaccharide Chain K	
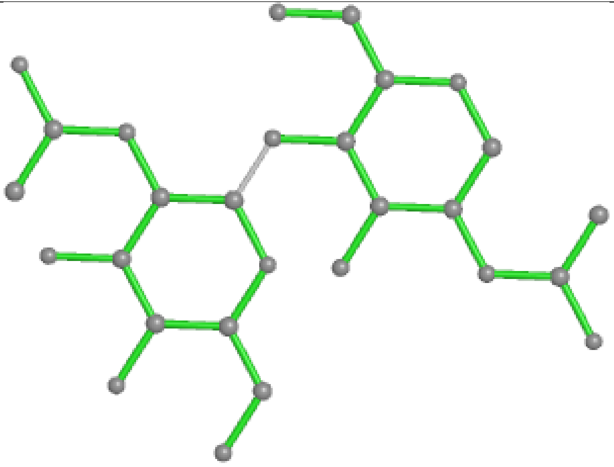 Bond lengths	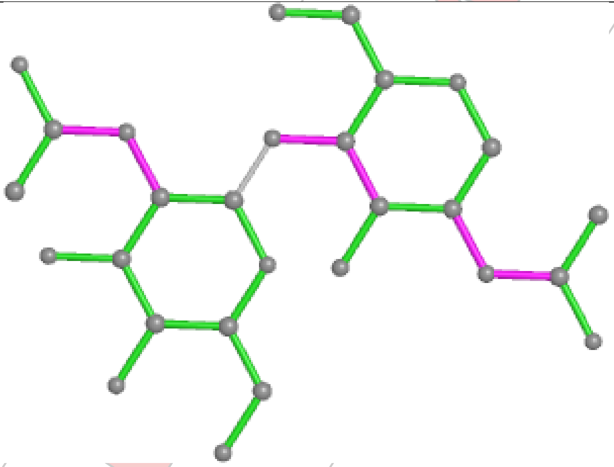 Bond angles
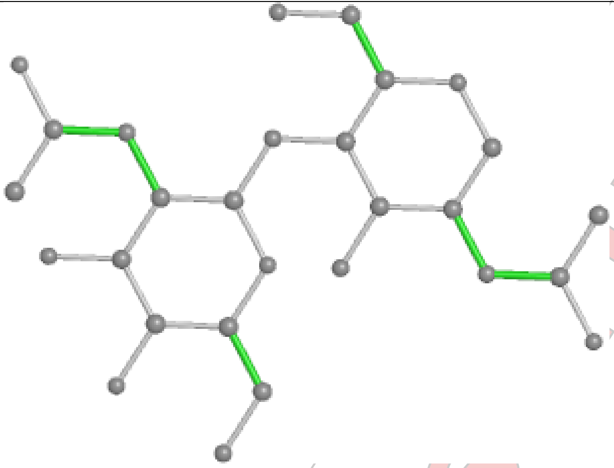 Torsions	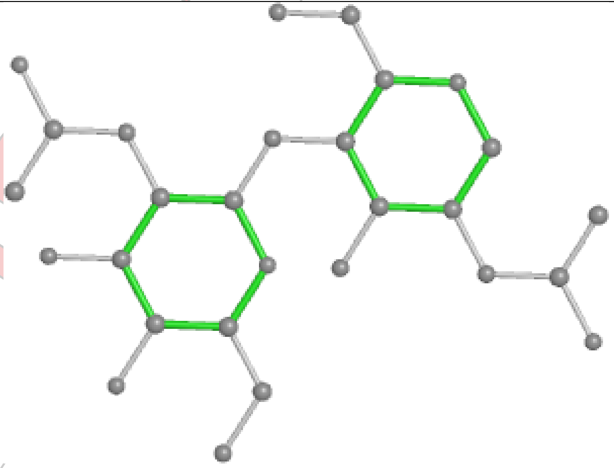 Rings

For Manuscript Review

Oligosaccharide Chain N	
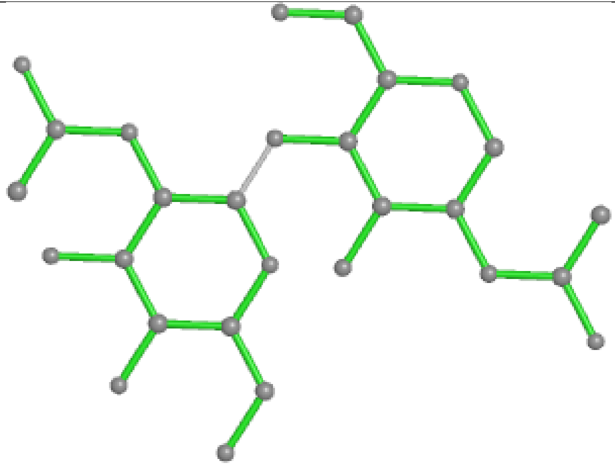 Bond lengths	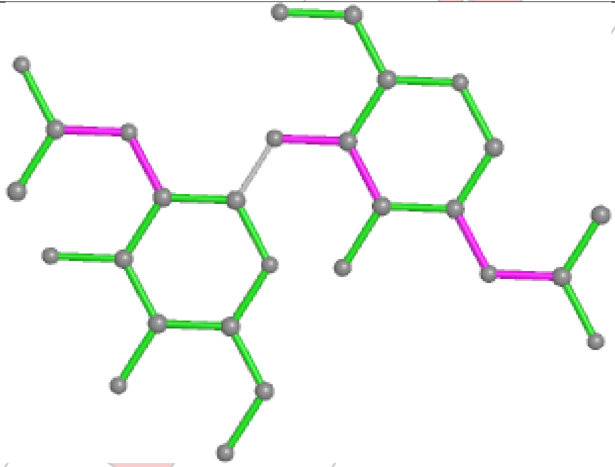 Bond angles
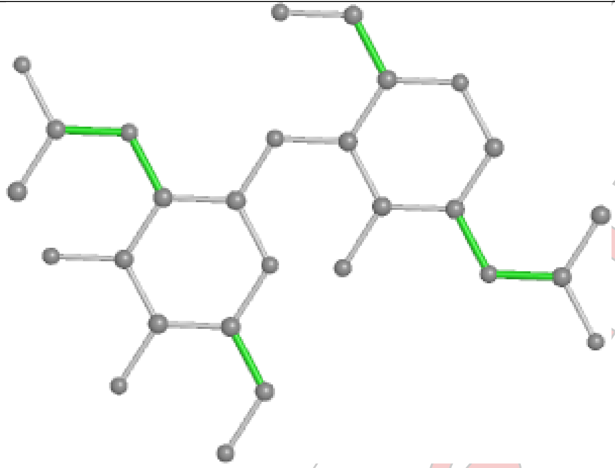 Torsions	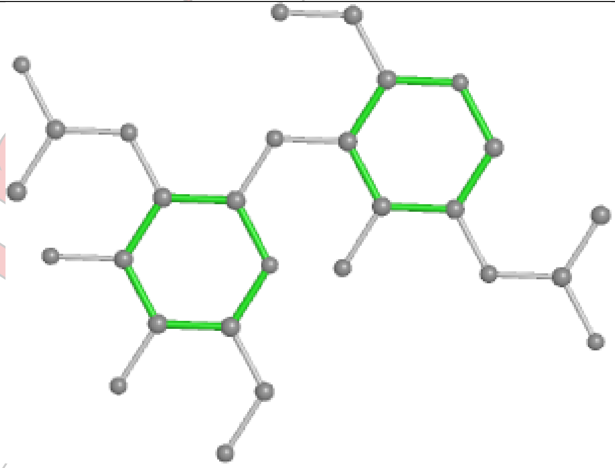 Rings

For Manuscript Review

Oligosaccharide Chain J

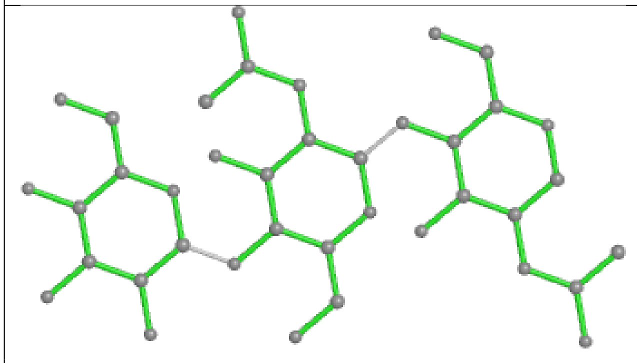
Bond lengths

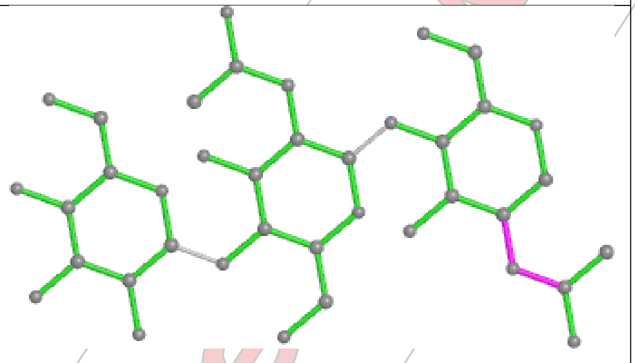
Bond angles

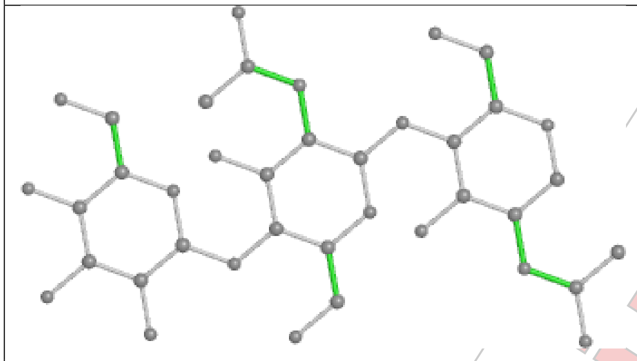
Torsions

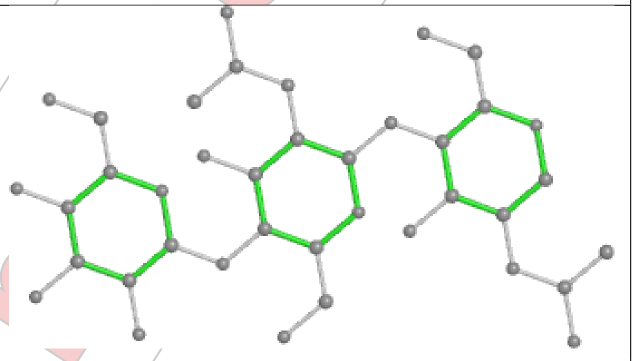
Rings

Oligosaccharide Chain M

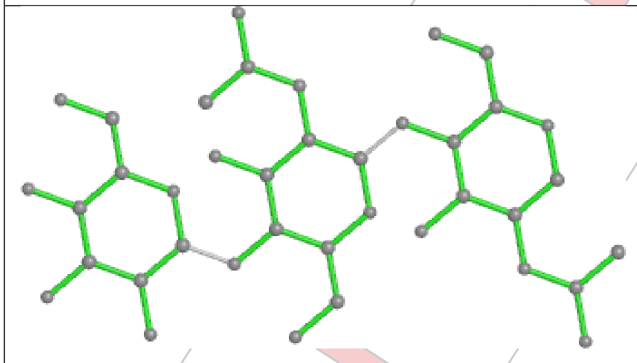
Bond lengths

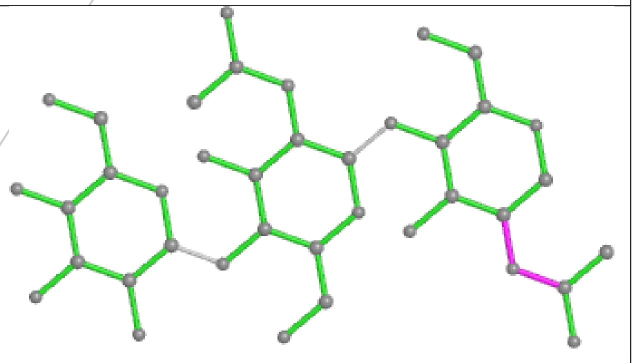
Bond angles

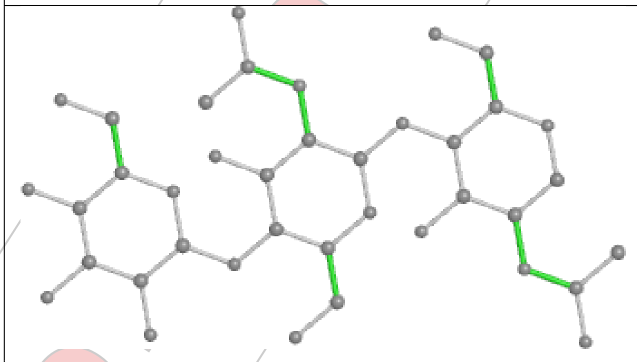
Torsions

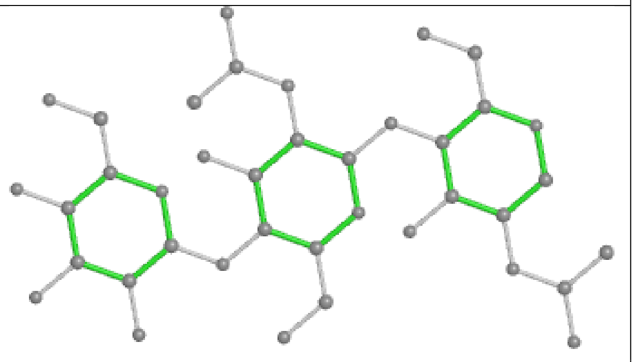
Rings

Oligosaccharide Chain O

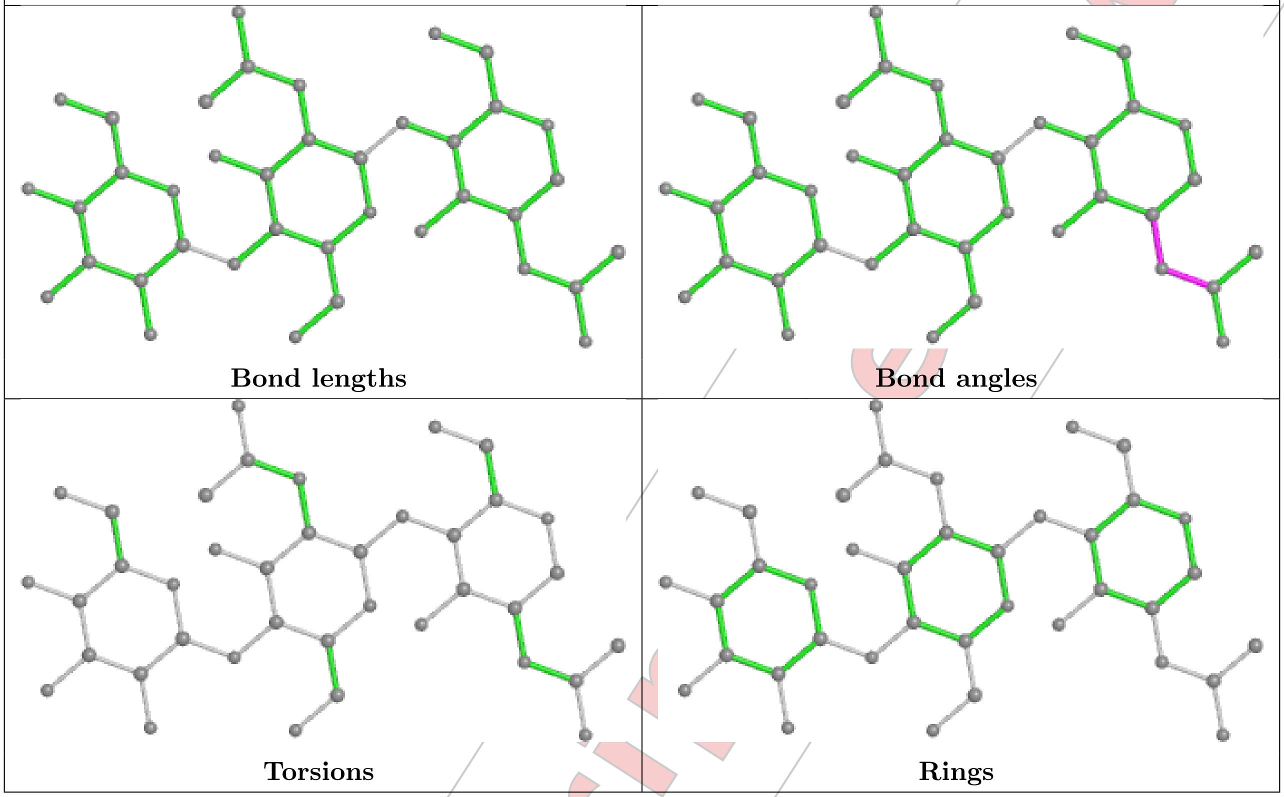

5.6 Ligand geometry (i)

45 ligands are modelled in this entry.

In the following table, the Counts columns list the number of bonds (or angles) for which Mogul statistics could be retrieved, the number of bonds (or angles) that are observed in the model and the number of bonds (or angles) that are defined in the Chemical Component Dictionary. The Link column lists molecule types, if any, to which the group is linked. The Z score for a bond length (or angle) is the number of standard deviations the observed value is removed from the expected value. A bond length (or angle) with $|Z| > 2$ is considered an outlier worth inspection. RMSZ is the root-mean-square of all Z scores of the bond lengths (or angles).

Mol	Type	Chain	Res	Link	Bond lengths			Bond angles		
					Counts	RMSZ	$\#\|Z\| > 2$	Counts	RMSZ	$\#\|Z\| > 2$
6	NAG	A	1313	1	14,14,15	1.09	1 (7%)	17,19,21	1.03	1 (5%)
6	NAG	B	1310	1	14,14,15	0.95	1 (7%)	17,19,21	1.29	4 (23%)
6	NAG	B	1311	-	14,14,15	1.06	1 (7%)	17,19,21	1.02	2 (11%)
6	NAG	F	1310	1	14,14,15	0.94	1 (7%)	17,19,21	1.29	4 (23%)
6	NAG	F	1303	1	14,14,15	0.43	0	17,19,21	0.58	0
6	NAG	A	1314	1	14,14,15	0.94	1 (7%)	17,19,21	1.06	1 (5%)
6	NAG	F	1304	1	14,14,15	1.29	2 (14%)	17,19,21	1.32	3 (17%)
6	NAG	F	1307	1	14,14,15	0.99	1 (7%)	17,19,21	1.01	1 (5%)

For Manuscript Review

Mol	Type	Chain	Res	Link	Bond lengths			Bond angles		
					Counts	RMSZ	#|Z| > 2	Counts	RMSZ	#|Z| > 2
6	NAG	F	1306	1	14,14,15	0.96	1 (7%)	17,19,21	1.03	1 (5%)
6	NAG	A	1303	1	14,14,15	0.42	0	17,19,21	0.58	0
6	NAG	B	1302	1	14,14,15	0.41	0	17,19,21	0.72	0
6	NAG	B	1301	1	14,14,15	0.40	0	17,19,21	0.65	0
6	NAG	A	1304	1	14,14,15	1.28	2 (14%)	17,19,21	1.31	3 (17%)
6	NAG	A	1307	1	14,14,15	0.99	1 (7%)	17,19,21	1.01	1 (5%)
6	NAG	B	1306	1	14,14,15	0.97	1 (7%)	17,19,21	1.03	1 (5%)
6	NAG	F	1308	1	14,14,15	0.92	1 (7%)	17,19,21	1.33	4 (23%)
6	NAG	B	1308	1	14,14,15	0.92	1 (7%)	17,19,21	1.33	4 (23%)
6	NAG	F	1312	1	14,14,15	1.12	1 (7%)	17,19,21	0.97	0
6	NAG	B	1305	1	14,14,15	0.93	1 (7%)	17,19,21	1.00	1 (5%)
6	NAG	B	1314	1	14,14,15	0.93	1 (7%)	17,19,21	1.06	1 (5%)
6	NAG	A	1305	1	14,14,15	0.94	1 (7%)	17,19,21	1.00	1 (5%)
6	NAG	A	1315	-	14,14,15	1.21	1 (7%)	17,19,21	1.60	4 (23%)
6	NAG	F	1305	1	14,14,15	0.93	1 (7%)	17,19,21	0.99	1 (5%)
6	NAG	A	1310	1	14,14,15	0.94	1 (7%)	17,19,21	1.29	4 (23%)
6	NAG	A	1311	-	14,14,15	1.06	1 (7%)	17,19,21	1.02	2 (11%)
6	NAG	B	1307	1	14,14,15	0.98	1 (7%)	17,19,21	1.01	1 (5%)
6	NAG	F	1313	1	14,14,15	1.08	1 (7%)	17,19,21	1.02	1 (5%)
6	NAG	F	1311	-	14,14,15	1.05	1 (7%)	17,19,21	1.01	2 (11%)
6	NAG	F	1309	-	14,14,15	1.02	1 (7%)	17,19,21	1.06	2 (11%)
6	NAG	F	1315	-	14,14,15	1.20	1 (7%)	17,19,21	1.60	4 (23%)
6	NAG	F	1314	1	14,14,15	0.95	1 (7%)	17,19,21	1.06	1 (5%)
6	NAG	B	1315	-	14,14,15	1.20	1 (7%)	17,19,21	1.60	4 (23%)
6	NAG	B	1304	1	14,14,15	1.29	2 (14%)	17,19,21	1.31	3 (17%)
6	NAG	A	1309	-	14,14,15	1.02	1 (7%)	17,19,21	1.06	2 (11%)
6	NAG	A	1306	1	14,14,15	0.97	1 (7%)	17,19,21	1.03	1 (5%)
6	NAG	A	1308	1	14,14,15	0.92	1 (7%)	17,19,21	1.33	4 (23%)
6	NAG	A	1302	1	14,14,15	0.41	0	17,19,21	0.72	0
6	NAG	A	1312	1	14,14,15	1.11	1 (7%)	17,19,21	0.97	0
6	NAG	B	1312	1	14,14,15	1.12	1 (7%)	17,19,21	0.97	0
6	NAG	F	1302	1	14,14,15	0.40	0	17,19,21	0.72	0
6	NAG	A	1301	1	14,14,15	0.42	0	17,19,21	0.65	0
6	NAG	B	1303	1	14,14,15	0.43	0	17,19,21	0.57	0
6	NAG	F	1301	1	14,14,15	0.41	0	17,19,21	0.65	0
6	NAG	B	1309	-	14,14,15	1.02	1 (7%)	17,19,21	1.06	2 (11%)

For Manuscript Review

Mol	Type	Chain	Res	Link	Bond lengths			Bond angles		
					Counts	RMSZ	#|Z| > 2	Counts	RMSZ	#|Z| > 2
6	NAG	B	1313	1	14,14,15	1.09	1 (7%)	17,19,21	1.03	1 (5%)

In the following table, the Chirals column lists the number of chiral outliers, the number of chiral centers analysed, the number of these observed in the model and the number defined in the Chemical Component Dictionary. Similar counts are reported in the Torsion and Rings columns. '-' means no outliers of that kind were identified.

Mol	Type	Chain	Res	Link	Chirals	Torsions	Rings
6	NAG	A	1313	1	-	0/6/23/26	0/1/1/1
6	NAG	B	1310	1	-	0/6/23/26	0/1/1/1
6	NAG	B	1311	-	-	0/6/23/26	0/1/1/1
6	NAG	F	1310	1	-	0/6/23/26	0/1/1/1
6	NAG	F	1303	1	-	0/6/23/26	0/1/1/1
6	NAG	A	1314	1	-	0/6/23/26	0/1/1/1
6	NAG	F	1304	1	-	0/6/23/26	0/1/1/1
6	NAG	F	1307	1	-	0/6/23/26	0/1/1/1
6	NAG	F	1306	1	-	0/6/23/26	0/1/1/1
6	NAG	A	1303	1	-	0/6/23/26	0/1/1/1
6	NAG	B	1302	1	-	0/6/23/26	0/1/1/1
6	NAG	B	1301	1	-	0/6/23/26	0/1/1/1
6	NAG	A	1304	1	-	0/6/23/26	0/1/1/1
6	NAG	A	1307	1	-	0/6/23/26	0/1/1/1
6	NAG	B	1306	1	-	0/6/23/26	0/1/1/1
6	NAG	F	1308	1	-	0/6/23/26	0/1/1/1
6	NAG	B	1308	1	-	0/6/23/26	0/1/1/1
6	NAG	F	1312	1	-	0/6/23/26	0/1/1/1
6	NAG	B	1305	1	-	0/6/23/26	0/1/1/1
6	NAG	B	1314	1	-	0/6/23/26	0/1/1/1
6	NAG	A	1305	1	-	0/6/23/26	0/1/1/1
6	NAG	A	1315	-	-	0/6/23/26	0/1/1/1
6	NAG	F	1305	1	-	0/6/23/26	0/1/1/1
6	NAG	A	1310	1	-	0/6/23/26	0/1/1/1
6	NAG	A	1311	-	-	0/6/23/26	0/1/1/1
6	NAG	B	1307	1	-	0/6/23/26	0/1/1/1
6	NAG	F	1313	1	-	0/6/23/26	0/1/1/1
6	NAG	F	1311	-	-	0/6/23/26	0/1/1/1
6	NAG	F	1309	-	-	0/6/23/26	0/1/1/1
6	NAG	F	1315	-	-	0/6/23/26	0/1/1/1
6	NAG	F	1314	1	-	0/6/23/26	0/1/1/1
6	NAG	B	1315	-	-	0/6/23/26	0/1/1/1
6	NAG	B	1304	1	-	0/6/23/26	0/1/1/1
6	NAG	A	1309	-	-	0/6/23/26	0/1/1/1

Continued on next page...

For Manuscript Review

Continued from previous page...

Mol	Type	Chain	Res	Link	Chirals	Torsions	Rings
6	NAG	A	1306	1	-	0/6/23/26	0/1/1/1
6	NAG	A	1308	1	-	0/6/23/26	0/1/1/1
6	NAG	A	1302	1	-	0/6/23/26	0/1/1/1
6	NAG	A	1312	1	-	0/6/23/26	0/1/1/1
6	NAG	B	1312	1	-	0/6/23/26	0/1/1/1
6	NAG	F	1302	1	-	0/6/23/26	0/1/1/1
6	NAG	A	1301	1	-	0/6/23/26	0/1/1/1
6	NAG	B	1303	1	-	0/6/23/26	0/1/1/1
6	NAG	F	1301	1	-	0/6/23/26	0/1/1/1
6	NAG	B	1309	-	-	0/6/23/26	0/1/1/1
6	NAG	B	1313	1	-	0/6/23/26	0/1/1/1

All (39) bond length outliers are listed below:

Mol	Chain	Res	Type	Atoms	Z	Observed(Å)	Ideal(Å)
6	F	1312	NAG	C1-C2	3.41	1.57	1.52
6	B	1312	NAG	C1-C2	3.41	1.57	1.52
6	A	1312	NAG	C1-C2	3.39	1.57	1.52
6	A	1315	NAG	O5-C5	3.24	1.50	1.43
6	B	1315	NAG	O5-C5	3.22	1.50	1.43
6	F	1315	NAG	O5-C5	3.21	1.50	1.43
6	A	1313	NAG	C1-C2	3.09	1.57	1.52
6	B	1313	NAG	C1-C2	3.07	1.56	1.52
6	F	1313	NAG	C1-C2	3.03	1.56	1.52
6	A	1307	NAG	C1-C2	2.66	1.56	1.52
6	F	1307	NAG	C1-C2	2.65	1.56	1.52
6	B	1307	NAG	C1-C2	2.64	1.56	1.52
6	B	1304	NAG	C1-C2	2.57	1.56	1.52
6	F	1304	NAG	C1-C2	2.56	1.56	1.52
6	A	1304	NAG	C1-C2	2.53	1.56	1.52
6	B	1304	NAG	O5-C5	2.52	1.48	1.43
6	F	1304	NAG	O5-C5	2.51	1.48	1.43
6	A	1304	NAG	O5-C5	2.49	1.48	1.43
6	B	1306	NAG	C1-C2	2.48	1.56	1.52
6	A	1306	NAG	C1-C2	2.46	1.56	1.52
6	F	1314	NAG	C1-C2	2.44	1.56	1.52
6	F	1306	NAG	C1-C2	2.44	1.56	1.52
6	A	1314	NAG	C1-C2	2.39	1.55	1.52
6	B	1314	NAG	C1-C2	2.37	1.55	1.52
6	A	1305	NAG	C1-C2	2.34	1.55	1.52
6	F	1305	NAG	C1-C2	2.33	1.55	1.52
6	B	1305	NAG	C1-C2	2.32	1.55	1.52

Continued on next page...

For Manuscript Review

Continued from previous page...

Mol	Chain	Res	Type	Atoms	Z	Observed(Å)	Ideal(Å)
6	A	1311	NAG	C1-C2	2.31	1.55	1.52
6	F	1311	NAG	C1-C2	2.30	1.55	1.52
6	B	1311	NAG	C1-C2	2.30	1.55	1.52
6	B	1310	NAG	C1-C2	2.18	1.55	1.52
6	A	1310	NAG	C1-C2	2.16	1.55	1.52
6	F	1310	NAG	C1-C2	2.14	1.55	1.52
6	A	1309	NAG	C1-C2	2.12	1.55	1.52
6	B	1309	NAG	C1-C2	2.11	1.55	1.52
6	F	1309	NAG	C1-C2	2.06	1.55	1.52
6	A	1308	NAG	C1-C2	2.05	1.55	1.52
6	B	1308	NAG	C1-C2	2.04	1.55	1.52
6	F	1308	NAG	C1-C2	2.04	1.55	1.52

All (72) bond angle outliers are listed below:

Mol	Chain	Res	Type	Atoms	Z	Observed(^{o})	Ideal(^{o})
6	A	1315	NAG	O5-C5-C6	3.24	112.28	107.20
6	F	1315	NAG	O5-C5-C6	3.23	112.26	107.20
6	B	1315	NAG	O5-C5-C6	3.23	112.26	107.20
6	F	1315	NAG	C8-C7-N2	2.76	120.78	116.10
6	A	1315	NAG	C8-C7-N2	2.76	120.76	116.10
6	B	1315	NAG	C8-C7-N2	2.74	120.74	116.10
6	B	1304	NAG	C8-C7-N2	2.54	120.40	116.10
6	F	1304	NAG	C8-C7-N2	2.52	120.37	116.10
6	A	1304	NAG	C8-C7-N2	2.51	120.34	116.10
6	B	1306	NAG	C8-C7-N2	2.46	120.27	116.10
6	F	1306	NAG	C8-C7-N2	2.46	120.26	116.10
6	A	1306	NAG	C8-C7-N2	2.46	120.26	116.10
6	F	1314	NAG	C8-C7-N2	2.41	120.19	116.10
6	B	1314	NAG	C8-C7-N2	2.41	120.18	116.10
6	A	1314	NAG	C8-C7-N2	2.41	120.18	116.10
6	B	1308	NAG	C4-C3-C2	-2.38	107.53	111.02
6	A	1308	NAG	C4-C3-C2	-2.37	107.54	111.02
6	F	1308	NAG	C4-C3-C2	-2.37	107.55	111.02
6	B	1305	NAG	C8-C7-N2	2.37	120.11	116.10
6	F	1309	NAG	C8-C7-N2	2.36	120.10	116.10
6	A	1305	NAG	C8-C7-N2	2.36	120.09	116.10
6	A	1309	NAG	C8-C7-N2	2.36	120.09	116.10
6	B	1309	NAG	C8-C7-N2	2.35	120.08	116.10
6	F	1305	NAG	C8-C7-N2	2.34	120.06	116.10
6	F	1310	NAG	C8-C7-N2	2.31	120.02	116.10
6	B	1310	NAG	C8-C7-N2	2.30	120.00	116.10

Continued on next page...

For Manuscript Review

Continued from previous page...

Mol	Chain	Res	Type	Atoms	Z	Observed(°)	Ideal(°)
6	A	1310	NAG	C8-C7-N2	2.30	120.00	116.10
6	B	1310	NAG	O4-C4-C5	-2.26	103.68	109.30
6	A	1310	NAG	O4-C4-C5	-2.26	103.69	109.30
6	F	1310	NAG	O4-C4-C5	-2.26	103.69	109.30
6	A	1310	NAG	C4-C3-C2	-2.22	107.76	111.02
6	A	1315	NAG	C1-C2-N2	-2.22	106.70	110.49
6	A	1308	NAG	C8-C7-N2	2.22	119.85	116.10
6	F	1310	NAG	C4-C3-C2	-2.21	107.77	111.02
6	B	1308	NAG	O4-C4-C3	-2.21	105.24	110.35
6	B	1315	NAG	C1-C2-N2	-2.21	106.72	110.49
6	F	1308	NAG	C8-C7-N2	2.21	119.84	116.10
6	B	1310	NAG	C4-C3-C2	-2.21	107.78	111.02
6	F	1315	NAG	C1-C2-N2	-2.20	106.72	110.49
6	A	1308	NAG	O4-C4-C3	-2.20	105.25	110.35
6	B	1308	NAG	C2-N2-C7	-2.20	119.77	122.90
6	F	1308	NAG	O4-C4-C3	-2.20	105.26	110.35
6	A	1310	NAG	C2-N2-C7	-2.20	119.77	122.90
6	B	1310	NAG	C2-N2-C7	-2.19	119.78	122.90
6	A	1308	NAG	C2-N2-C7	-2.19	119.78	122.90
6	B	1308	NAG	C8-C7-N2	2.19	119.81	116.10
6	F	1311	NAG	C8-C7-N2	2.19	119.80	116.10
6	F	1308	NAG	C2-N2-C7	-2.19	119.79	122.90
6	B	1311	NAG	C8-C7-N2	2.18	119.79	116.10
6	F	1310	NAG	C2-N2-C7	-2.18	119.80	122.90
6	A	1304	NAG	C2-N2-C7	-2.18	119.80	122.90
6	F	1304	NAG	C2-N2-C7	-2.17	119.81	122.90
6	A	1311	NAG	C8-C7-N2	2.17	119.77	116.10
6	A	1309	NAG	C2-N2-C7	-2.16	119.82	122.90
6	B	1304	NAG	C2-N2-C7	-2.16	119.83	122.90
6	B	1309	NAG	C2-N2-C7	-2.16	119.83	122.90
6	F	1309	NAG	C2-N2-C7	-2.15	119.84	122.90
6	A	1313	NAG	C8-C7-N2	2.14	119.72	116.10
6	F	1313	NAG	C8-C7-N2	2.13	119.71	116.10
6	B	1313	NAG	C8-C7-N2	2.13	119.71	116.10
6	A	1307	NAG	C8-C7-N2	2.05	119.58	116.10
6	F	1304	NAG	C1-O5-C5	-2.05	109.41	112.19
6	F	1307	NAG	C8-C7-N2	2.05	119.57	116.10
6	B	1311	NAG	C2-N2-C7	-2.05	119.99	122.90
6	B	1307	NAG	C8-C7-N2	2.04	119.56	116.10
6	A	1311	NAG	C2-N2-C7	-2.04	119.99	122.90
6	A	1304	NAG	C1-O5-C5	-2.04	109.43	112.19
6	B	1315	NAG	O4-C4-C3	-2.04	105.64	110.35

Continued on next page...

For Manuscript Review

Continued from previous page...

Mol	Chain	Res	Type	Atoms	Z	Observed(°)	Ideal(°)
6	F	1311	NAG	C2-N2-C7	-2.03	120.01	122.90
6	F	1315	NAG	O4-C4-C3	-2.03	105.65	110.35
6	A	1315	NAG	O4-C4-C3	-2.03	105.66	110.35
6	B	1304	NAG	C1-O5-C5	-2.03	109.45	112.19

There are no chirality outliers.

There are no torsion outliers.

There are no ring outliers.

15 monomers are involved in 16 short contacts:

Mol	Chain	Res	Type	Clashes	Symm-Clashes
6	B	1310	NAG	2	0
6	B	1311	NAG	2	0
6	F	1310	NAG	2	0
6	B	1301	NAG	1	0
6	F	1308	NAG	2	0
6	B	1308	NAG	3	0
6	A	1310	NAG	2	0
6	A	1311	NAG	2	0
6	F	1311	NAG	2	0
6	F	1309	NAG	2	0
6	A	1309	NAG	2	0
6	A	1308	NAG	2	0
6	A	1301	NAG	1	0
6	F	1301	NAG	1	0
6	B	1309	NAG	3	0

5.7 Other polymers (i)

There are no such residues in this entry.

5.8 Polymer linkage issues (i)

There are no chain breaks in this entry.

For Manuscript Review

6 Map visualisation (i)

This section contains visualisations of the EMDB entry EMD-45174. These allow visual inspection of the internal detail of the map and identification of artifacts.

Images derived from a raw map, generated by summing the deposited half-maps, are presented below the corresponding image components of the primary map to allow further visual inspection and comparison with those of the primary map.

6.1 Orthogonal projections (i)

6.1.1 Primary map

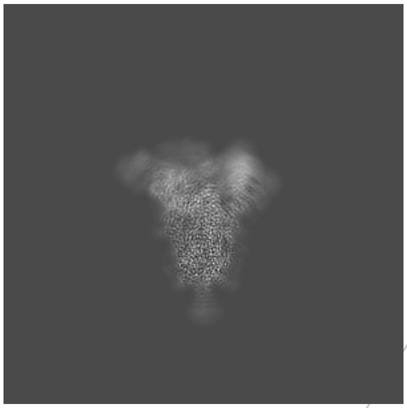
X

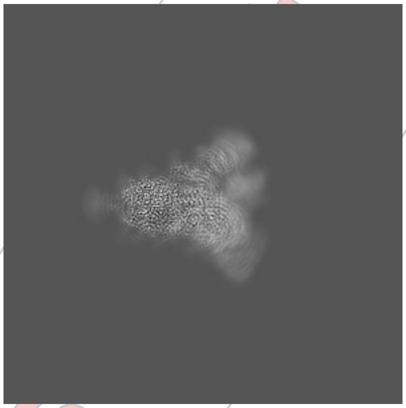
Y

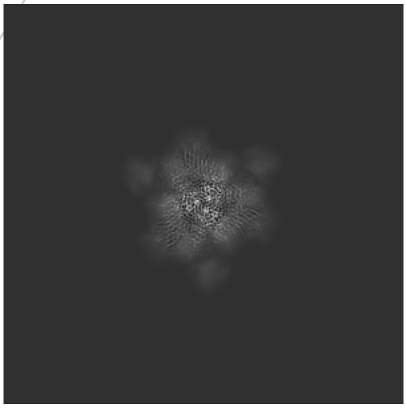
Z

6.1.2 Raw map

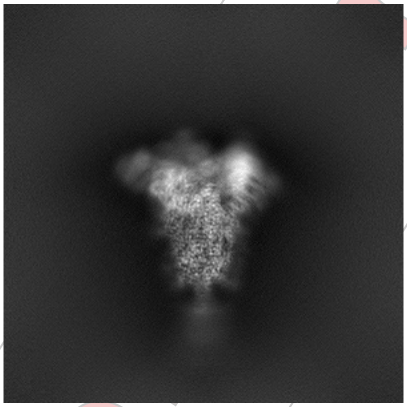
X

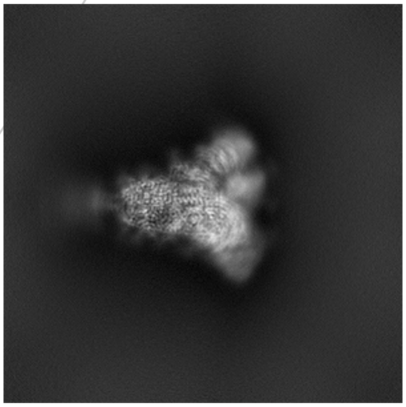
Y

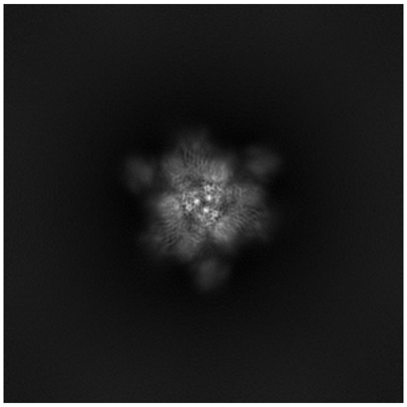
Z

The images above show the map projected in three orthogonal directions.

For Manuscript Review

6.2 Central slices (i)

6.2.1 Primary map

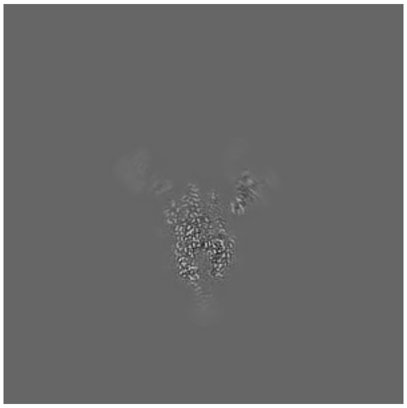

X Index: 256

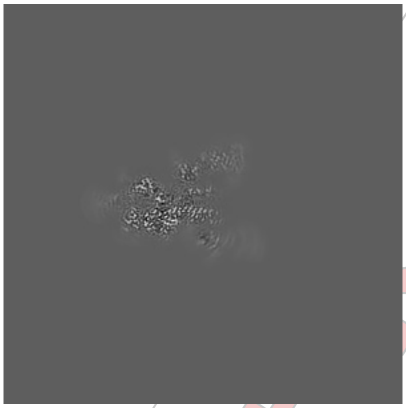

Y Index: 256

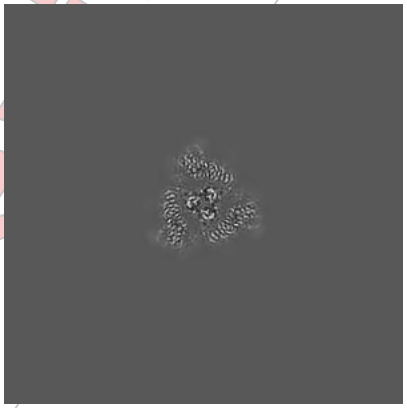

Z Index: 256

6.2.2 Raw map

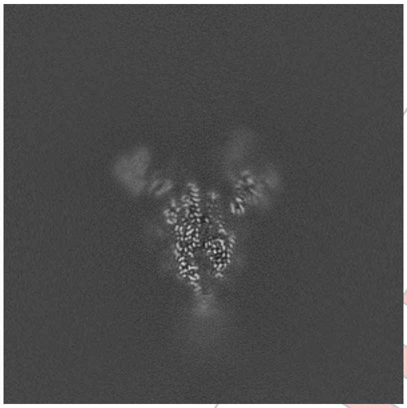

X Index: 256

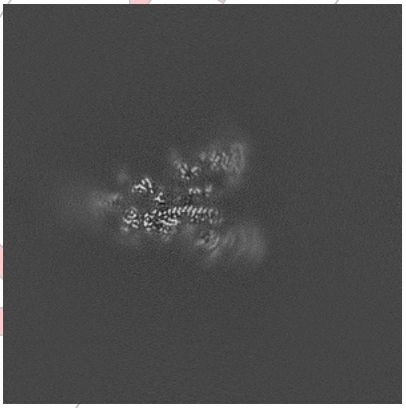

Y Index: 256

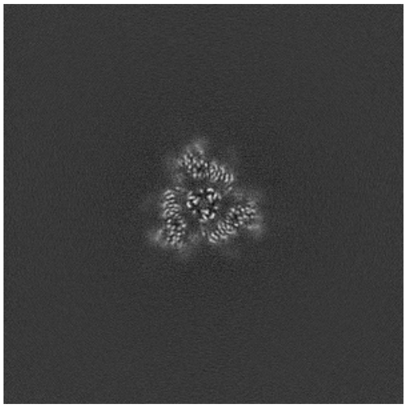

Z Index: 256

The images above show central slices of the map in three orthogonal directions.

For Manuscript Review

6.3 Largest variance slices (i)

6.3.1 Primary map

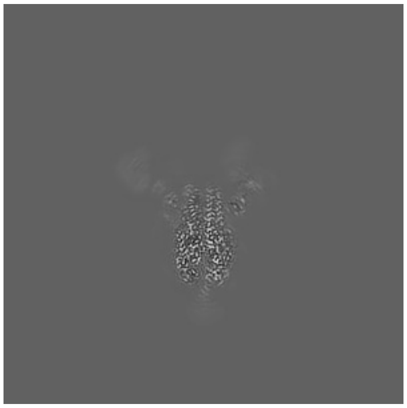
X Index: 263

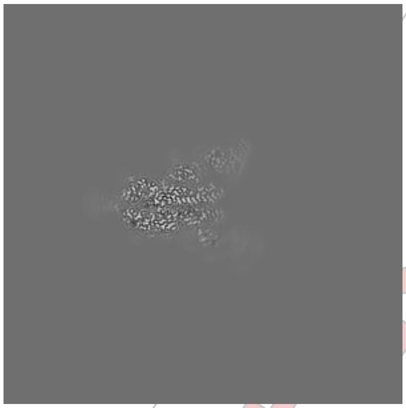
Y Index: 264

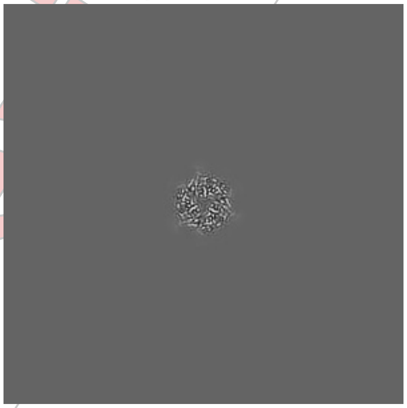
Z Index: 183

6.3.2 Raw map

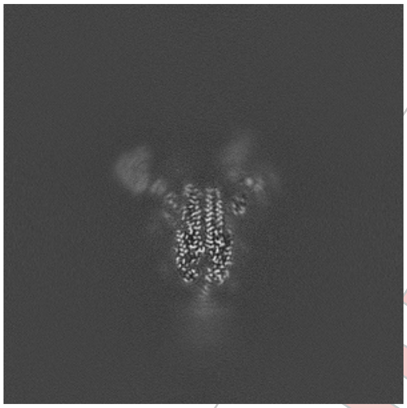
X Index: 262

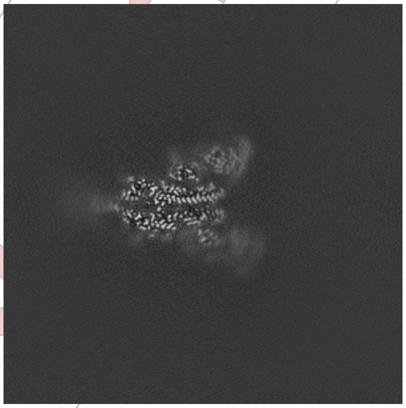
Y Index: 264

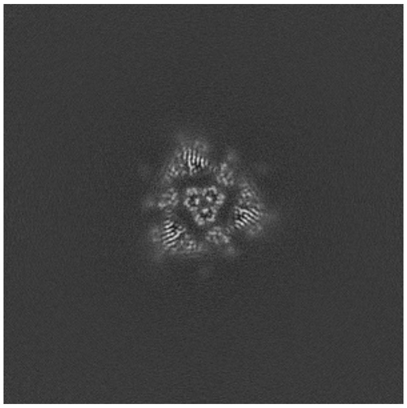
Z Index: 266

The images above show the largest variance slices of the map in three orthogonal directions.

For Manuscript Review

6.4 Orthogonal standard-deviation projections (False-color) (i)

6.4.1 Primary map

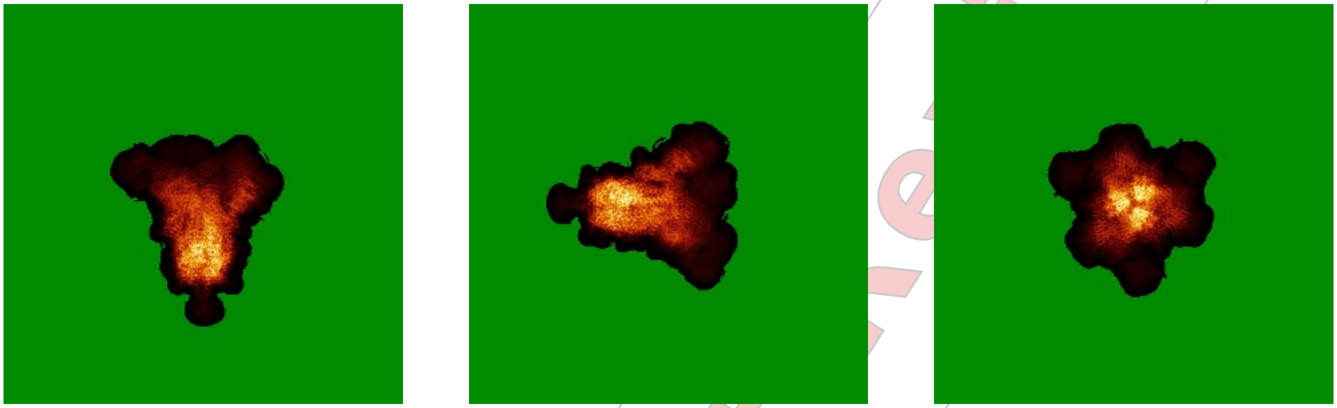

X Y Z

6.4.2 Raw map

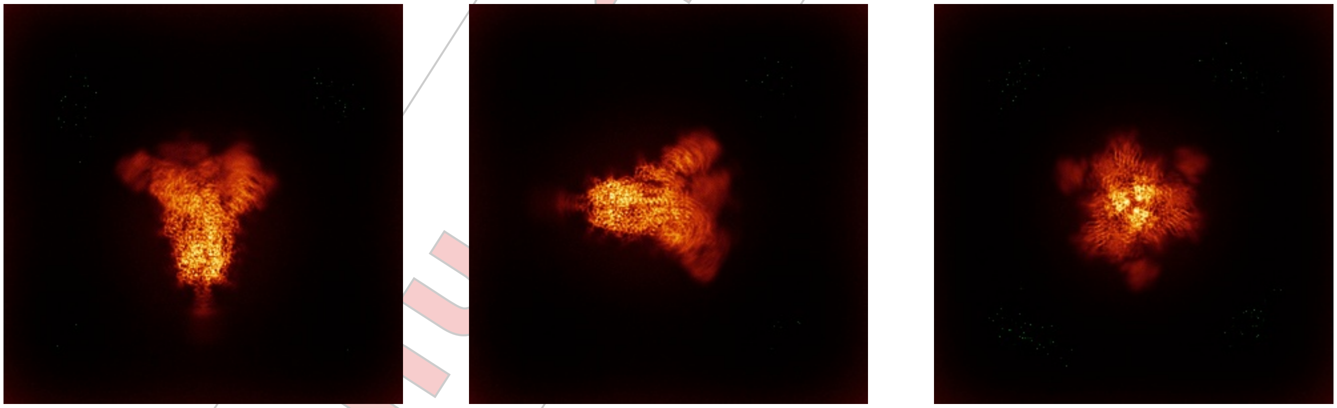

X Y Z

The images above show the map standard deviation projections with false color in three orthogonal directions. Minimum values are shown in green, max in blue, and dark to light orange shades represent small to large values respectively.

For Manuscript Review

6.5 Orthogonal surface views (i)

6.5.1 Primary map

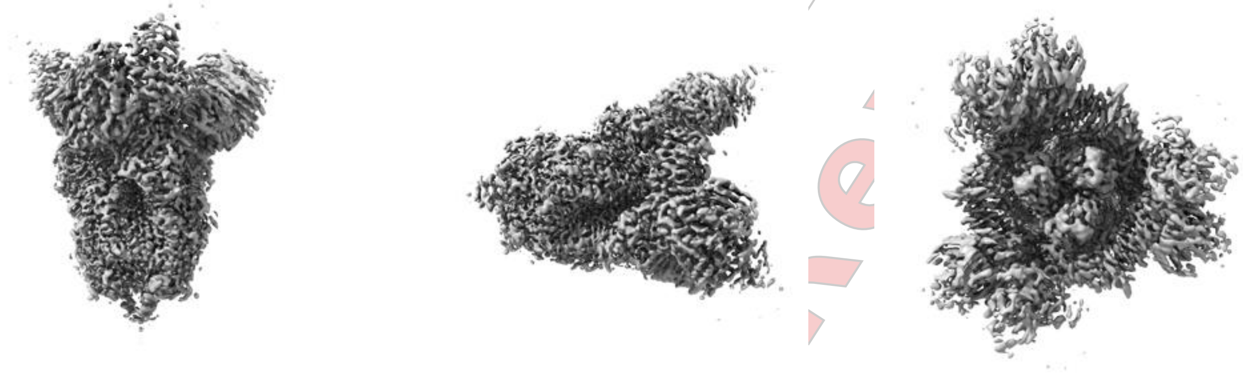

X Y Z

The images above show the 3D surface view of the map at the recommended contour level 0.6. These images, in conjunction with the slice images, may facilitate assessment of whether an appropriate contour level has been provided.

6.5.2 Raw map

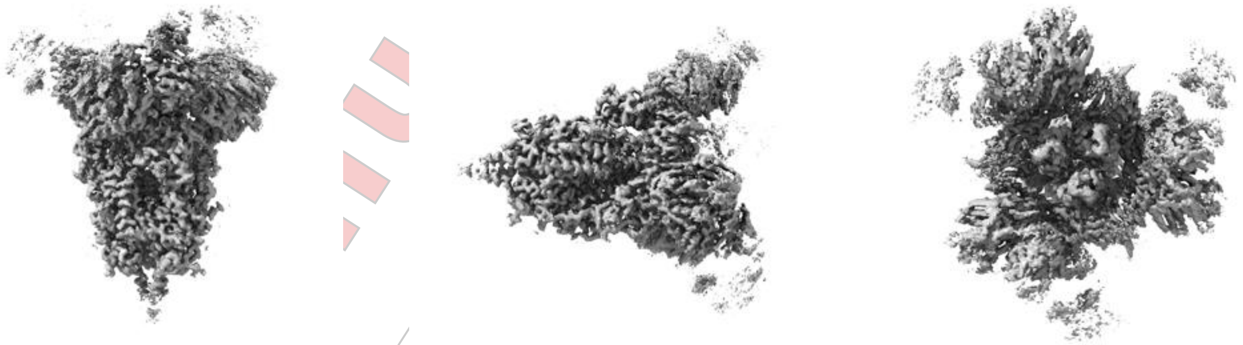

X Y Z

These images show the 3D surface of the raw map. The raw map's contour level was selected so that its surface encloses the same volume as the primary map does at its recommended contour level.

6.6 Mask visualisation (i)

This section was not generated. No masks/segmentation were deposited.

For Manuscript Review

7 Map analysis (i)

This section contains the results of statistical analysis of the map.

7.1 Map-value distribution (i)

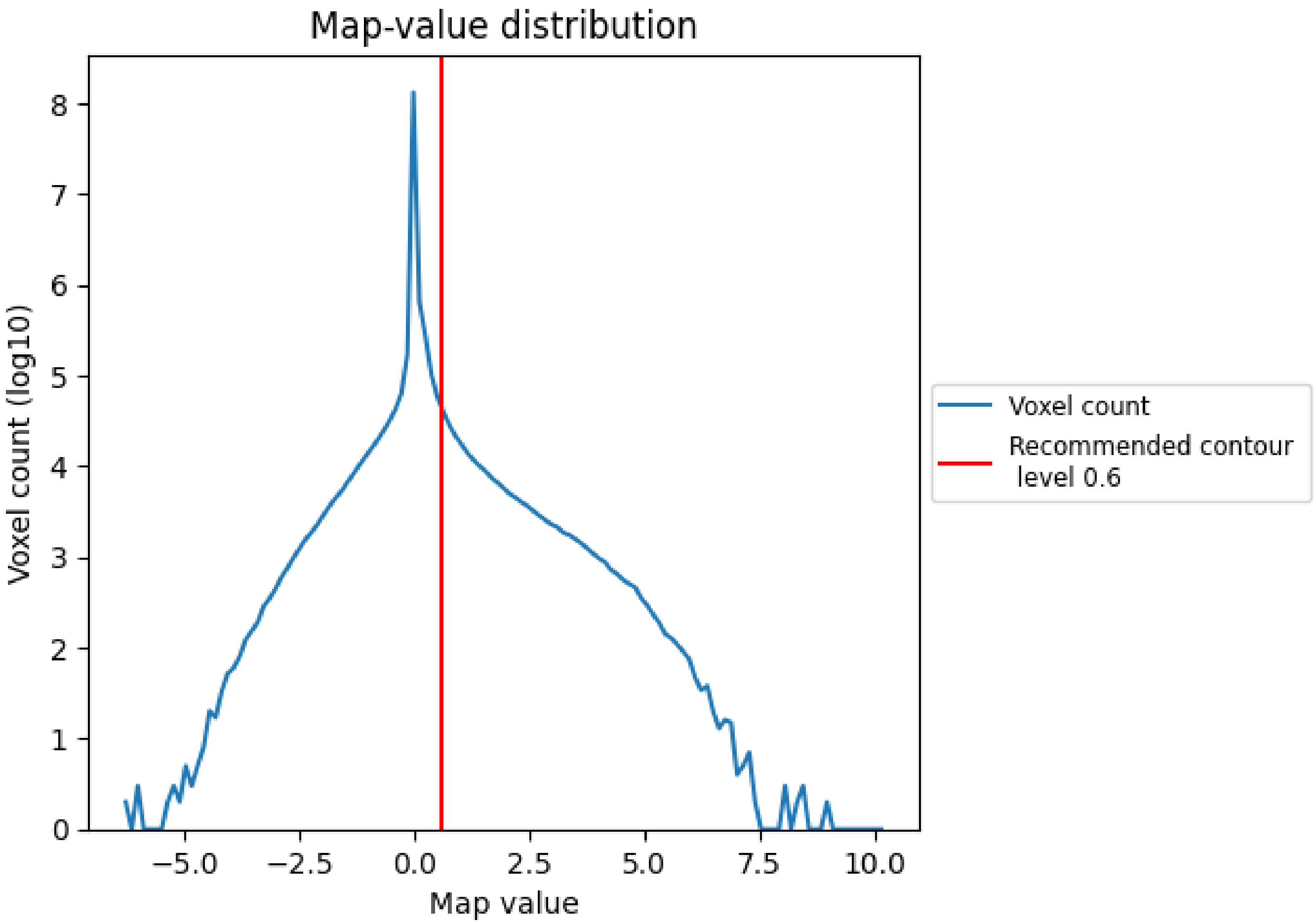

The map-value distribution is plotted in 128 intervals along the x-axis. The y-axis is logarithmic. A spike in this graph at zero usually indicates that the volume has been masked.

For Manuscript Review

7.2 Volume estimate (i)

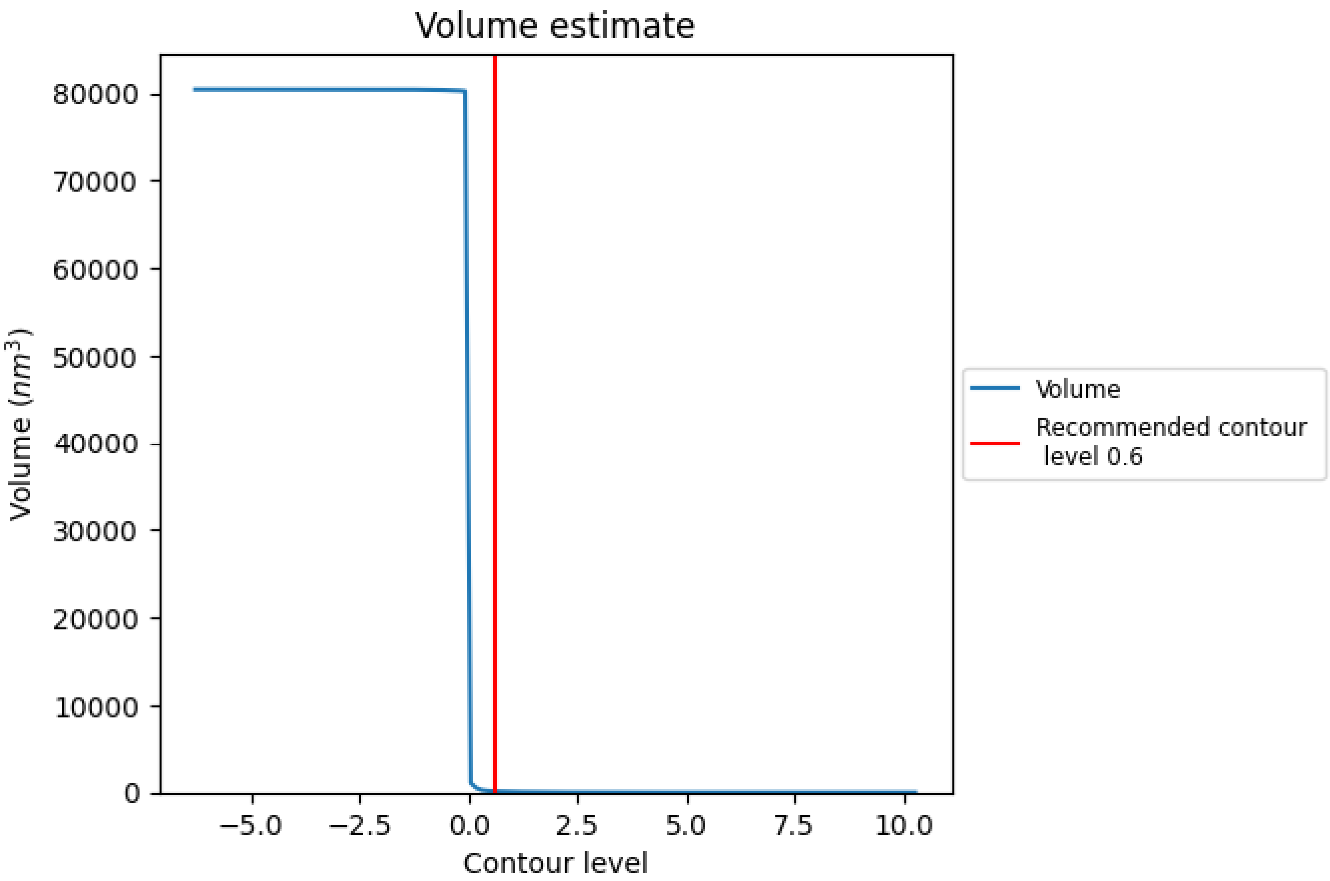

The volume at the recommended contour level is 137 nm^3; this corresponds to an approximate mass of 124 kDa.

The volume estimate graph shows how the enclosed volume varies with the contour level. The recommended contour level is shown as a vertical line and the intersection between the line and the curve gives the volume of the enclosed surface at the given level.

For Manuscript Review

7.3 Rotationally averaged power spectrum (i)

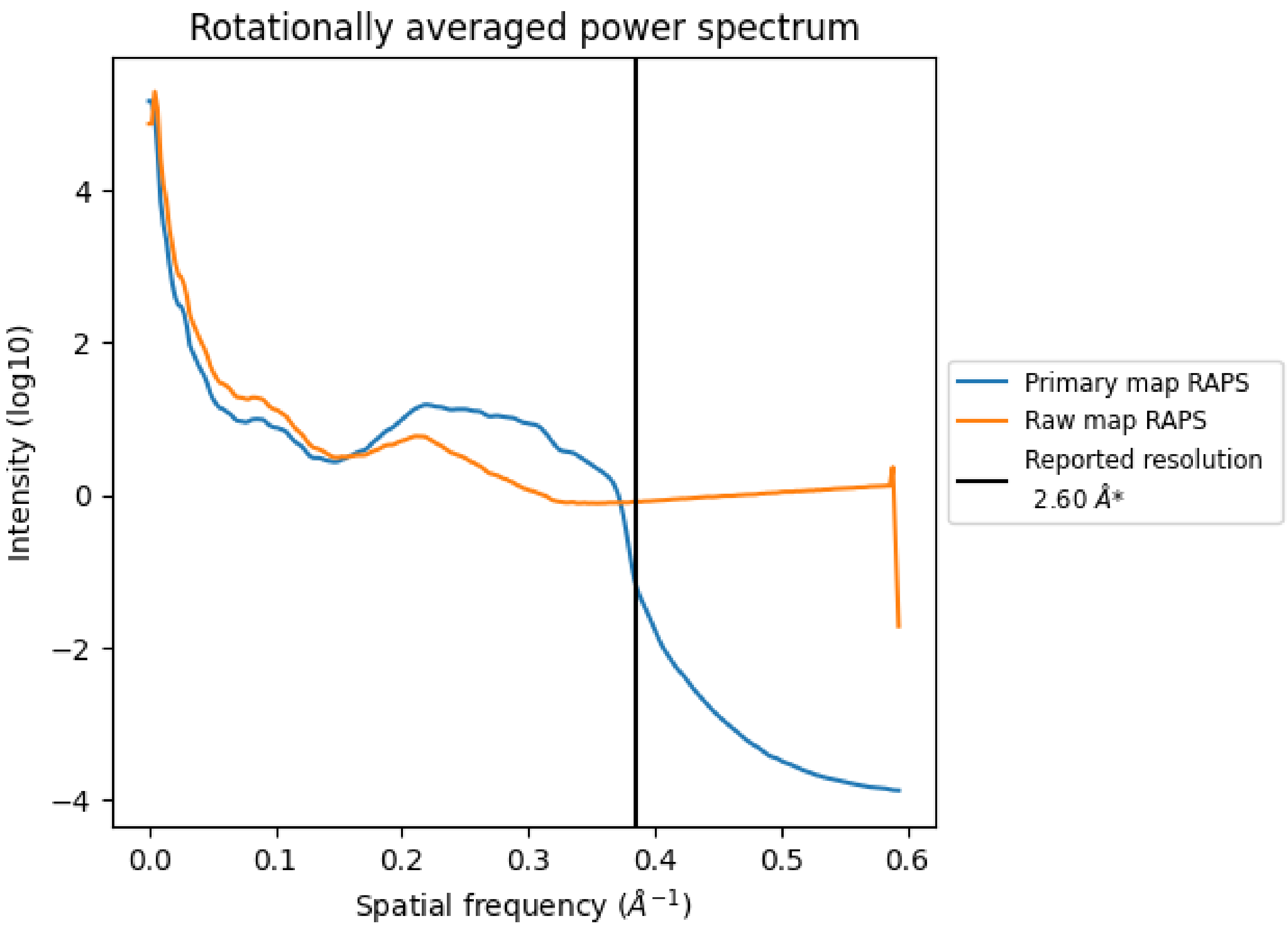

*Reported resolution corresponds to spatial frequency of 0.385 Å^{-1}

For Manuscript Review

8 Fourier-Shell correlation (i)

Fourier-Shell Correlation (FSC) is the most commonly used method to estimate the resolution of single-particle and subtomogram-averaged maps. The shape of the curve depends on the imposed symmetry, mask and whether or not the two 3D reconstructions used were processed from a common reference. The reported resolution is shown as a black line. A curve is displayed for the half-bit criterion in addition to lines showing the 0.143 gold standard cut-off and 0.5 cut-off.

8.1 FSC (i)

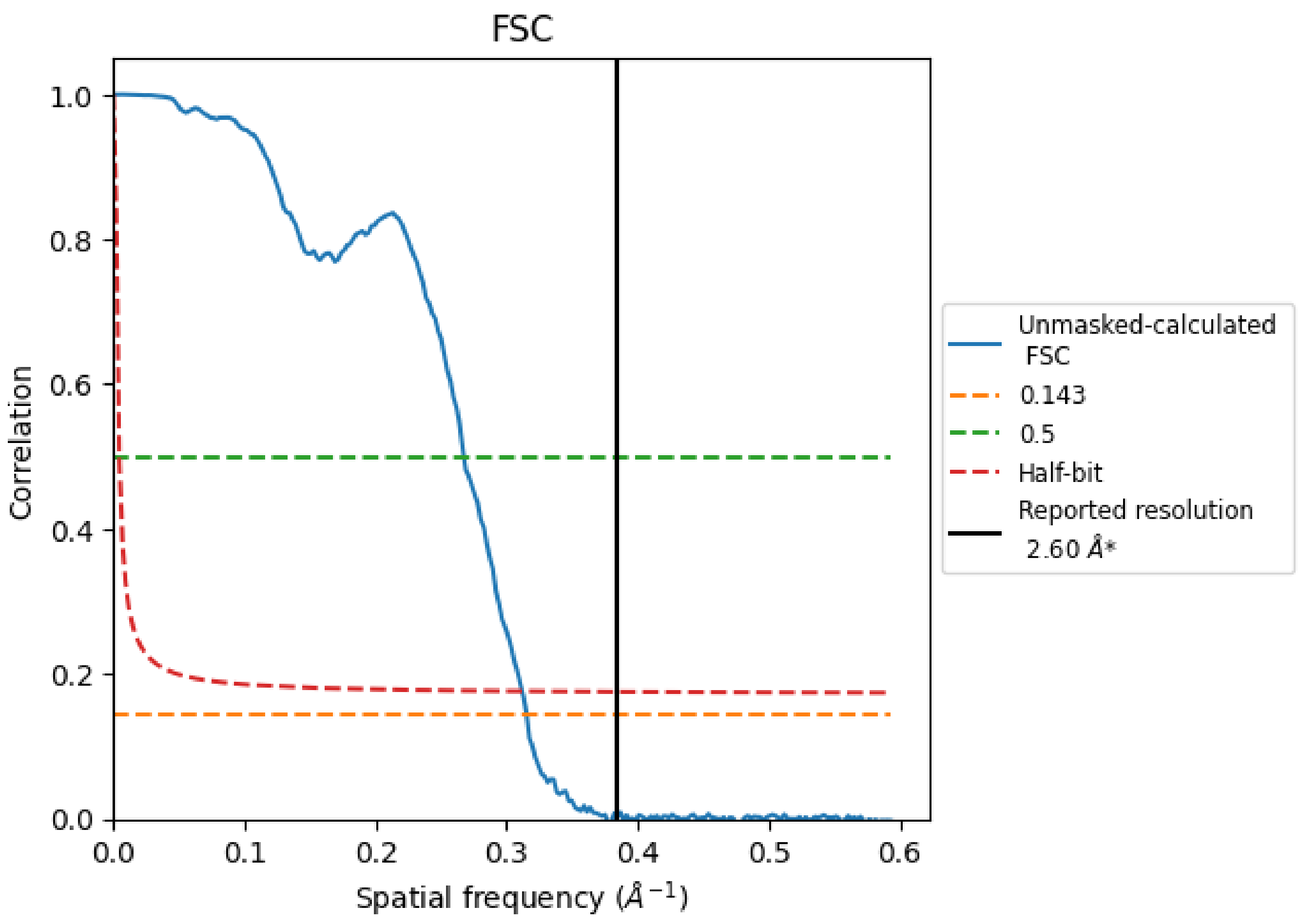

*Reported resolution corresponds to spatial frequency of 0.385 Å^{-1}

For Manuscript Review

8.2 Resolution estimates (i)

Resolution estimate (Å)	Estimation criterion (FSC cut-off)		
	0.143	0.5	Half-bit
Reported by author	2.60	-	-
Author-provided FSC curve	-	-	-
Unmasked-calculated*	3.17	3.74	3.21

*Resolution estimate based on FSC curve calculated by comparison of deposited half-maps. The value from deposited half-maps intersecting FSC 0.143 CUT-OFF 3.17 differs from the reported value 2.6 by more than 10 %

For Manuscript Review

9 Map-model fit (i)

This section contains information regarding the fit between EMDB map EMD-45174 and PDB model 9C44. Per-residue inclusion information can be found in section 3 on page 15.

9.1 Map-model overlay (i)

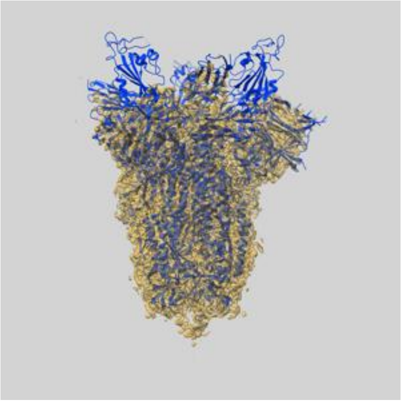

X

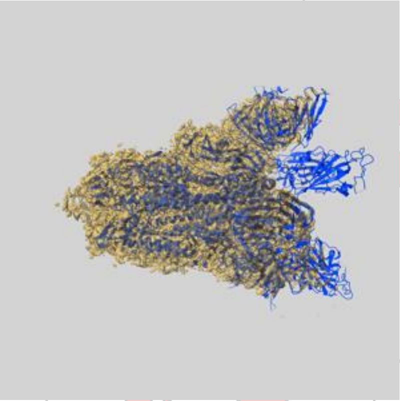

Y

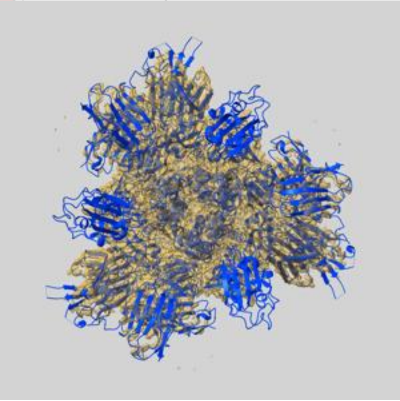

Z

The images above show the 3D surface view of the map at the recommended contour level 0.6 at 50% transparency in yellow overlaid with a ribbon representation of the model coloured in blue. These images allow for the visual assessment of the quality of fit between the atomic model and the map.

For Manuscript Review

9.2 Q-score mapped to coordinate model (i)

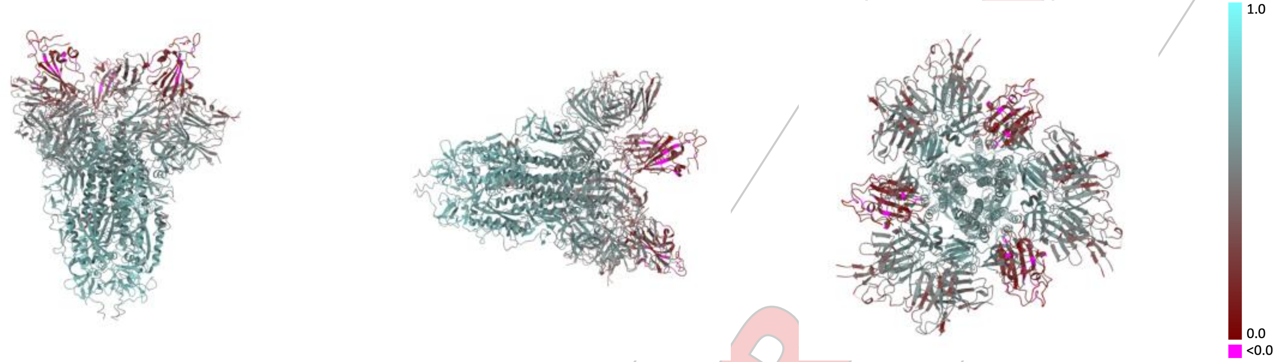

X Y Z

The images above show the model with each residue coloured according its Q-score. This shows their resolvability in the map with higher Q-score values reflecting better resolvability. Please note: Q-score is calculating the resolvability of atoms, and thus high values are only expected at resolutions at which atoms can be resolved. Low Q-score values may therefore be expected for many entries.

9.3 Atom inclusion mapped to coordinate model (i)

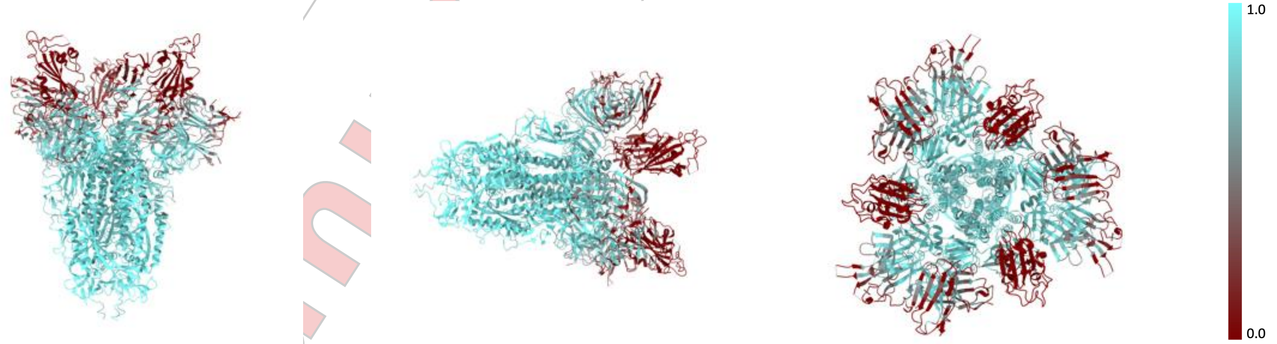

X Y Z

The images above show the model with each residue coloured according to its atom inclusion. This shows to what extent they are inside the map at the recommended contour level (0.6).

For Manuscript Review

9.4 Atom inclusion (i)

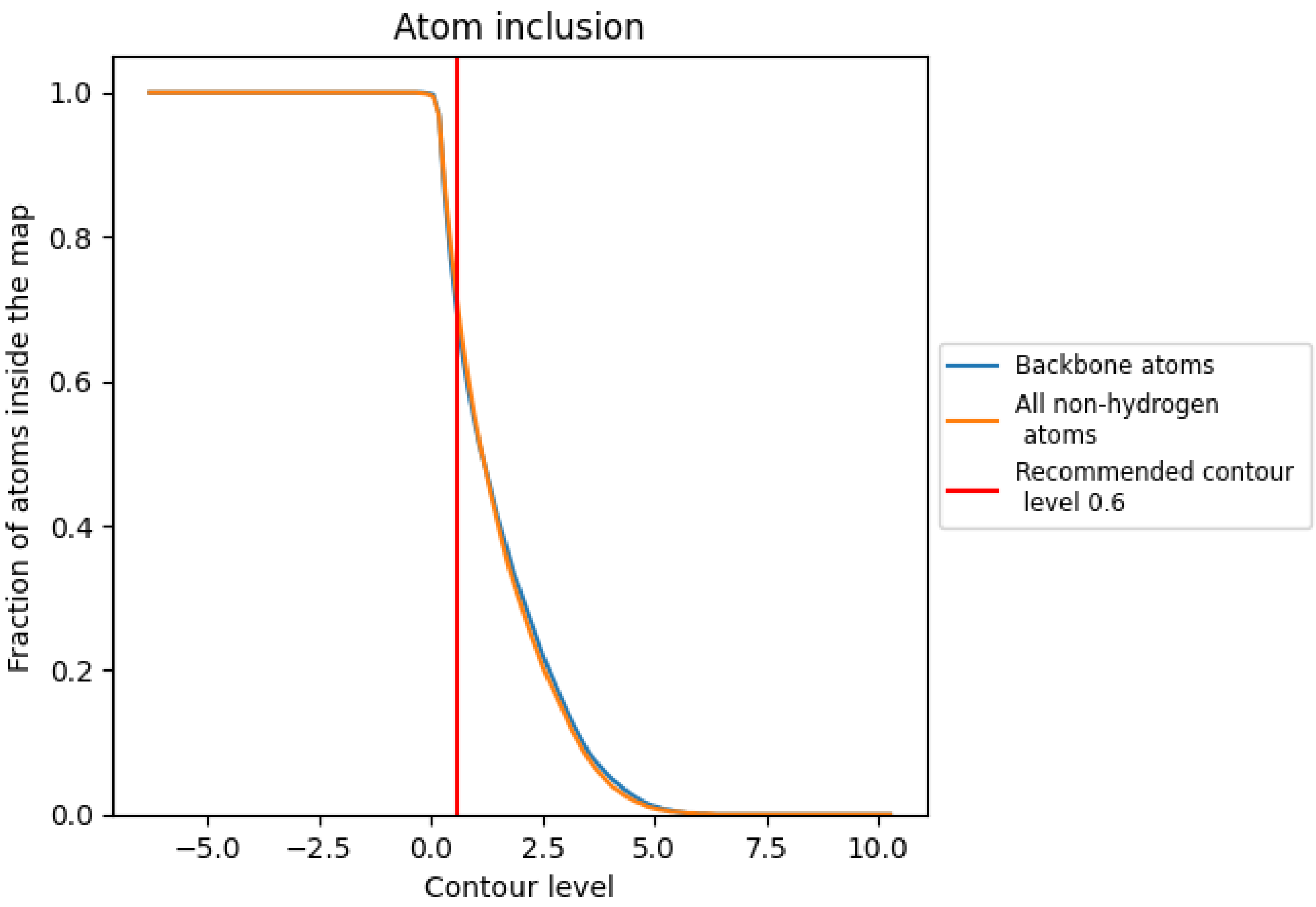

At the recommended contour level, 69% of all backbone atoms, 70% of all non-hydrogen atoms, are inside the map.

For Manuscript Review

9.5 Map-model fit summary (i)

The table lists the average atom inclusion at the recommended contour level (0.6) and Q-score for the entire model and for each chain.

Chain	Atom inclusion	Q-score
All	0.7050	0.5320
A	0.7500	0.5420
B	0.7500	0.5410
C	0.6200	0.5220
D	0.6220	0.5240
E	0.3150	0.4390
F	0.7510	0.5420
G	0.6170	0.5250
H	0.3060	0.4340
I	0.1070	0.3590
J	0.1030	0.3410
K	0.1070	0.3540
L	0.3090	0.4350
M	0.1030	0.3430
N	0.1070	0.3660
O	0.1030	0.3460

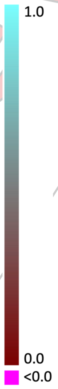

For Manuscript Review
