# Supplementary material for: Design of customized coronavirus receptors
Source: Nature. Author manuscript; Available in PMC 2025 Jun 24. (PMC12187079; doi:10.1038/s41586-024-08121-5)
Supplement: PDB Validation Report 9C45 [file NIHMS2083933-supplement-PDB_Validation_Report_9C45.pdf]

Full wwPDB EM Validation Report (i)

Jun 4, 2024 – 11:06 AM EDT

PDB ID	:	9C45
EMDB ID	:	EMD-45175
Title	:	SARS-CoV-2 S + S2L20 (local refinement of NTD and S2L20 Fab variable region)
Deposited on	:	2024-06-03
Resolution	:	3.20 Å(reported)

This wwPDB validation report is for manuscript review

For Manuscript Review

This is a Full wwPDB EM Validation Report.

This report is produced by the wwPDB biocuration pipeline after annotation of the structure.

We welcome your comments at *validation@mail.wwpdb.org*
A user guide is available at
https://www.wwpdb.org/validation/2017/EMValidationReportHelp
with specific help available everywhere you see the (i) symbol.

The types of validation reports are described at
http://www.wwpdb.org/validation/2017/FAQs#types.

The following versions of software and data (see references (i)) were used in the production of this report:

EMDB validation analysis	:	0.0.1.dev92
Mogul	:	1.8.5 (274361), CSD as541be (2020)
MolProbity	:	4.02b-467
Percentile statistics	:	20191225.v01 (using entries in the PDB archive December 25th 2019)
MapQ	:	1.9.13
Ideal geometry (proteins)	:	Engh & Huber (2001)
Ideal geometry (DNA, RNA)	:	Parkinson et al. (1996)
Validation Pipeline (wwPDB-VP)	:	2.36.2

1 Overall quality at a glance (i)

The following experimental techniques were used to determine the structure:
ELECTRON MICROSCOPY

The reported resolution of this entry is 3.20 Å.

Percentile scores (ranging between 0-100) for global validation metrics of the entry are shown in the following graphic. The table shows the number of entries on which the scores are based.

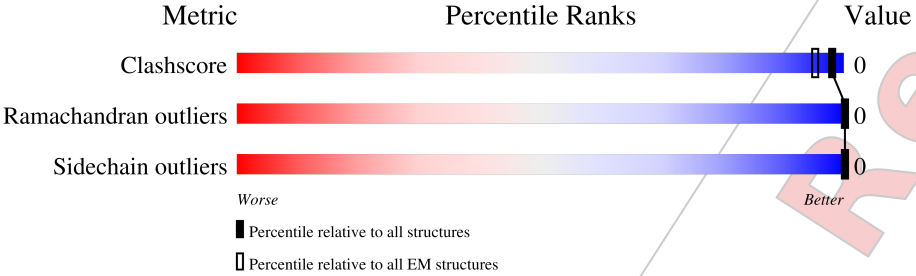

Metric	Whole archive (#Entries)	EM structures (#Entries)
Clashscore	158937	4297
Ramachandran outliers	154571	4023
Sidechain outliers	154315	3826

The table below summarises the geometric issues observed across the polymeric chains and their fit to the map. The red, orange, yellow and green segments of the bar indicate the fraction of residues that contain outliers for >=3, 2, 1 and 0 types of geometric quality criteria respectively. A grey segment represents the fraction of residues that are not modelled. The numeric value for each fraction is indicated below the corresponding segment, with a dot representing fractions <=5% The upper red bar (where present) indicates the fraction of residues that have poor fit to the EM map (all-atom inclusion < 40%). The numeric value is given above the bar.

Mol	Chain	Length	Quality of chain
1	L	107	7% / 100%
2	H	121	• / 100%
3	A	1288	• / 21% 79%
4	B	2	50% / 100%
5	C	3	67% / 33% 67%

For Manuscript Review

2 Entry composition (i)

There are 6 unique types of molecules in this entry. The entry contains 4006 atoms, of which 0 are hydrogens and 0 are deuteriums.

In the tables below, the AltConf column contains the number of residues with at least one atom in alternate conformation and the Trace column contains the number of residues modelled with at most 2 atoms.

- Molecule 1 is a protein called S2L20 Fab Light Chain Variable Region.

Mol	Chain	Residues	Atoms					AltConf	Trace
			Total	C	N	O	S		
1	L	107	830	527	134	165	4	0	0

- Molecule 2 is a protein called S2L20 Fab Heavy Chain Variable.

Mol	Chain	Residues	Atoms					AltConf	Trace
			Total	C	N	O	S		
2	H	121	948	600	163	181	4	0	0

- Molecule 3 is a protein called Spike glycoprotein.

Mol	Chain	Residues	Atoms					AltConf	Trace
			Total	C	N	O	S		
3	A	268	2105	1370	342	386	7	0	0

There are 89 discrepancies between the modelled and reference sequences:

Chain	Residue	Modelled	Actual	Comment	Reference
A	682	GLY	ARG	engineered mutation	UNP P0DTC2
A	683	SER	ARG	engineered mutation	UNP P0DTC2
A	685	SER	ARG	engineered mutation	UNP P0DTC2
A	817	PRO	PHE	engineered mutation	UNP P0DTC2
A	892	PRO	ALA	engineered mutation	UNP P0DTC2
A	899	PRO	ALA	engineered mutation	UNP P0DTC2
A	942	PRO	ALA	engineered mutation	UNP P0DTC2
A	986	PRO	LYS	engineered mutation	UNP P0DTC2
A	987	PRO	VAL	engineered mutation	UNP P0DTC2
A	1209	GLY	-	expression tag	UNP P0DTC2
A	1210	SER	-	expression tag	UNP P0DTC2
A	1211	GLY	-	expression tag	UNP P0DTC2
A	1212	TYR	-	expression tag	UNP P0DTC2
A	1213	ILE	-	expression tag	UNP P0DTC2
A	1214	PRO	-	expression tag	UNP P0DTC2

Continued on next page...

For Manuscript Review

Continued from previous page...

Chain	Residue	Modelled	Actual	Comment	Reference
A	1215	GLU	-	expression tag	UNP P0DTC2
A	1216	ALA	-	expression tag	UNP P0DTC2
A	1217	PRO	-	expression tag	UNP P0DTC2
A	1218	ARG	-	expression tag	UNP P0DTC2
A	1219	ASP	-	expression tag	UNP P0DTC2
A	1220	GLY	-	expression tag	UNP P0DTC2
A	1221	GLN	-	expression tag	UNP P0DTC2
A	1222	ALA	-	expression tag	UNP P0DTC2
A	1223	TYR	-	expression tag	UNP P0DTC2
A	1224	VAL	-	expression tag	UNP P0DTC2
A	1225	ARG	-	expression tag	UNP P0DTC2
A	1226	LYS	-	expression tag	UNP P0DTC2
A	1227	ASP	-	expression tag	UNP P0DTC2
A	1228	GLY	-	expression tag	UNP P0DTC2
A	1229	GLU	-	expression tag	UNP P0DTC2
A	1230	TRP	-	expression tag	UNP P0DTC2
A	1231	VAL	-	expression tag	UNP P0DTC2
A	1232	LEU	-	expression tag	UNP P0DTC2
A	1233	LEU	-	expression tag	UNP P0DTC2
A	1234	SER	-	expression tag	UNP P0DTC2
A	1235	THR	-	expression tag	UNP P0DTC2
A	1236	PHE	-	expression tag	UNP P0DTC2
A	1237	LEU	-	expression tag	UNP P0DTC2
A	1238	GLY	-	expression tag	UNP P0DTC2
A	1239	ARG	-	expression tag	UNP P0DTC2
A	1240	SER	-	expression tag	UNP P0DTC2
A	1241	LEU	-	expression tag	UNP P0DTC2
A	1242	GLU	-	expression tag	UNP P0DTC2
A	1243	VAL	-	expression tag	UNP P0DTC2
A	1244	LEU	-	expression tag	UNP P0DTC2
A	1245	PHE	-	expression tag	UNP P0DTC2
A	1246	GLN	-	expression tag	UNP P0DTC2
A	1247	GLY	-	expression tag	UNP P0DTC2
A	1248	PRO	-	expression tag	UNP P0DTC2
A	1249	GLY	-	expression tag	UNP P0DTC2
A	1250	HIS	-	expression tag	UNP P0DTC2
A	1251	HIS	-	expression tag	UNP P0DTC2
A	1252	HIS	-	expression tag	UNP P0DTC2
A	1253	HIS	-	expression tag	UNP P0DTC2
A	1254	HIS	-	expression tag	UNP P0DTC2
A	1255	HIS	-	expression tag	UNP P0DTC2
A	1256	HIS	-	expression tag	UNP P0DTC2

Continued on next page...

For Manuscript Review

Continued from previous page...

Chain	Residue	Modelled	Actual	Comment	Reference
A	1257	HIS	-	expression tag	UNP P0DTC2
A	1258	SER	-	expression tag	UNP P0DTC2
A	1259	ALA	-	expression tag	UNP P0DTC2
A	1260	TRP	-	expression tag	UNP P0DTC2
A	1261	SER	-	expression tag	UNP P0DTC2
A	1262	HIS	-	expression tag	UNP P0DTC2
A	1263	PRO	-	expression tag	UNP P0DTC2
A	1264	GLN	-	expression tag	UNP P0DTC2
A	1265	PHE	-	expression tag	UNP P0DTC2
A	1266	GLU	-	expression tag	UNP P0DTC2
A	1267	LYS	-	expression tag	UNP P0DTC2
A	1268	GLY	-	expression tag	UNP P0DTC2
A	1269	GLY	-	expression tag	UNP P0DTC2
A	1270	GLY	-	expression tag	UNP P0DTC2
A	1271	SER	-	expression tag	UNP P0DTC2
A	1272	GLY	-	expression tag	UNP P0DTC2
A	1273	GLY	-	expression tag	UNP P0DTC2
A	1274	GLY	-	expression tag	UNP P0DTC2
A	1275	GLY	-	expression tag	UNP P0DTC2
A	1276	SER	-	expression tag	UNP P0DTC2
A	1277	GLY	-	expression tag	UNP P0DTC2
A	1278	GLY	-	expression tag	UNP P0DTC2
A	1279	SER	-	expression tag	UNP P0DTC2
A	1280	ALA	-	expression tag	UNP P0DTC2
A	1281	TRP	-	expression tag	UNP P0DTC2
A	1282	SER	-	expression tag	UNP P0DTC2
A	1283	HIS	-	expression tag	UNP P0DTC2
A	1284	PRO	-	expression tag	UNP P0DTC2
A	1285	GLN	-	expression tag	UNP P0DTC2
A	1286	PHE	-	expression tag	UNP P0DTC2
A	1287	GLU	-	expression tag	UNP P0DTC2
A	1288	LYS	-	expression tag	UNP P0DTC2

- Molecule 4 is an oligosaccharide called 2-acetamido-2-deoxy-beta-D-glucopyranose-(1-4)-2-acetamido-2-deoxy-beta-D-glucopyranose.

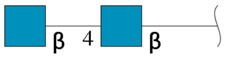

For Manuscript Review

Mol	Chain	Residues	Atoms	AltConf	Trace
4	B	2	Total C N O<br>28 16 2 10	0	0

- Molecule 5 is an oligosaccharide called beta-D-mannopyranose-(1-4)-2-acetamido-2-deoxy-beta-D-glucopyranose-(1-4)-2-acetamido-2-deoxy-beta-D-glucopyranose.

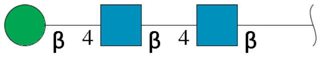

Mol	Chain	Residues	Atoms	AltConf	Trace
5	C	3	Total C N O<br>39 22 2 15	0	0

- Molecule 6 is 2-acetamido-2-deoxy-beta-D-glucopyranose (three-letter code: NAG) (formula: $C_8H_{15}NO_6$).

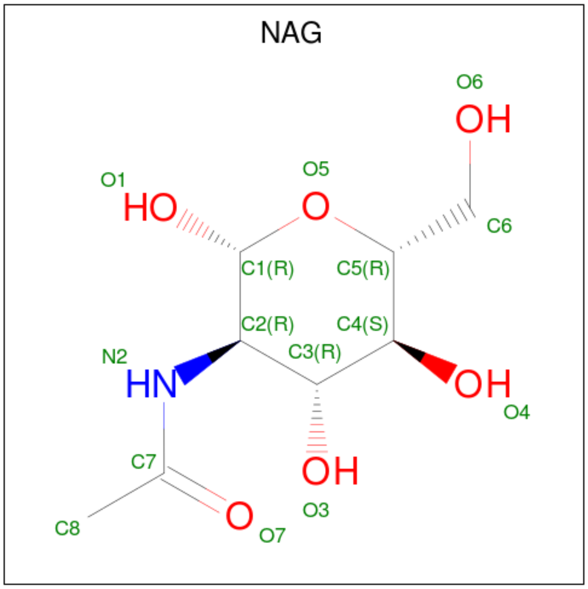

Mol	Chain	Residues	Atoms	AltConf
6	A	1	Total C N O<br>14 8 1 5	0
6	A	1	Total C N O<br>14 8 1 5	0
6	A	1	Total C N O<br>14 8 1 5	0
6	A	1	Total C N O<br>14 8 1 5	0

For Manuscript Review

3 Residue-property plots ⓘ

These plots are drawn for all protein, RNA, DNA and oligosaccharide chains in the entry. The first graphic for a chain summarises the proportions of the various outlier classes displayed in the second graphic. The second graphic shows the sequence view annotated by issues in geometry and atom inclusion in map density. Residues are color-coded according to the number of geometric quality criteria for which they contain at least one outlier: green = 0, yellow = 1, orange = 2 and red = 3 or more. A red diamond above a residue indicates a poor fit to the EM map for this residue (all-atom inclusion < 40%). Stretches of 2 or more consecutive residues without any outlier are shown as a green connector. Residues present in the sample, but not in the model, are shown in grey.

- Molecule 1: S2L20 Fab Light Chain Variable Region

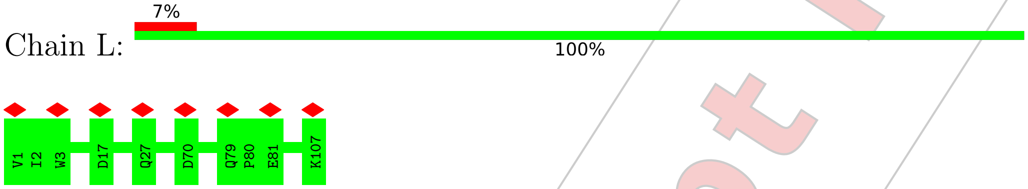

- Molecule 2: S2L20 Fab Heavy Chain Variable

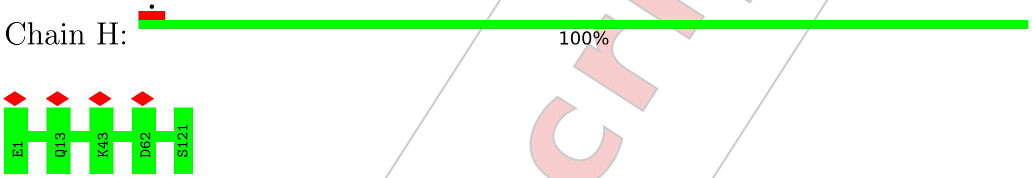

- Molecule 3: Spike glycoprotein

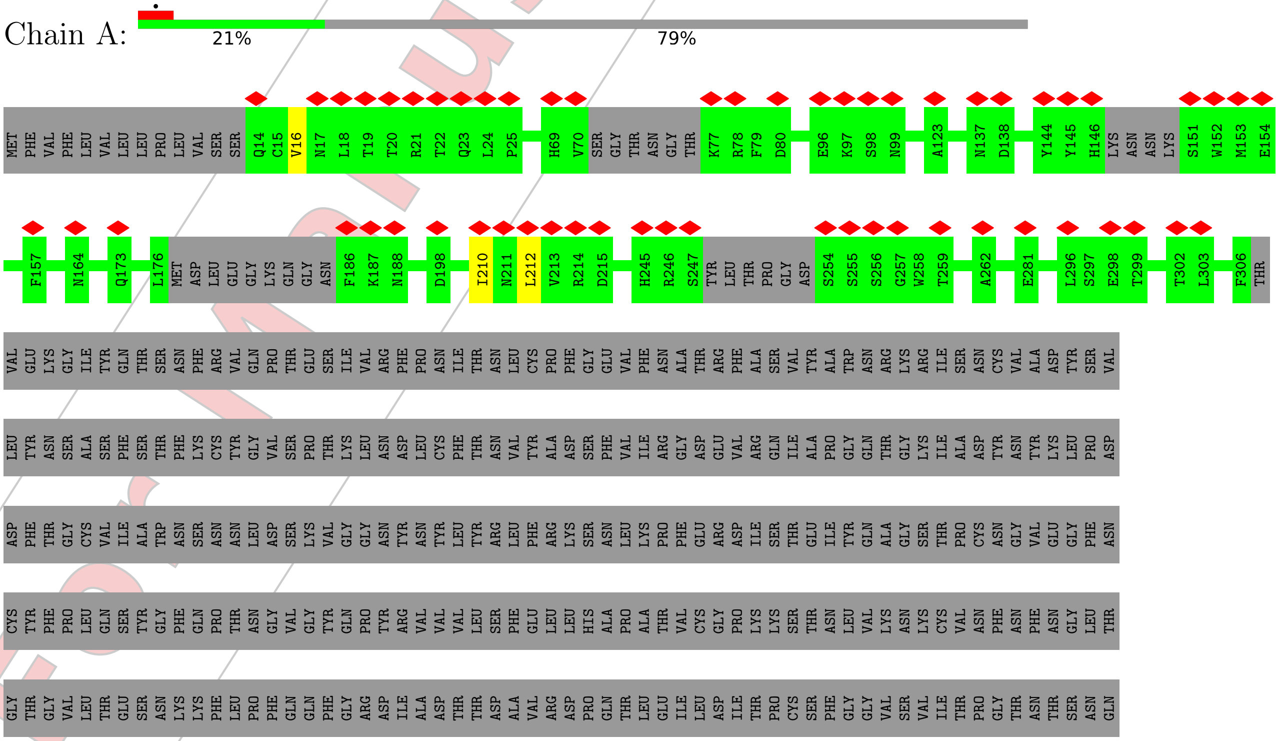

For Manuscript Review

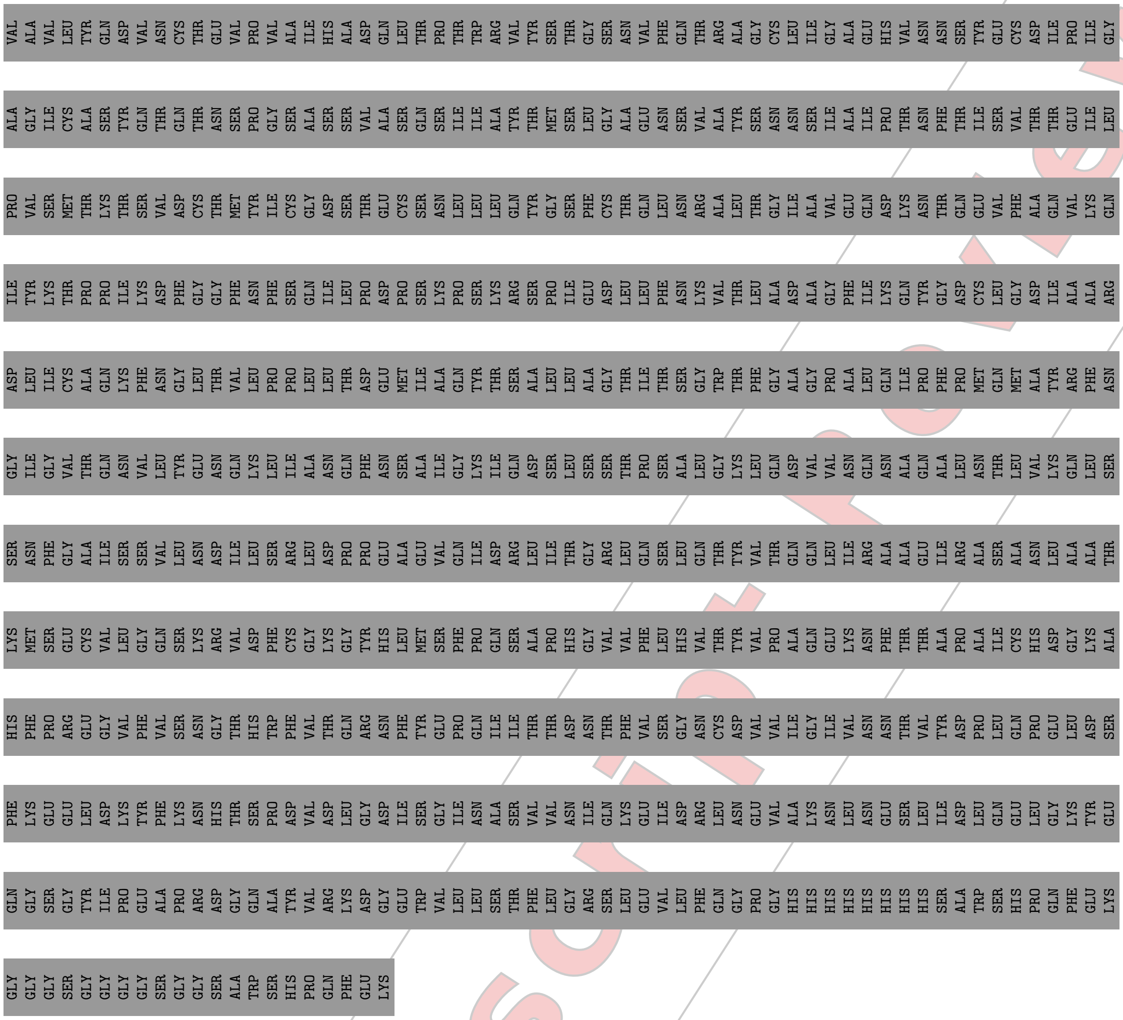

• Molecule 4: 2-acetamido-2-deoxy-beta-D-glucopyranose-(1-4)-2-acetamido-2-deoxy-beta-D-glucopyranose

Chain B:

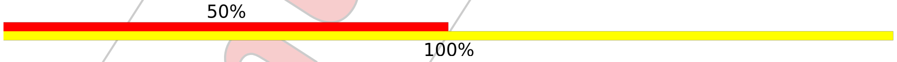

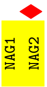

• Molecule 5: beta-D-mannopyranose-(1-4)-2-acetamido-2-deoxy-beta-D-glucopyranose-(1-4)-2-acetamido-2-deoxy-beta-D-glucopyranose

Chain C:

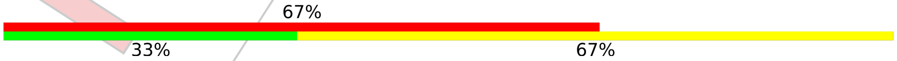

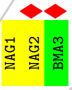

For Manuscript Review

4 Experimental information (i)

Property	Value	Source
EM reconstruction method	SINGLE PARTICLE	Depositor
Imposed symmetry	POINT, Not provided	
Number of particles used	209164	Depositor
Resolution determination method	FSC 0.143 CUT-OFF	Depositor
CTF correction method	PHASE FLIPPING AND AMPLITUDE CORRECTION	Depositor
Microscope	FEI TITAN KRIOS	Depositor
Voltage (kV)	300	Depositor
Electron dose ($e^-/\text{Å}^2$)	63	Depositor
Minimum defocus (nm)	5000	Depositor
Maximum defocus (nm)	20000	Depositor
Magnification	Not provided	
Image detector	GATAN K3 (6k x 4k)	Depositor
Maximum map value	9.598	Depositor
Minimum map value	-6.545	Depositor
Average map value	-0.000	Depositor
Map value standard deviation	0.085	Depositor
Recommended contour level	1.4	Depositor
Map size (Å)	431.616, 431.616, 431.616	wwPDB
Map dimensions	512, 512, 512	wwPDB
Map angles (°)	90.0, 90.0, 90.0	wwPDB
Pixel spacing (Å)	0.843, 0.843, 0.843	Depositor

For Manuscript Review

5 Model quality (i)

5.1 Standard geometry (i)

Bond lengths and bond angles in the following residue types are not validated in this section: NAG, BMA

The Z score for a bond length (or angle) is the number of standard deviations the observed value is removed from the expected value. A bond length (or angle) with $|Z| > 5$ is considered an outlier worth inspection. RMSZ is the root-mean-square of all Z scores of the bond lengths (or angles).

Mol	Chain	Bond lengths		Bond angles	
		RMSZ	#$|Z|$ >5	RMSZ	#$|Z|$ >5
1	L	0.60	0/852	0.54	0/1158
2	H	0.66	0/971	0.60	0/1312
3	A	0.62	0/2164	0.55	0/2953
All	All	0.62	0/3987	0.56	0/5423

There are no bond length outliers.

There are no bond angle outliers.

There are no chirality outliers.

There are no planarity outliers.

5.2 Too-close contacts (i)

In the following table, the Non-H and H(model) columns list the number of non-hydrogen atoms and hydrogen atoms in the chain respectively. The H(added) column lists the number of hydrogen atoms added and optimized by MolProbity. The Clashes column lists the number of clashes within the asymmetric unit, whereas Symm-Clashes lists symmetry-related clashes.

Mol	Chain	Non-H	H(model)	H(added)	Clashes	Symm-Clashes
1	L	830	0	790	0	0
2	H	948	0	904	0	0
3	A	2105	0	1960	2	0
4	B	28	0	25	0	0
5	C	39	0	34	0	0
6	A	56	0	52	1	0
All	All	4006	0	3765	2	0

The all-atom clashscore is defined as the number of clashes found per 1000 atoms (including hydrogen atoms). The all-atom clashscore for this structure is 0.

For Manuscript Review

All (2) close contacts within the same asymmetric unit are listed below, sorted by their clash magnitude.

Atom-1	Atom-2	Interatomic distance (Å)	Clash overlap (Å)
3:A:16:VAL:HA	6:A:1302:NAG:H82	2.00	0.44
3:A:210:ILE:HG12	3:A:212:LEU:H	1.85	0.42

There are no symmetry-related clashes.

5.3 Torsion angles (i)

5.3.1 Protein backbone (i)

In the following table, the Percentiles column shows the percent Ramachandran outliers of the chain as a percentile score with respect to all PDB entries followed by that with respect to all EM entries.

The Analysed column shows the number of residues for which the backbone conformation was analysed, and the total number of residues.

Mol	Chain	Analysed	Favoured	Allowed	Outliers	Percentiles	
1	L	105/107 (98%)	101 (96%)	4 (4%)	0	100	100
2	H	119/121 (98%)	118 (99%)	1 (1%)	0	100	100
3	A	258/1288 (20%)	249 (96%)	9 (4%)	0	100	100
All	All	482/1516 (32%)	468 (97%)	14 (3%)	0	100	100

There are no Ramachandran outliers to report.

5.3.2 Protein sidechains (i)

In the following table, the Percentiles column shows the percent sidechain outliers of the chain as a percentile score with respect to all PDB entries followed by that with respect to all EM entries.

The Analysed column shows the number of residues for which the sidechain conformation was analysed, and the total number of residues.

Mol	Chain	Analysed	Rotameric	Outliers	Percentiles	
1	L	93/94 (99%)	93 (100%)	0	100	100
2	H	98/98 (100%)	98 (100%)	0	100	100
3	A	224/1116 (20%)	224 (100%)	0	100	100

Continued on next page...

For Manuscript Review

Continued from previous page...

Mol	Chain	Analysed	Rotameric	Outliers	Percentiles	
All	All	415/1308 (32%)	415 (100%)	0	100	100

There are no protein residues with a non-rotameric sidechain to report.

Sometimes sidechains can be flipped to improve hydrogen bonding and reduce clashes. All (1) such sidechains are listed below:

Mol	Chain	Res	Type
1	L	37	GLN

5.3.3 RNA (i)

There are no RNA molecules in this entry.

5.4 Non-standard residues in protein, DNA, RNA chains (i)

There are no non-standard protein/DNA/RNA residues in this entry.

5.5 Carbohydrates (i)

5 monosaccharides are modelled in this entry.

In the following table, the Counts columns list the number of bonds (or angles) for which Mogul statistics could be retrieved, the number of bonds (or angles) that are observed in the model and the number of bonds (or angles) that are defined in the Chemical Component Dictionary. The Link column lists molecule types, if any, to which the group is linked. The Z score for a bond length (or angle) is the number of standard deviations the observed value is removed from the expected value. A bond length (or angle) with $|Z| > 2$ is considered an outlier worth inspection. RMSZ is the root-mean-square of all Z scores of the bond lengths (or angles).

Mol	Type	Chain	Res	Link	Bond lengths			Bond angles		
					Counts	RMSZ	$\#\|Z\| > 2$	Counts	RMSZ	$\#\|Z\| > 2$
4	NAG	B	1	3,4	14,14,15	1.12	1 (7%)	17,19,21	1.10	1 (5%)
4	NAG	B	2	4	14,14,15	1.09	1 (7%)	17,19,21	0.99	1 (5%)
5	NAG	C	1	5,3	14,14,15	1.17	1 (7%)	17,19,21	0.99	1 (5%)
5	NAG	C	2	5	14,14,15	1.01	1 (7%)	17,19,21	1.01	1 (5%)
5	BMA	C	3	5	11,11,12	1.04	0	15,15,17	0.58	0

In the following table, the Chirals column lists the number of chiral outliers, the number of chiral centers analysed, the number of these observed in the model and the number defined in the Chemical Component Dictionary. Similar counts are reported in the Torsion and Rings columns.

For Manuscript Review

'-' means no outliers of that kind were identified.

Mol	Type	Chain	Res	Link	Chirals	Torsions	Rings
4	NAG	B	1	3,4	-	0/6/23/26	0/1/1/1
4	NAG	B	2	4	-	0/6/23/26	0/1/1/1
5	NAG	C	1	5,3	-	0/6/23/26	0/1/1/1
5	NAG	C	2	5	-	0/6/23/26	0/1/1/1
5	BMA	C	3	5	-	0/2/19/22	0/1/1/1

All (4) bond length outliers are listed below:

Mol	Chain	Res	Type	Atoms	Z	Observed(Å)	Ideal(Å)
5	C	1	NAG	C1-C2	3.31	1.57	1.52
4	B	1	NAG	C1-C2	3.11	1.57	1.52
4	B	2	NAG	C1-C2	2.91	1.56	1.52
5	C	2	NAG	C1-C2	2.58	1.56	1.52

All (4) bond angle outliers are listed below:

Mol	Chain	Res	Type	Atoms	Z	Observed(^{o})	Ideal(^{o})
4	B	1	NAG	C8-C7-N2	2.64	120.57	116.10
4	B	2	NAG	C8-C7-N2	2.53	120.38	116.10
5	C	2	NAG	C8-C7-N2	2.35	120.09	116.10
5	C	1	NAG	C8-C7-N2	2.34	120.06	116.10

There are no chirality outliers.

There are no torsion outliers.

There are no ring outliers.

No monomer is involved in short contacts.

The following is a two-dimensional graphical depiction of Mogul quality analysis of bond lengths, bond angles, torsion angles, and ring geometry for oligosaccharide.

For Manuscript Review

Oligosaccharide Chain B	
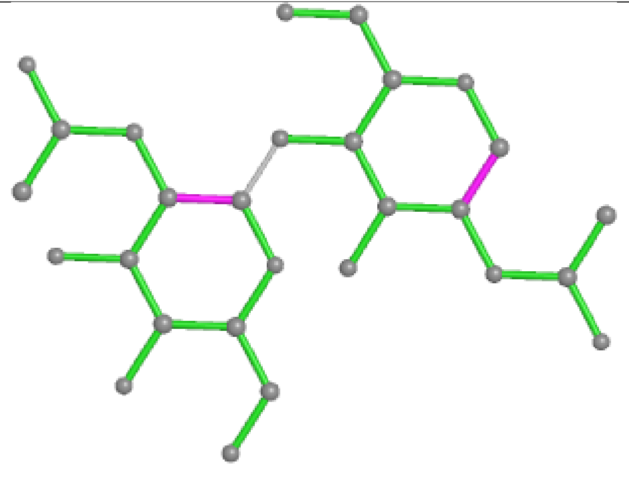 Bond lengths	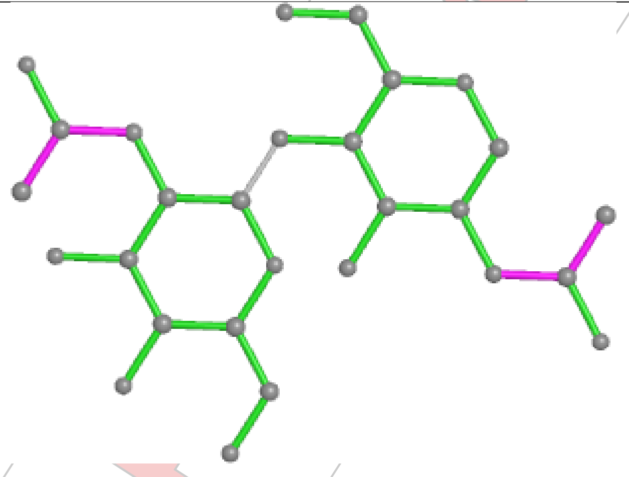 Bond angles
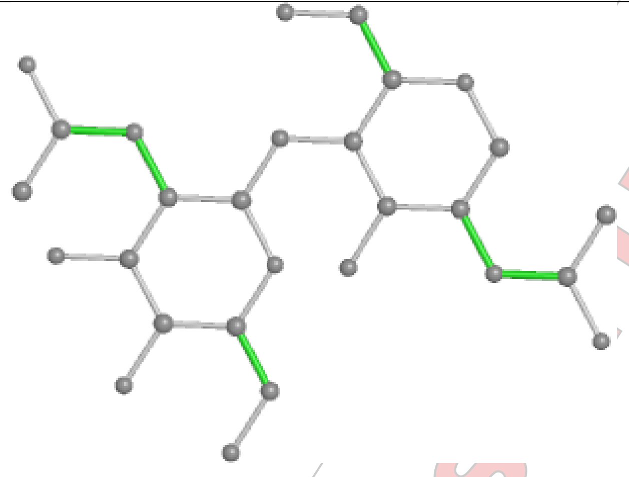 Torsions	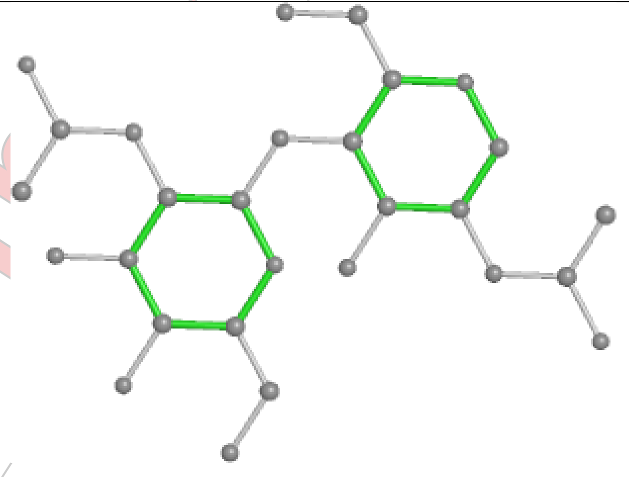 Rings

For Manuscript Review

Oligosaccharide Chain C

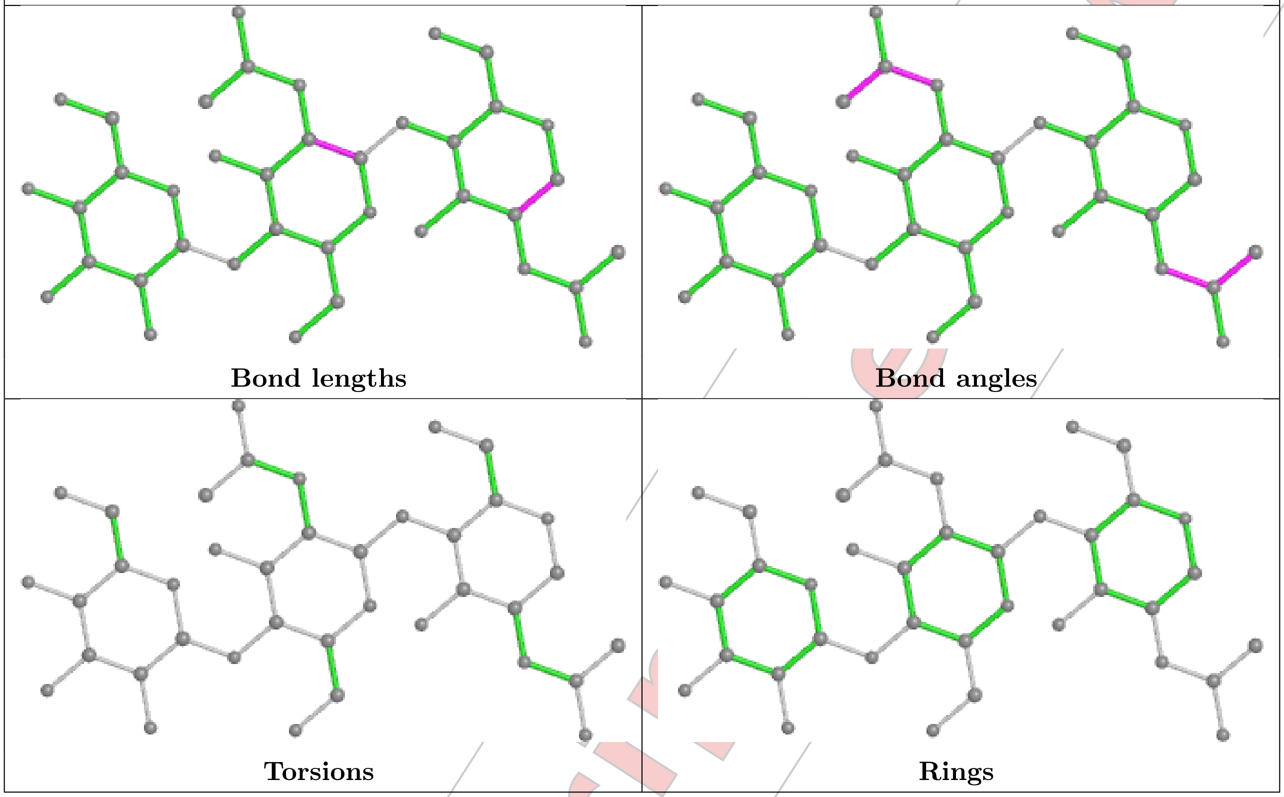

5.6 Ligand geometry (i)

4 ligands are modelled in this entry.

In the following table, the Counts columns list the number of bonds (or angles) for which Mogul statistics could be retrieved, the number of bonds (or angles) that are observed in the model and the number of bonds (or angles) that are defined in the Chemical Component Dictionary. The Link column lists molecule types, if any, to which the group is linked. The Z score for a bond length (or angle) is the number of standard deviations the observed value is removed from the expected value. A bond length (or angle) with $|Z| > 2$ is considered an outlier worth inspection. RMSZ is the root-mean-square of all Z scores of the bond lengths (or angles).

Mol	Type	Chain	Res	Link	Bond lengths			Bond angles		
					Counts	RMSZ	$\#\|Z\| > 2$	Counts	RMSZ	$\#\|Z\| > 2$
6	NAG	A	1302	3	14,14,15	1.09	1 (7%)	17,19,21	1.07	1 (5%)
6	NAG	A	1301	3	14,14,15	1.09	1 (7%)	17,19,21	0.99	1 (5%)
6	NAG	A	1303	3	14,14,15	1.14	1 (7%)	17,19,21	1.04	1 (5%)
6	NAG	A	1304	3	14,14,15	1.17	1 (7%)	17,19,21	1.02	1 (5%)

In the following table, the Chirals column lists the number of chiral outliers, the number of chiral centers analysed, the number of these observed in the model and the number defined in the Chemical Component Dictionary. Similar counts are reported in the Torsion and Rings columns. '-' means no outliers of that kind were identified.

For Manuscript Review

Mol	Type	Chain	Res	Link	Chirals	Torsions	Rings
6	NAG	A	1302	3	-	1/6/23/26	0/1/1/1
6	NAG	A	1301	3	-	0/6/23/26	0/1/1/1
6	NAG	A	1303	3	-	0/6/23/26	0/1/1/1
6	NAG	A	1304	3	-	0/6/23/26	0/1/1/1

All (4) bond length outliers are listed below:

Mol	Chain	Res	Type	Atoms	Z	Observed(Å)	Ideal(Å)
6	A	1304	NAG	C1-C2	3.43	1.57	1.52
6	A	1303	NAG	C1-C2	3.32	1.57	1.52
6	A	1301	NAG	C1-C2	3.13	1.57	1.52
6	A	1302	NAG	C1-C2	3.03	1.56	1.52

All (4) bond angle outliers are listed below:

Mol	Chain	Res	Type	Atoms	Z	Observed(^{o})	Ideal(^{o})
6	A	1302	NAG	C8-C7-N2	2.65	120.58	116.10
6	A	1301	NAG	C8-C7-N2	2.54	120.40	116.10
6	A	1303	NAG	C8-C7-N2	2.40	120.17	116.10
6	A	1304	NAG	C8-C7-N2	2.40	120.16	116.10

There are no chirality outliers.

All (1) torsion outliers are listed below:

Mol	Chain	Res	Type	Atoms
6	A	1302	NAG	C4-C5-C6-O6

There are no ring outliers.

1 monomer is involved in 1 short contact:

Mol	Chain	Res	Type	Clashes	Symm-Clashes
6	A	1302	NAG	1	0

5.7 Other polymers (i)

There are no such residues in this entry.

5.8 Polymer linkage issues (i)

There are no chain breaks in this entry.

For Manuscript Review

6 Map visualisation (i)

This section contains visualisations of the EMDB entry EMD-45175. These allow visual inspection of the internal detail of the map and identification of artifacts.

Images derived from a raw map, generated by summing the deposited half-maps, are presented below the corresponding image components of the primary map to allow further visual inspection and comparison with those of the primary map.

6.1 Orthogonal projections (i)

6.1.1 Primary map

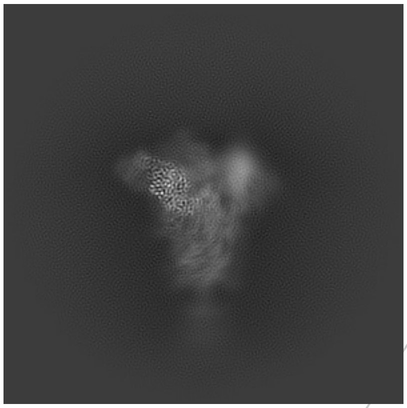

X

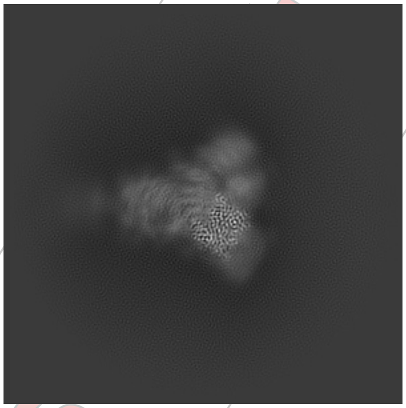

Y

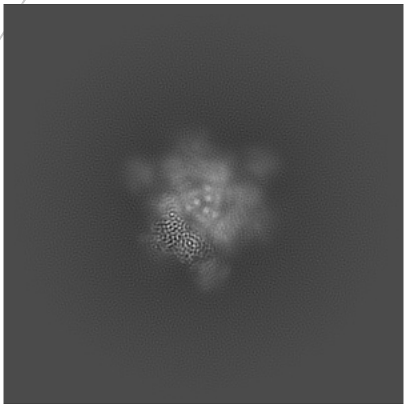

Z

6.1.2 Raw map

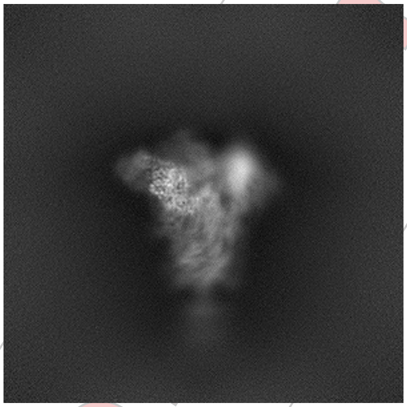

X

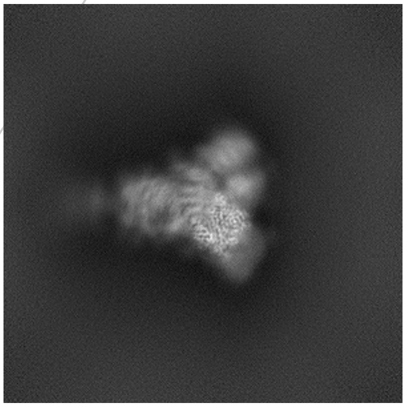

Y

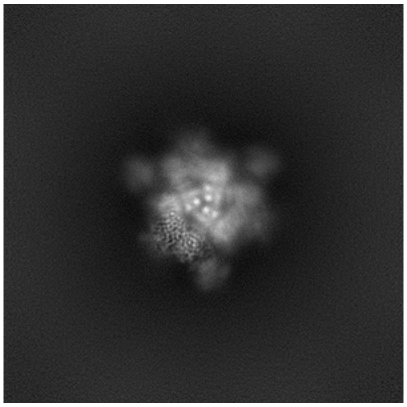

Z

The images above show the map projected in three orthogonal directions.

For Manuscript Review

6.2 Central slices (i)

6.2.1 Primary map

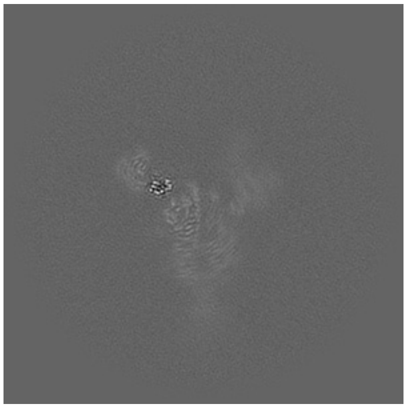
X Index: 256

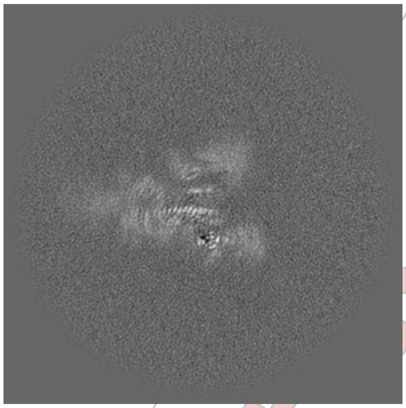
Y Index: 256

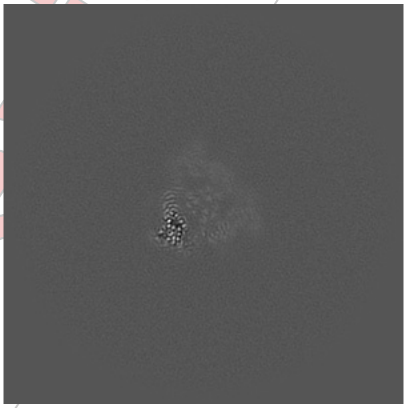
Z Index: 256

6.2.2 Raw map

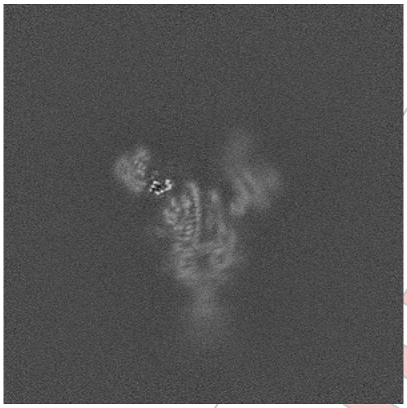
X Index: 256

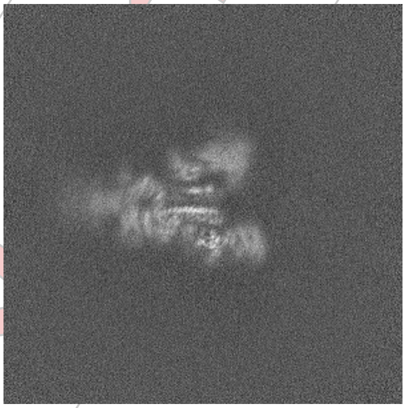
Y Index: 256

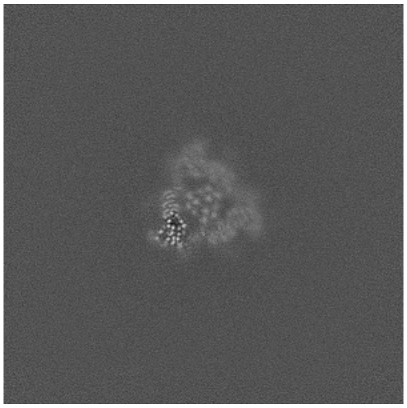
Z Index: 256

The images above show central slices of the map in three orthogonal directions.

For Manuscript Review

6.3 Largest variance slices (i)

6.3.1 Primary map

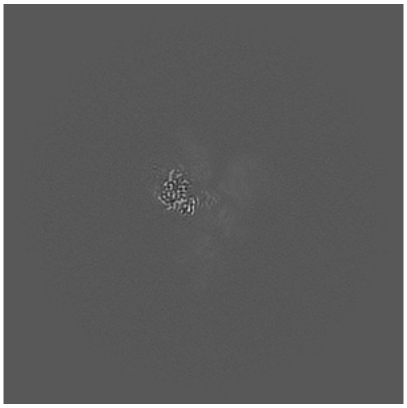
X Index: 216

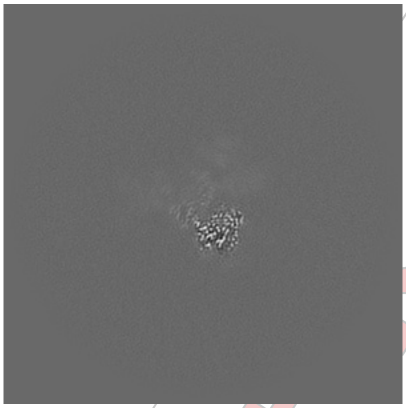
Y Index: 217

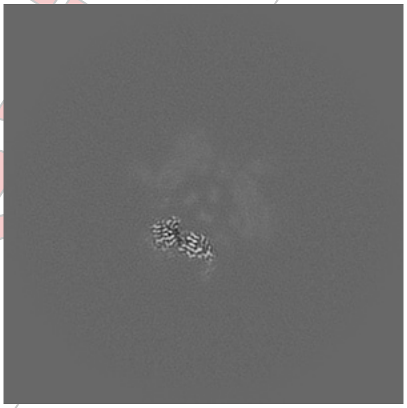
Z Index: 279

6.3.2 Raw map

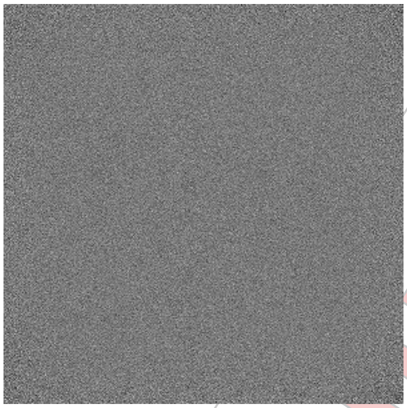
X Index: 0

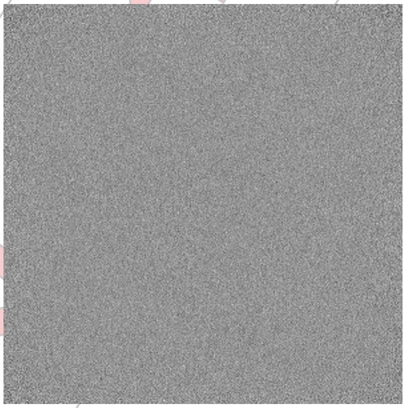
Y Index: 0

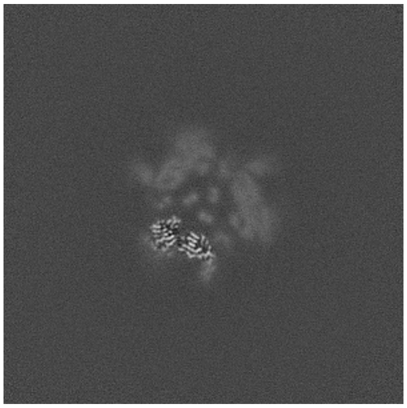
Z Index: 279

The images above show the largest variance slices of the map in three orthogonal directions.

For Manuscript Review

6.4 Orthogonal standard-deviation projections (False-color) (i)

6.4.1 Primary map

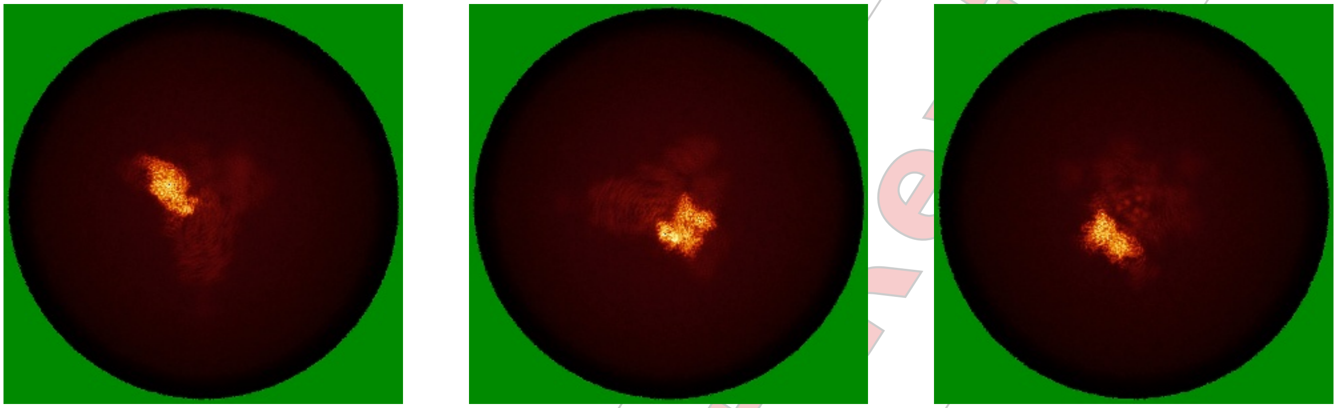

X Y Z

6.4.2 Raw map

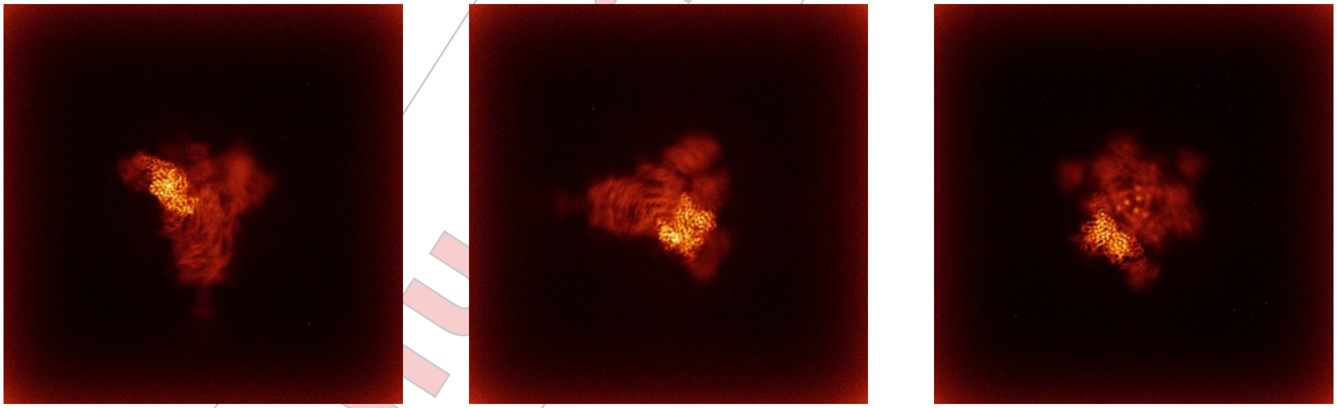

X Y Z

The images above show the map standard deviation projections with false color in three orthogonal directions. Minimum values are shown in green, max in blue, and dark to light orange shades represent small to large values respectively.

For Manuscript Review

6.5 Orthogonal surface views (i)

6.5.1 Primary map

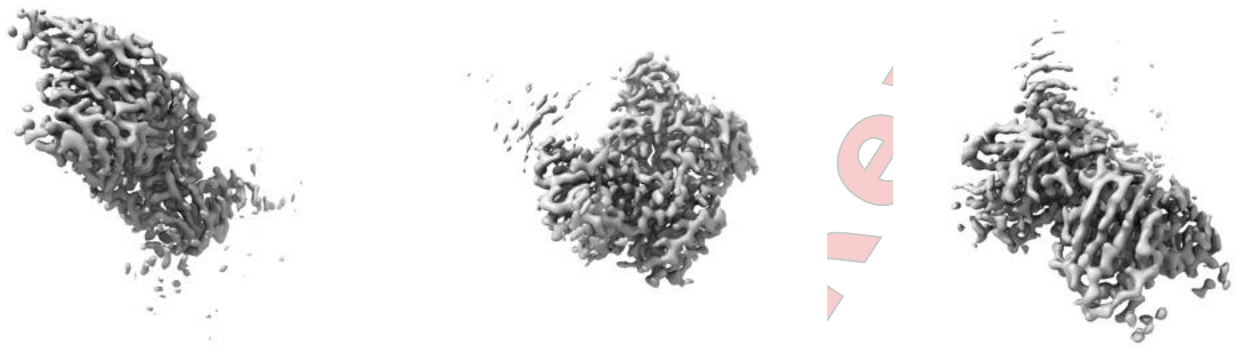

X Y Z

The images above show the 3D surface view of the map at the recommended contour level 1.4. These images, in conjunction with the slice images, may facilitate assessment of whether an appropriate contour level has been provided.

6.5.2 Raw map

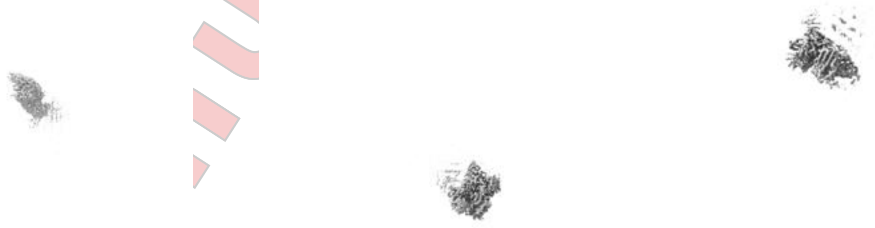

X Y Z

These images show the 3D surface of the raw map. The raw map's contour level was selected so that its surface encloses the same volume as the primary map does at its recommended contour level.

6.6 Mask visualisation (i)

This section was not generated. No masks/segmentation were deposited.

For Manuscript Review

7 Map analysis (i)

This section contains the results of statistical analysis of the map.

7.1 Map-value distribution (i)

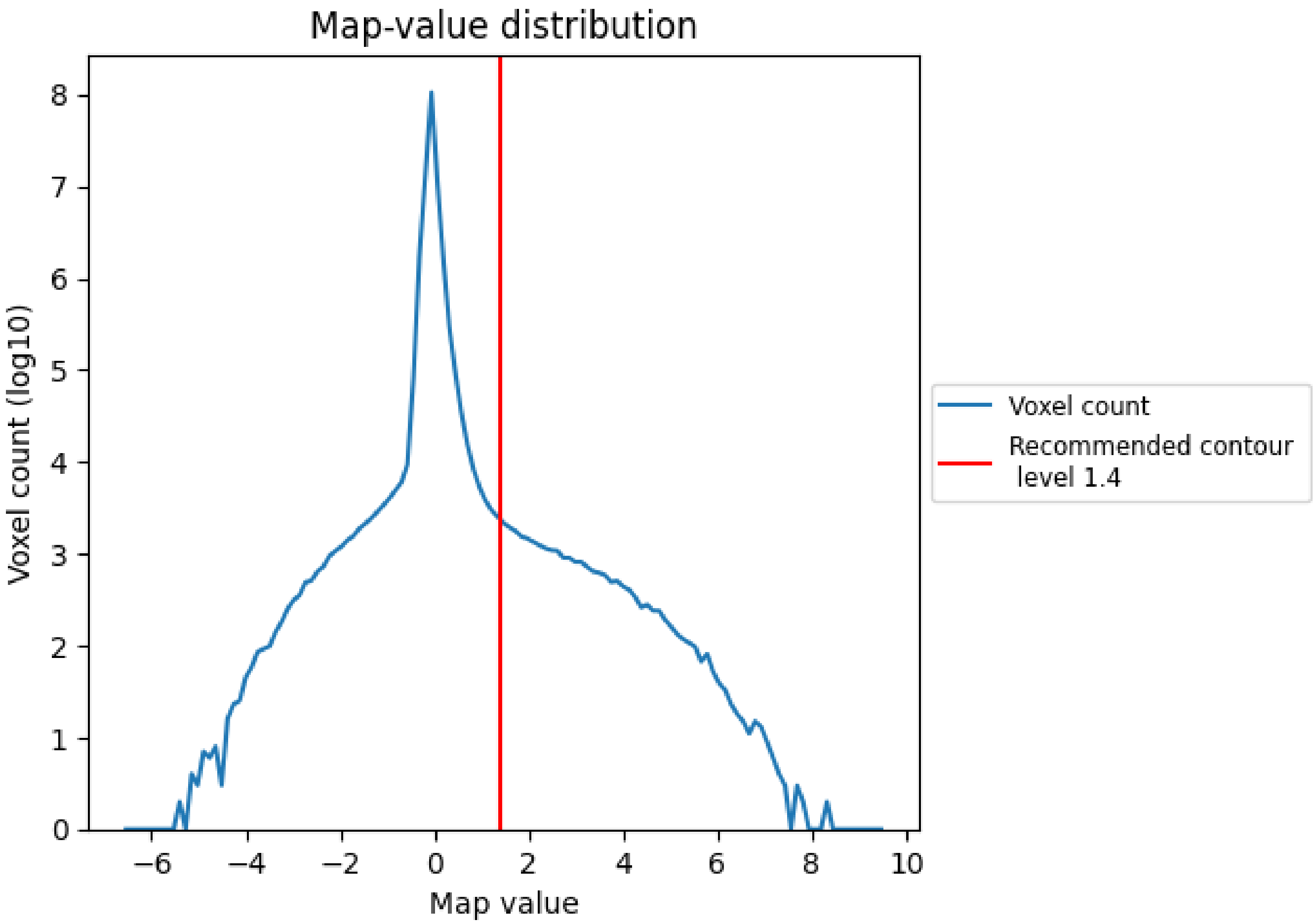

The map-value distribution is plotted in 128 intervals along the x-axis. The y-axis is logarithmic. A spike in this graph at zero usually indicates that the volume has been masked.

For Manuscript Review

7.2 Volume estimate (i)

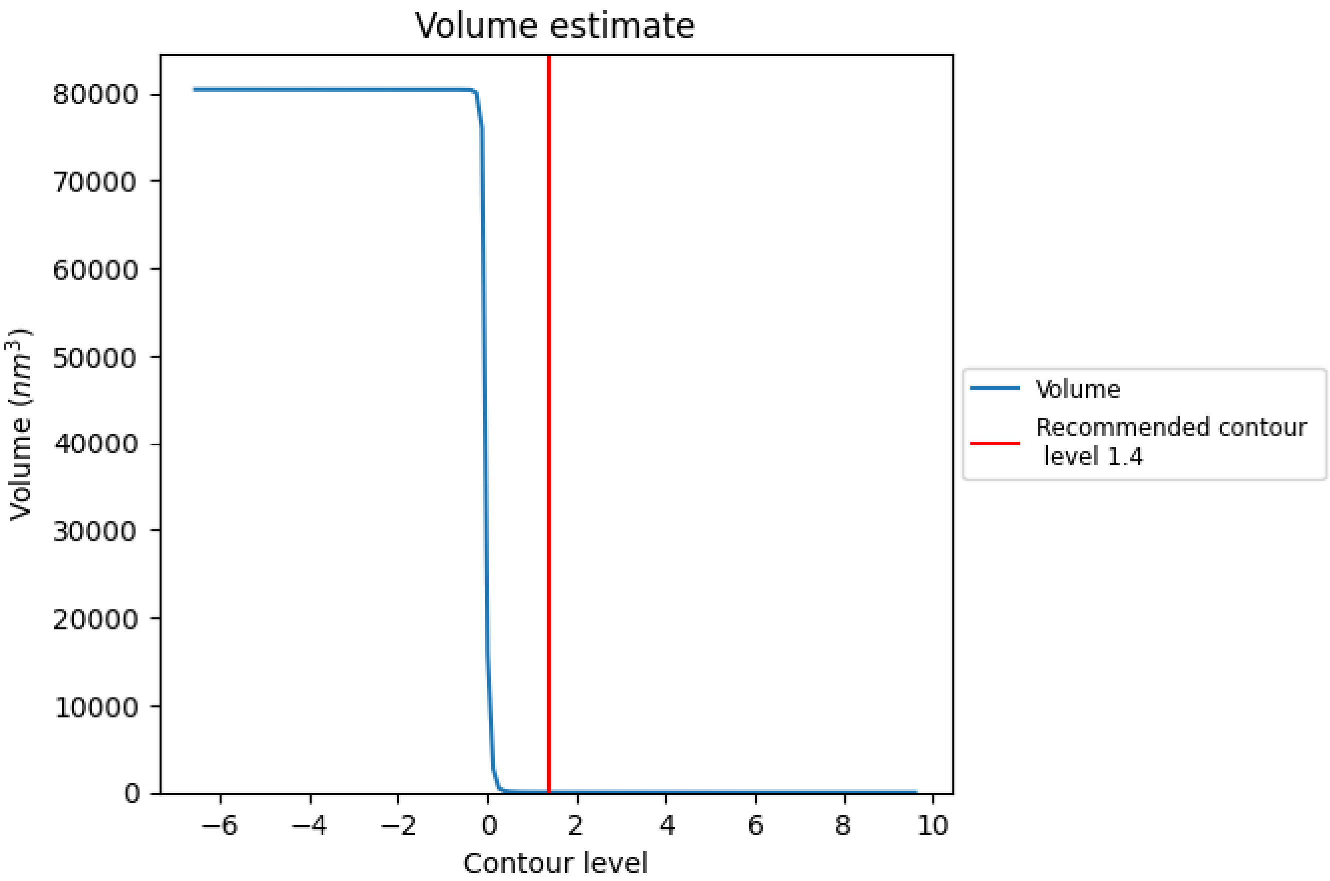

The volume at the recommended contour level is 16 nm^3; this corresponds to an approximate mass of 14 kDa.

The volume estimate graph shows how the enclosed volume varies with the contour level. The recommended contour level is shown as a vertical line and the intersection between the line and the curve gives the volume of the enclosed surface at the given level.

For Manuscript Review

7.3 Rotationally averaged power spectrum (i)

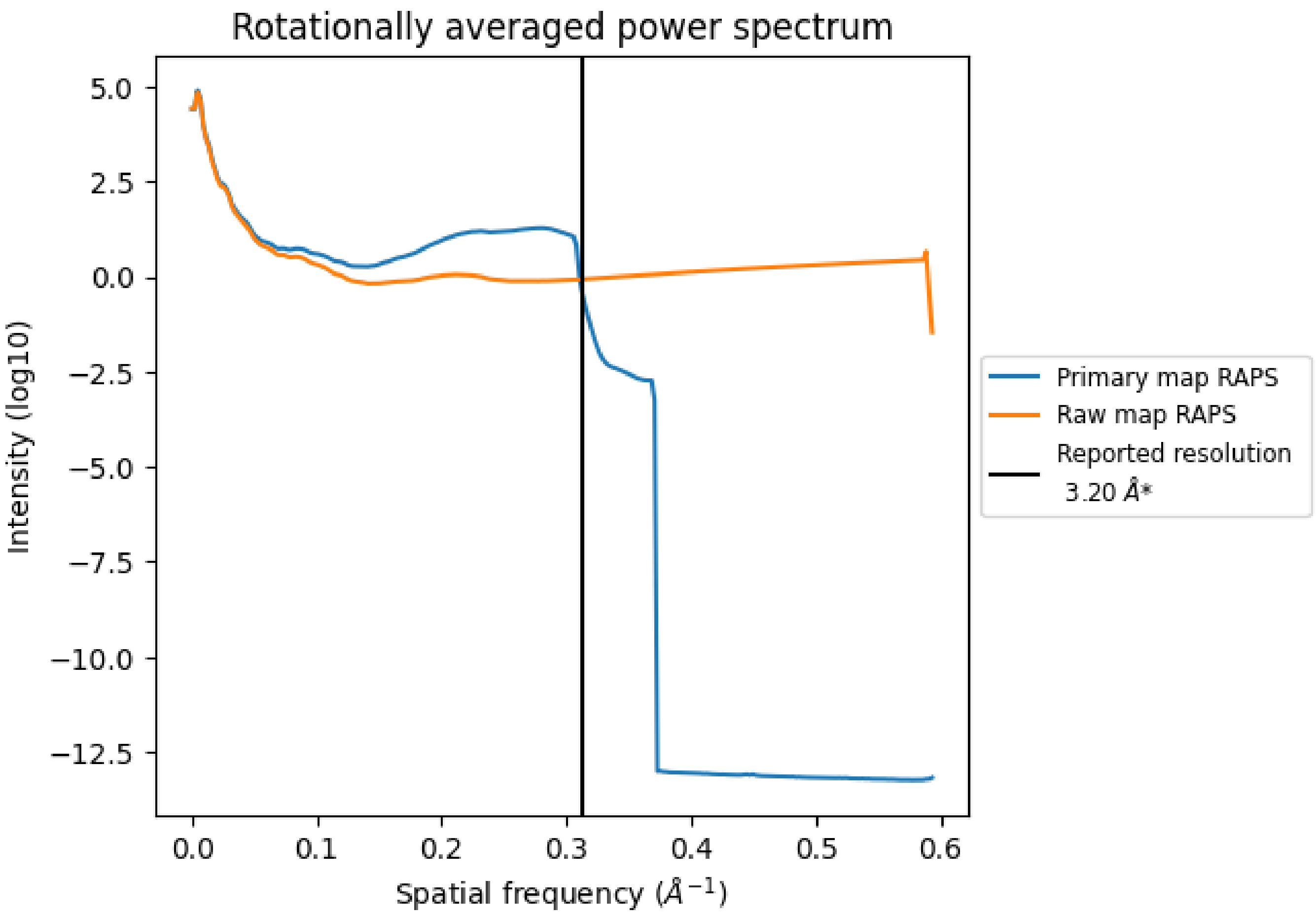

*Reported resolution corresponds to spatial frequency of 0.312 Å^{-1}

For Manuscript Review

8 Fourier-Shell correlation (i)

Fourier-Shell Correlation (FSC) is the most commonly used method to estimate the resolution of single-particle and subtomogram-averaged maps. The shape of the curve depends on the imposed symmetry, mask and whether or not the two 3D reconstructions used were processed from a common reference. The reported resolution is shown as a black line. A curve is displayed for the half-bit criterion in addition to lines showing the 0.143 gold standard cut-off and 0.5 cut-off.

8.1 FSC (i)

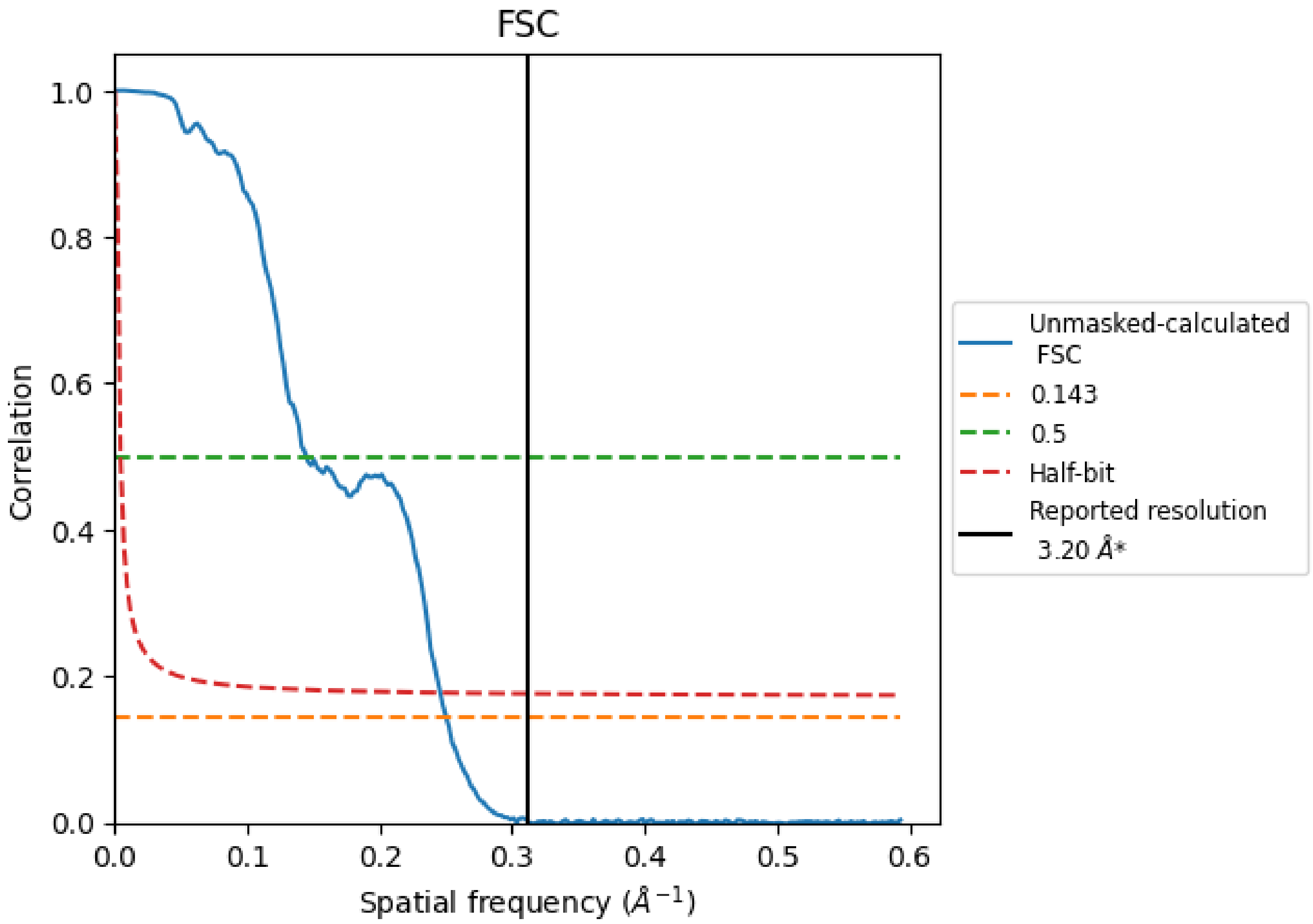

*Reported resolution corresponds to spatial frequency of 0.312 Å^{-1}

For Manuscript Review

8.2 Resolution estimates (i)

Resolution estimate (Å)	Estimation criterion (FSC cut-off)		
	0.143	0.5	Half-bit
Reported by author	3.20	-	-
Author-provided FSC curve	-	-	-
Unmasked-calculated*	3.99	6.91	4.07

*Resolution estimate based on FSC curve calculated by comparison of deposited half-maps. The value from deposited half-maps intersecting FSC 0.143 CUT-OFF 3.99 differs from the reported value 3.2 by more than 10 %

For Manuscript Review

9 Map-model fit (i)

This section contains information regarding the fit between EMDB map EMD-45175 and PDB model 9C45. Per-residue inclusion information can be found in section 3 on page 7.

9.1 Map-model overlay (i)

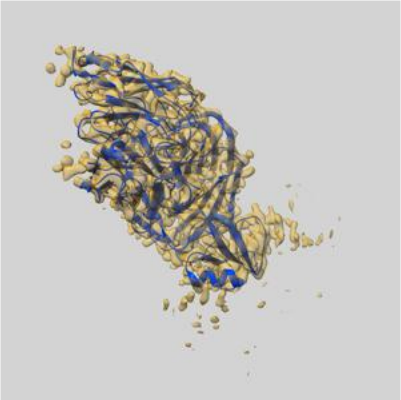

X

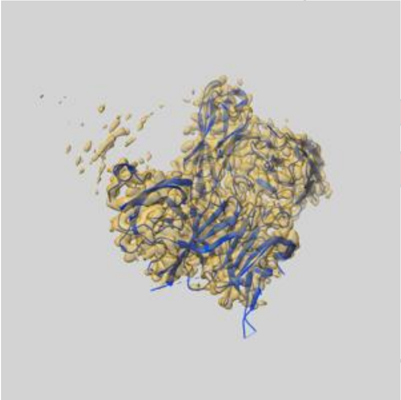

Y

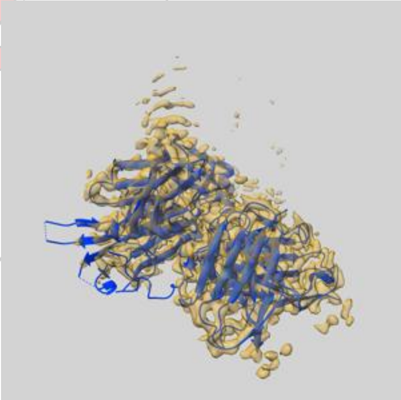

Z

The images above show the 3D surface view of the map at the recommended contour level 1.4 at 50% transparency in yellow overlaid with a ribbon representation of the model coloured in blue. These images allow for the visual assessment of the quality of fit between the atomic model and the map.

For Manuscript Review

9.2 Q-score mapped to coordinate model (i)

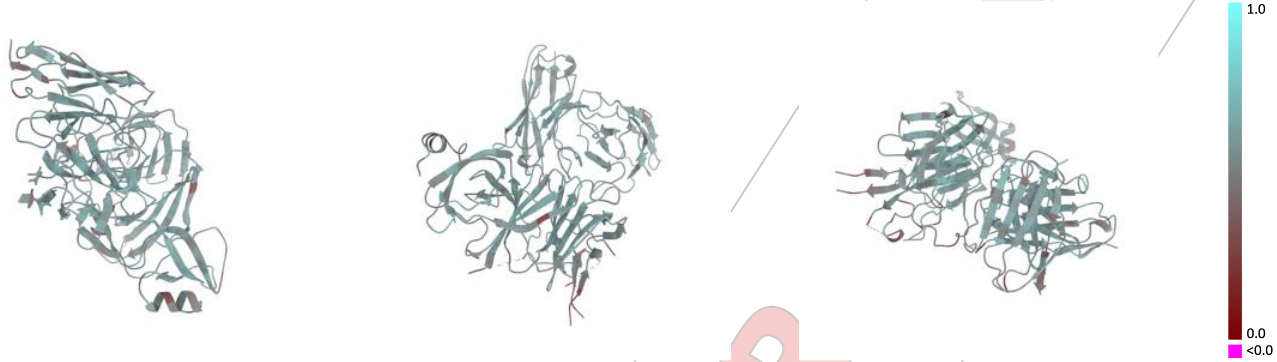

X Y Z

The images above show the model with each residue coloured according its Q-score. This shows their resolvability in the map with higher Q-score values reflecting better resolvability. Please note: Q-score is calculating the resolvability of atoms, and thus high values are only expected at resolutions at which atoms can be resolved. Low Q-score values may therefore be expected for many entries.

9.3 Atom inclusion mapped to coordinate model (i)

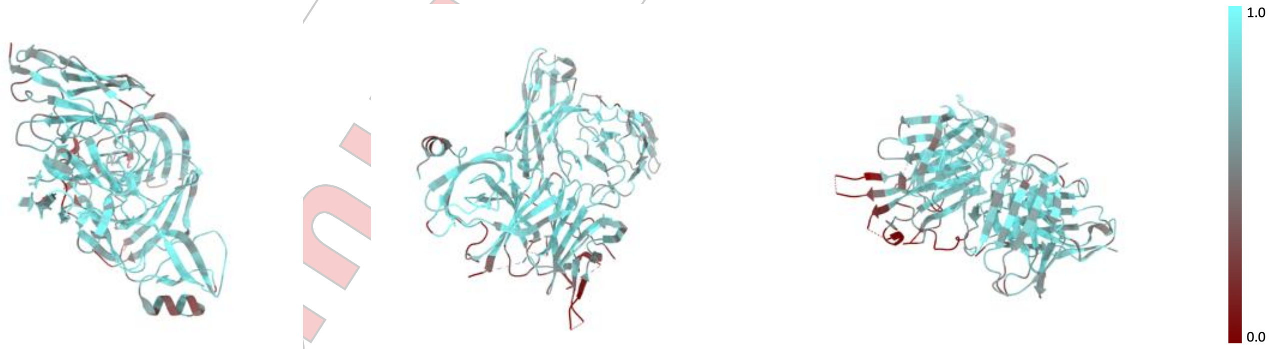

X Y Z

The images above show the model with each residue coloured according to its atom inclusion. This shows to what extent they are inside the map at the recommended contour level (1.4).

For Manuscript Review

9.4 Atom inclusion (i)

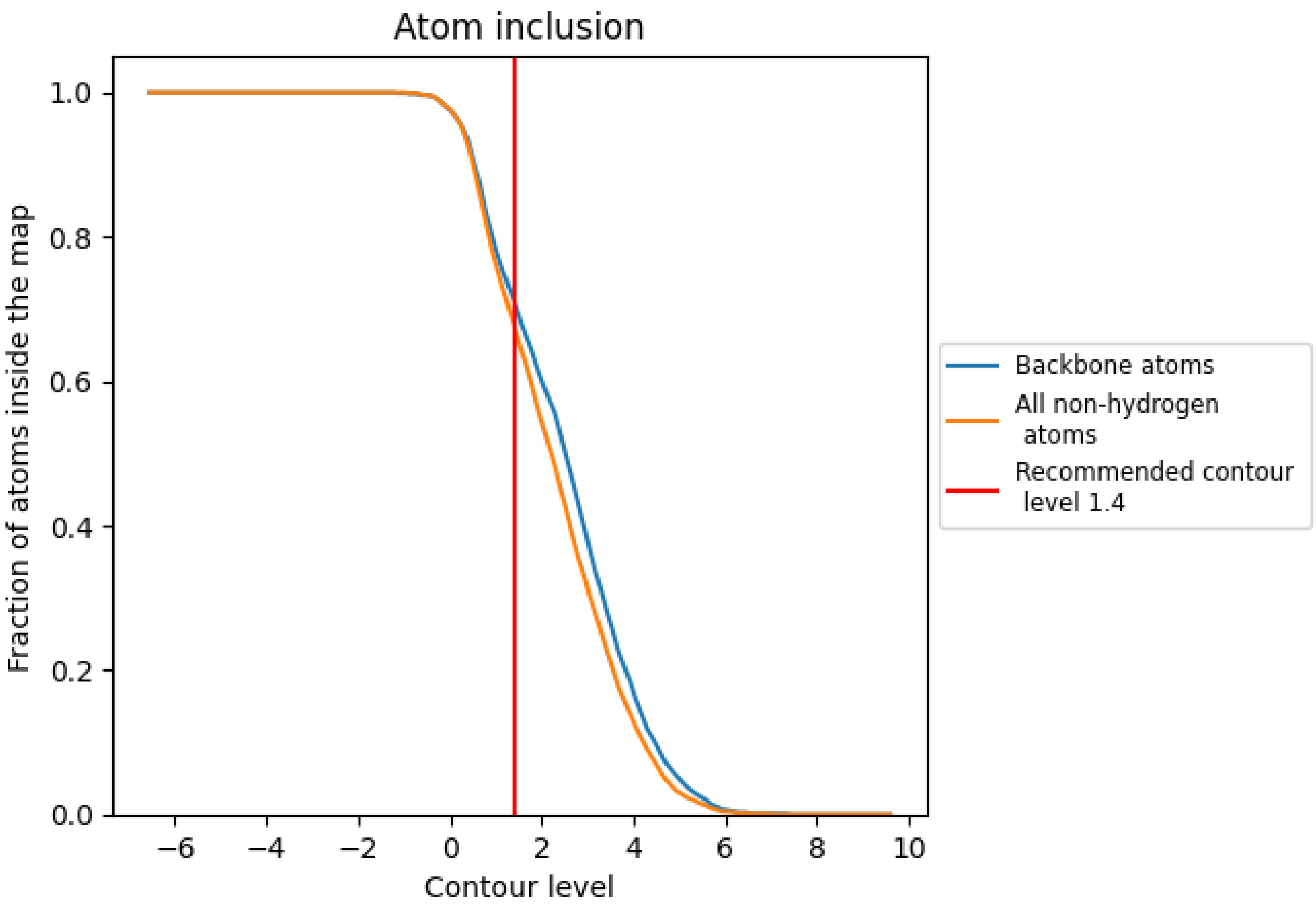

At the recommended contour level, 71% of all backbone atoms, 68% of all non-hydrogen atoms, are inside the map.

For Manuscript Review

9.5 Map-model fit summary (i)

The table lists the average atom inclusion at the recommended contour level (1.4) and Q-score for the entire model and for each chain.

Chain	Atom inclusion	Q-score
All	0.6770	0.5430
A	0.6500	0.5350
B	0.2500	0.4680
C	0.2820	0.4630
H	0.7560	0.5660
L	0.6930	0.5430

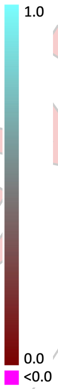

For Manuscript Review
