# Supplementary material for: Design of customized coronavirus receptors
Source: Nature. Author manuscript; Available in PMC 2025 Jun 24. (PMC12187079; doi:10.1038/s41586-024-08121-5)
Supplement: SI guide [file NIHMS2083933-supplement-SI_guide.docx]

**SI guide for Nature_2024-03-04549-B**

The Supplementary Information files include Supplementary Figs. 1-3 in an combined PDF and Supplementary Tables 1-3 in three separate Excel files. Supplementary Figures 1 and 2 present Uncropped immunoblots from Main and Extended Data Figures, respectively; Supplementary Figure 3 describes gating strategies for flow cytometry analysis. Supplementary Tables 1 summarized Viruses and receptors gene information; Supplementary Tables 2 summarized sequences for CVR design; Supplementary Table 3 summarizes information of nanobodies used in this study.
