# Supplementary material for: Design of customized coronavirus receptors
Source: Nature. Author manuscript; Available in PMC 2025 Jun 24. (PMC12187079; doi:10.1038/s41586-024-08121-5)
Supplement: Supp figures 1 to 3 [file NIHMS2083933-supplement-Supp_figures_1_to_3.pdf]

Supplementary information for

Design of customized coronavirus receptors

Peng Liu[1,#], Mei-Ling Huang[1#], Hua Guo[2,#], Matthew McCallum[4,#], Jun-Yu Si[1], Yuan-Mei Chen[1], Chun-Li Wang[1], Xiao Yu[1], Lu-Lu Shi[1], Qing Xiong[1], Cheng-Bao Ma[1], John E. Bowen[4], Fei Tong[1], Chen Liu[1], Ye-hui Sun[1,] Xiao Yang[1], Jing Chen[2], Ming Guo[1], Jing Li[1], Davide Corti[6], David Veesler[4,5,*], Zheng-Li Shi[2,3*], Huan Yan[1,*,&]

[1]State Key Laboratory of Virology, College of Life Sciences, TaiKang Center for Life and Medical Sciences, Wuhan University; Wuhan, Hubei, China.

[2]Key Laboratory of Virology and Biosafety, Wuhan Institute of Virology, Chinese Academy of Sciences; Wuhan, China.

[3]Guangzhou Laboratory, Guangzhou International Bio Island; Guangzhou, China.

[4]Department of Biochemistry, University of Washington; Seattle, WA 98195, USA.

[5]Howard Hughes Medical Institute, University of Washington; Seattle, WA 98195, USA.

[6]Humabs BioMed SA, subsidiary of Vir Biotechnology, Bellinzona, Switzerland.

[#]These authors contributed equally.

[*]Correspondence: e-mail: Huan Yan: huanyan@whu.edu.cn, Zheng-Li Shi: zlshi@wh.iov.cn, David Veesler: dveesler@uw.edu .

[&] Lead contact

Table of content:

SI Figure 1. Uncropped immunoblots used in Main Figures.

SI Figure 2. Uncropped immunoblots used in Extended Data Figures.

SI Figure 3. Gating strategies for representative flow cytometry analyses

SI Table 1. Gene information of viruses and receptors.

SI Table 2. Domain sequences for CVR design.

SI Table 3. Nanobodies information.

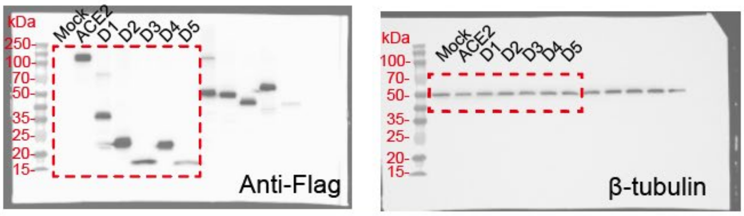

Fig.1b

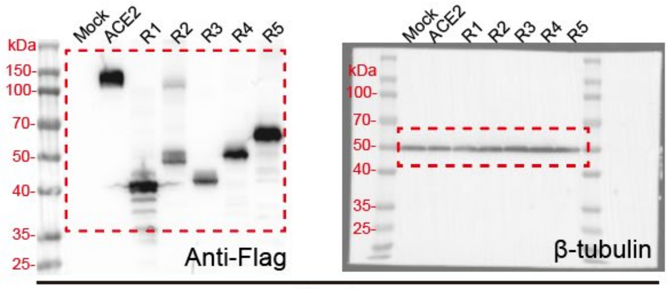

Fig.1g

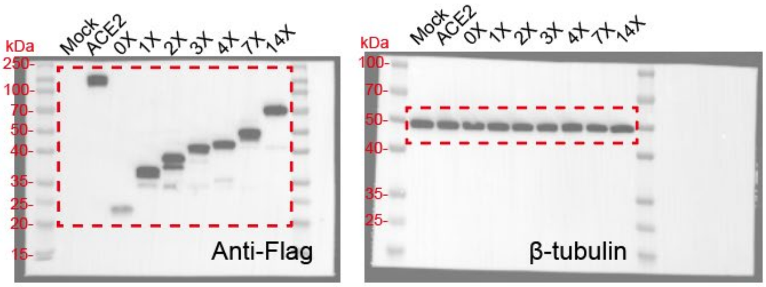

Fig.1l

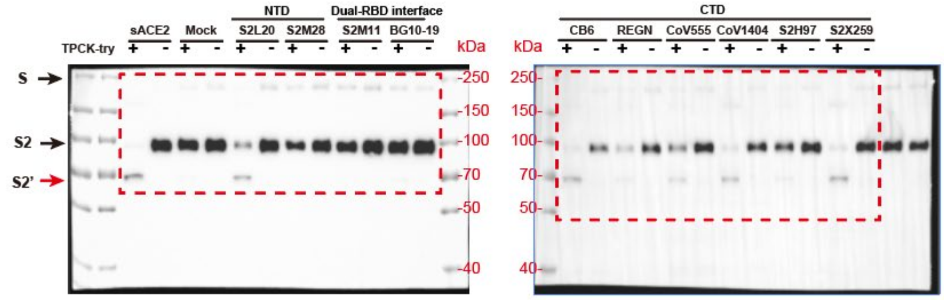

Fig.2h

SI Figure 1. Uncropped immunoblots from Main Figures. Uncropped and unprocessed scans of the protein blots used in Main Figures. Molecular weight markers, detecting antibodies, and the corresponding Figures were indicated.

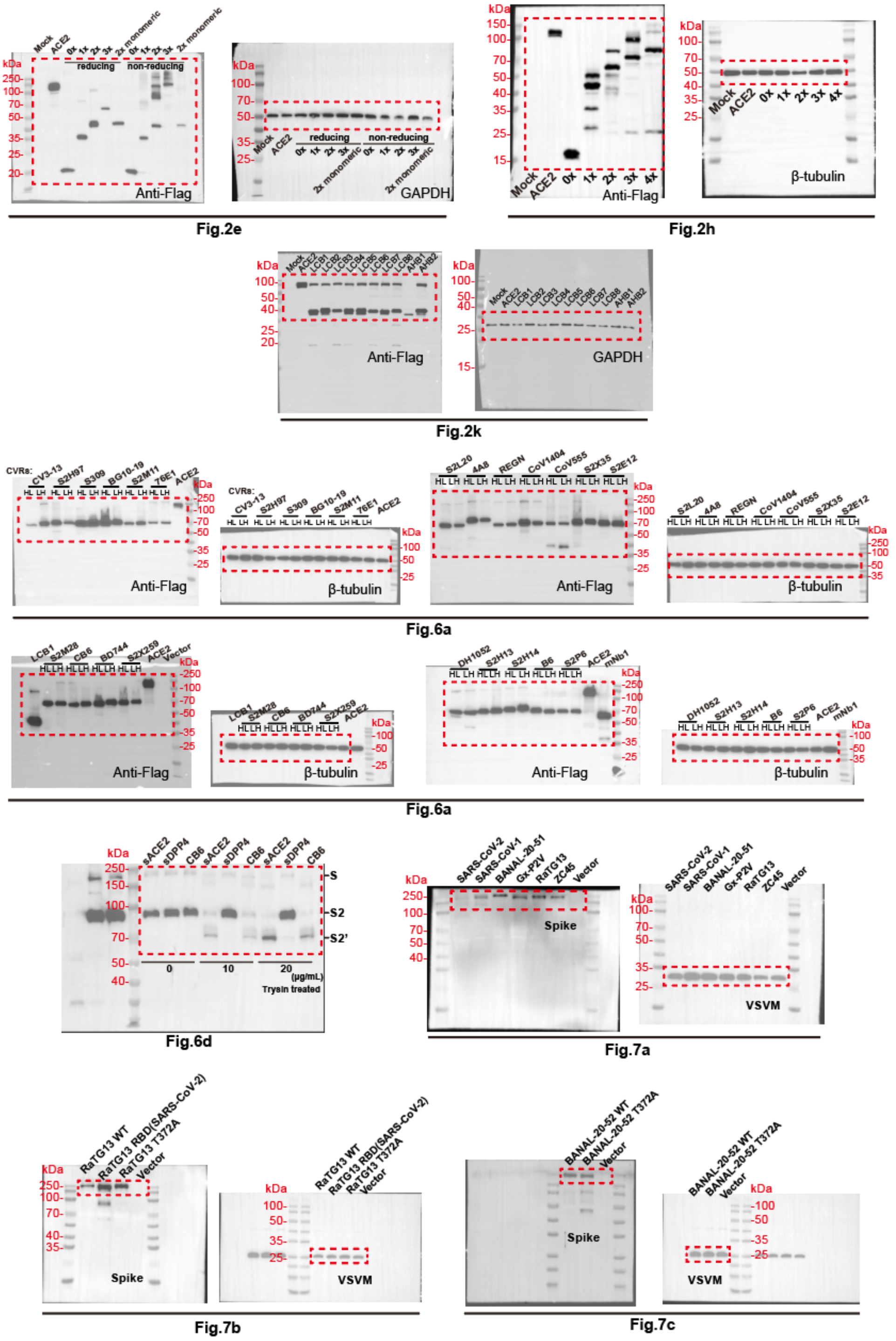

SI Figure 2. Uncropped immunoblots from Extended Data Figures. Uncropped and unprocessed scans of the protein blots used in Extended data Figures. Molecular weight markers, detecting antibodies, and the corresponding Figures were indicated.

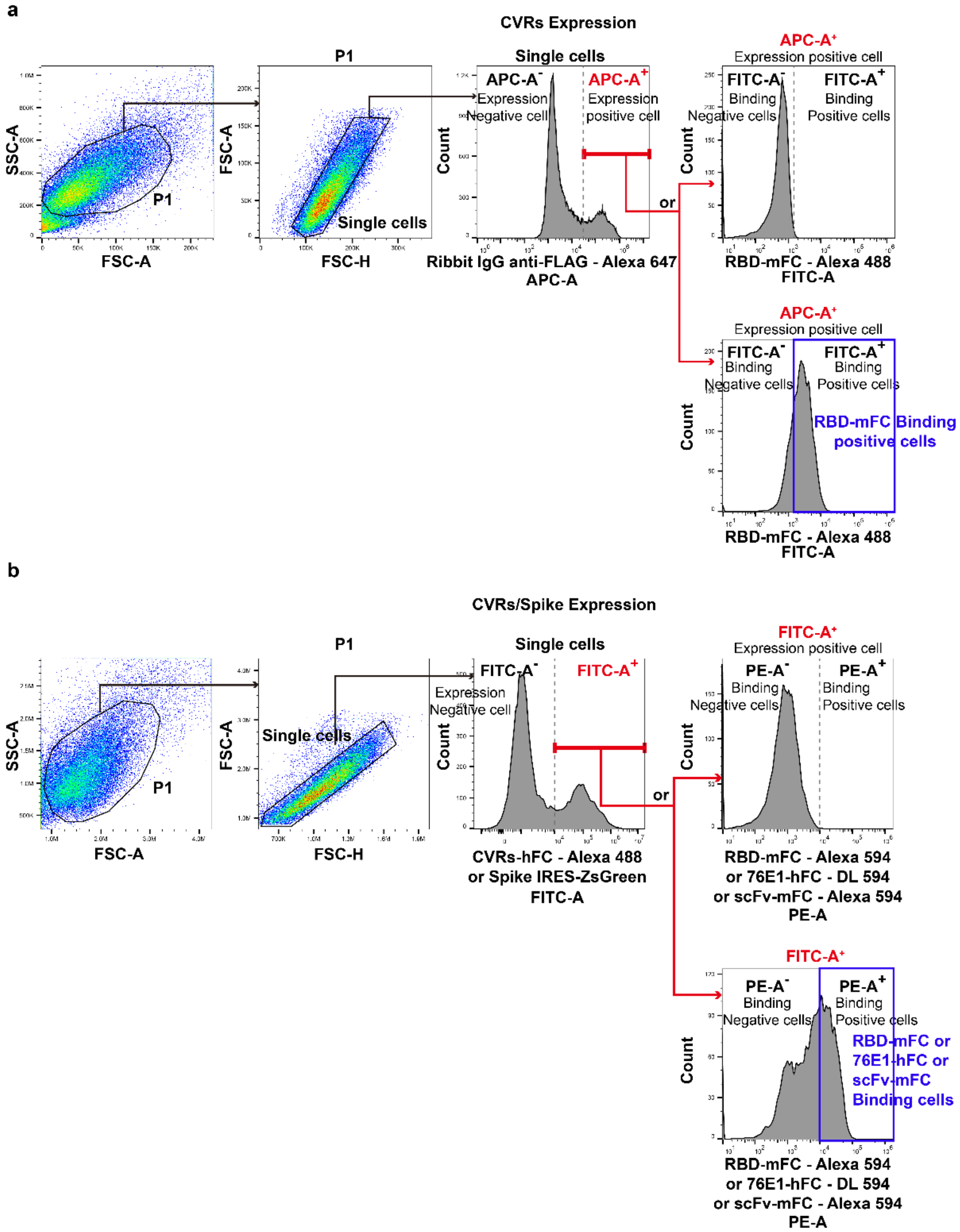

SI Figure 3. Gating strategies for representative flow cytometry analyses. a, SARS2 RBD-mFc binding to ACE2/CVRs expressing HEK293T cells in Figure **1c**, **1h. b,** Antibodies (scFv-mFC or 76E1-hFC) binding to SARS-CoV-2 spike expressing HEK293T cells in Figure **2f**, **2g**, and **Extended data Fig. 6c**. Coronavirus S1/RBD-mFc binding to CVRs expressing HEK293T cells in **Extended data Fig. 8b**. Representative gating to exclude cell debris, dead cells (FSC-A/SSC-A), and doublets (FSC-A/FSC-H), and to select binding positive cells (FITC-A+ or PE-A+) from receptor/spike expressing positive cells (APC-A+ or FITC-A+) based on the threshold set based on the histogram of mock controls (HEK293T transfected with vector plasmids).
